# Fpr1, a primary target of rapamycin, functions as a transcription factor for ribosomal protein genes cooperatively with Hmo1 in *Saccharomyces cerevisiae*

**DOI:** 10.1371/journal.pgen.1008865

**Published:** 2020-06-30

**Authors:** Koji Kasahara, Risa Nakayama, Yuh Shiwa, Yu Kanesaki, Taichiro Ishige, Hirofumi Yoshikawa, Tetsuro Kokubo

**Affiliations:** 1 Department of Molecular Microbiology, Tokyo University of Agriculture, Tokyo, Japan; 2 Department of Bioscience, Tokyo University of Agriculture, Tokyo, Japan; 3 Research Institute of Green Science and Technology, Shizuoka University, Shizuoka, Japan; 4 NODAI Genome Research Center, Tokyo University of Agriculture, Tokyo, Japan; 5 Graduate School of Medical Life Science, Yokohama City University, Yokohama, Japan; University of Geneva, Sciences III, SWITZERLAND

## Abstract

Fpr1 (FK506-sensitive proline rotamase 1**)**, a protein of the FKBP12 (FK506-binding protein 12 kDa) family in *Saccharomyces cerevisiae*, is a primary target for the immunosuppressive agents FK506 and rapamycin. Fpr1 inhibits calcineurin and TORC1 (target of rapamycin complex 1) when bound to FK506 and rapamycin, respectively. Although Fpr1 is recognised to play a crucial role in the efficacy of these drugs, its physiological functions remain unclear. In a *hmo1*Δ (high mobility group family 1-deleted) yeast strain, deletion of *FPR1* induced severe growth defects, which could be alleviated by increasing the copy number of *RPL25* (ribosome protein of the large subunit 25), suggesting that *RPL25* expression was affected in *hmo1*Δ*fpr1*Δ cells. In the current study, extensive chromatin immunoprecipitation (ChIP) and ChIP-sequencing analyses revealed that Fpr1 associates specifically with the upstream activating sequences of nearly all RPG (ribosomal protein gene) promoters, presumably in a manner dependent on Rap1 (repressor/activator site binding protein 1). Intriguingly, Fpr1 promotes the binding of Fhl1/Ifh1 (forkhead-like 1/interacts with forkhead 1), two key regulators of RPG transcription, to certain RPG promoters independently of and/or cooperatively with Hmo1. Furthermore, mutation analyses of Fpr1 indicated that for transcriptional function on RPG promoters, Fpr1 requires its N-terminal domain and the binding surface for rapamycin, but not peptidyl-prolyl isomerase activity. Notably, Fpr1 orthologues from other species also inhibit TORC1 when bound to rapamycin, but do not regulate transcription in yeast, which suggests that these two functions of Fpr1 are independent of each other.

## Author summary

In eukaryotic cells, ribosome production requires the activity of all three RNA polymerases plus vast amounts of energy and resources. Therefore, ribosome production is regulated strictly and in a coordinated manner in response to specific environmental conditions. Fpr1 forms a complex with the immunosuppressive agent rapamycin, and this binary complex inhibits TORC1, a central player in coordinated ribosome production. Fpr1 is known to play a crucial role in drug efficacy, but its involvement in ribosomal biogenesis under physiological conditions remains unclear. *FPR1* mutation has been shown to cause synthetic lethality with mutation of *HMO1*, a widely recognised transcriptional regulator of ribosomal component genes. A previous study demonstrated that the growth defect of *hmo1*Δ*fpr1*Δ cells could be suppressed by increasing the copy number of *RPL25*, an essential RPG, which suggested that decrease of *RPL25* expression caused the growth defect. Here, we found that Fpr1 specifically binds to the promoters of RPGs, including *RPL25*, in a Rap1-dependent manner, and promotes Fhl1/Ifh1 binding to certain RPG promoters. Mutation analyses of Fpr1 revealed that transcriptional regulation by Fpr1 is a physiological process that is unrelated to its non-physiological function in interacting with TORC1. We conclude that Fpr1 is a transcriptional regulator of RPGs.

## Introduction

In eukaryotic cells, ribosome production requires coordinated activities of RNA polymerases I, II, and III and vast amounts of energy and cellular resources [1]. The yeast ribosome is composed of four ribosomal RNAs (rRNAs) and approximately 80 ribosomal proteins [2]. The transcription of the genes encoding distinct ribosomal components is tightly regulated to produce roughly equimolar amounts of the gene products, and TORC1 (target of rapamycin complex 1) plays a central role in coordinating the activities of the three RNA polymerases in response to environmental conditions (such as nutrient availability and various stresses). In rapidly growing yeast cells, TORC1 phosphorylates downstream targets such as Sfp1 and Sch9 to transmit nutrient signals into the nucleus, and this promotes the transcription of rRNAs, ribosomal protein genes (RPGs), and Ribi genes; conversely, starvation signals inhibit TORC1 to switch off the synthesis of ribosomal components in a coordinated manner [3, 4]. In eukaryotic cells, exposure to rapamycin, an immunosuppressive agent originally identified in *Streptomyces hygroscopicus*, mimics starvation conditions and elicits diverse cellular responses, such as reductions in ribosome biogenesis and protein synthesis, induction of autophagy, and exit from the cell cycle [5, 6]. Rapamycin forms a complex with its primary target, FKBP12 (FK506-binding protein 12 kDa), and this binary complex binds to and inhibits TORC1 [5, 7, 8]. FKBP12 was originally identified as a target of FK506, another immunosuppressive agent [9], and the FKBP12-FK506 binary complex has been shown to bind to and inhibit the protein phosphatase calcineurin and thereby prevent T lymphocytes from producing interleukin-2 in mammals [10] or confer cation hypersensitivity to yeast cells [11]. Although the molecular mechanisms by which FKBP12 functions together with FK506 or rapamycin have been elucidated [12], the physiological roles of FKBP12, particularly those independent of these two drugs, remain unclear.

FKBP12 is a member of the FKBP family and is widely conserved among eukaryotes, from yeasts to humans. FKBPs possess peptidyl-prolyl isomerase (PPIase) activity, which catalyses the *cis-trans* isomerisation of peptidyl-prolyl bonds in target proteins and thus contributes to proper protein folding [13]. Increasing evidence suggests that FKBPs are associated with diverse biological processes, some of which are related to various diseases [14, 15]. PPIases are widely conserved among eukaryotes and have been classified into three structurally distinct protein families: cyclophilins, FKBPs, and parvulins [16]. Cyclophilins and FKBPs are non-essential in yeast, and cells lacking one or all of the genes encoding these molecules are viable [16, 17]. *Saccharomyces cerevisiae* contains four FKBPs (Fpr1–4) (Fpr: FK506-sensitive proline rotamase**)**. Fpr1, a yeast orthologue of FKBP12, is smaller than the other FKBPs and appears to lack the characteristic domains other than the FKBP domain. Mammalian FKBP12 modulates the activities of ryanodine receptor, a multimeric Ca^2+^-release channel [15], inositol-1,4,5 triphosphate receptor [15, 18], type I transforming growth factor-β receptor [19, 20], the transcription factor YY1 [21], and palmitoylated H-Ras [22]. In contrast, only a few functions have been reported for Fpr1. Murine Mdr3, a P-glycoprotein multidrug-resistance pump, requires Fpr1 to confer drug sensitivity to the host cells when expressed in yeast [23]. Physiologically, Fpr1 controls the aspartate pathway by regulating aspartokinase [24, 25].

Deletion of *FPR1* causes synthetic lethality with mutation of Hmo1, a yeast high-mobility group box protein [26, 27] that plays diverse roles in the transcription of rRNAs and RPGs. Fpr1 binds to Hmo1 and might regulate Hmo1 dimerization and DNA-binding activities [26]. When bound to RPG promoters, Hmo1 promotes the DNA binding of Fhl1/Ifh1 (forkhead-like 1/interacts with forkhead 1), TFIID, and other general transcription factors [27–32] and thereby activates RPG transcription. Furthermore, Hmo1 might regulate the positions of nucleosomes on RPG promoters by masking and/or looping out specific regions [30, 33, 34] or by cooperating with certain nucleosome remodellers [35]. Dolinski et al. reported that the synthetic lethality of *hmo1*Δ*fpr1*Δ cells could be suppressed by increasing the copy number of *RPL25*, which encodes a component of the ribosomal large subunit, which suggested that Fpr1 participates in RPG transcription together with Hmo1 [26].

Here, we examined how Fpr1 and Hmo1 regulate the transcription of RPGs. Extensive chromatin immunoprecipitation (ChIP) and ChIP-sequencing (ChIP-seq) analyses revealed that Fpr1 specifically binds to the promoters of *RPL25* and other RPGs in a Rap1-dependent manner. The target RPGs of Fpr1 overlap considerably with those of Fhl1 and Rap1, but not Hmo1, which suggests that Fpr1, Fhl1, and Rap1 intimately interact with each other on the target loci. Notably, *FPR1* deletion markedly decreased Fhl1/Ifh1 binding to the promoters of certain RPGs. Most of these RPGs that bind to Fhl1 in a manner facilitated by Fpr1 are deficient in Hmo1 binding and their binding to Fhl1 is negligibly affected by *HMO1* deletion, whereas ~30% of the RPGs bind to Fhl1 independently of both of Hmo1 and Fpr1. These results suggest that Fhl1 binding is regulated by multiple mechanisms at different RPGs: by the known mechanism facilitated by Hmo1, by the newly identified mechanism facilitated by Fpr1, or by other mechanisms involving neither Hmo1 nor Fpr1. The results of further analyses conducted using Fpr1 mutants or orthologues derived from other species (*Schizosaccharomyces pombe* and humans) showed that the PPIase activity of Fpr1 is not required for its function on RPG promoters, and that the newly discovered promoter function of Fpr1 is independent of its other well-characterised function of TORC1 inhibition, which is exerted in the presence of rapamycin. This is the first study to reveal that FKBP12 can function as a transcriptional regulator of RPGs without the involvement of immunosuppressive drugs.

## Results

### Fpr1 specifically binds to RPG promoters

**Fig 1:**
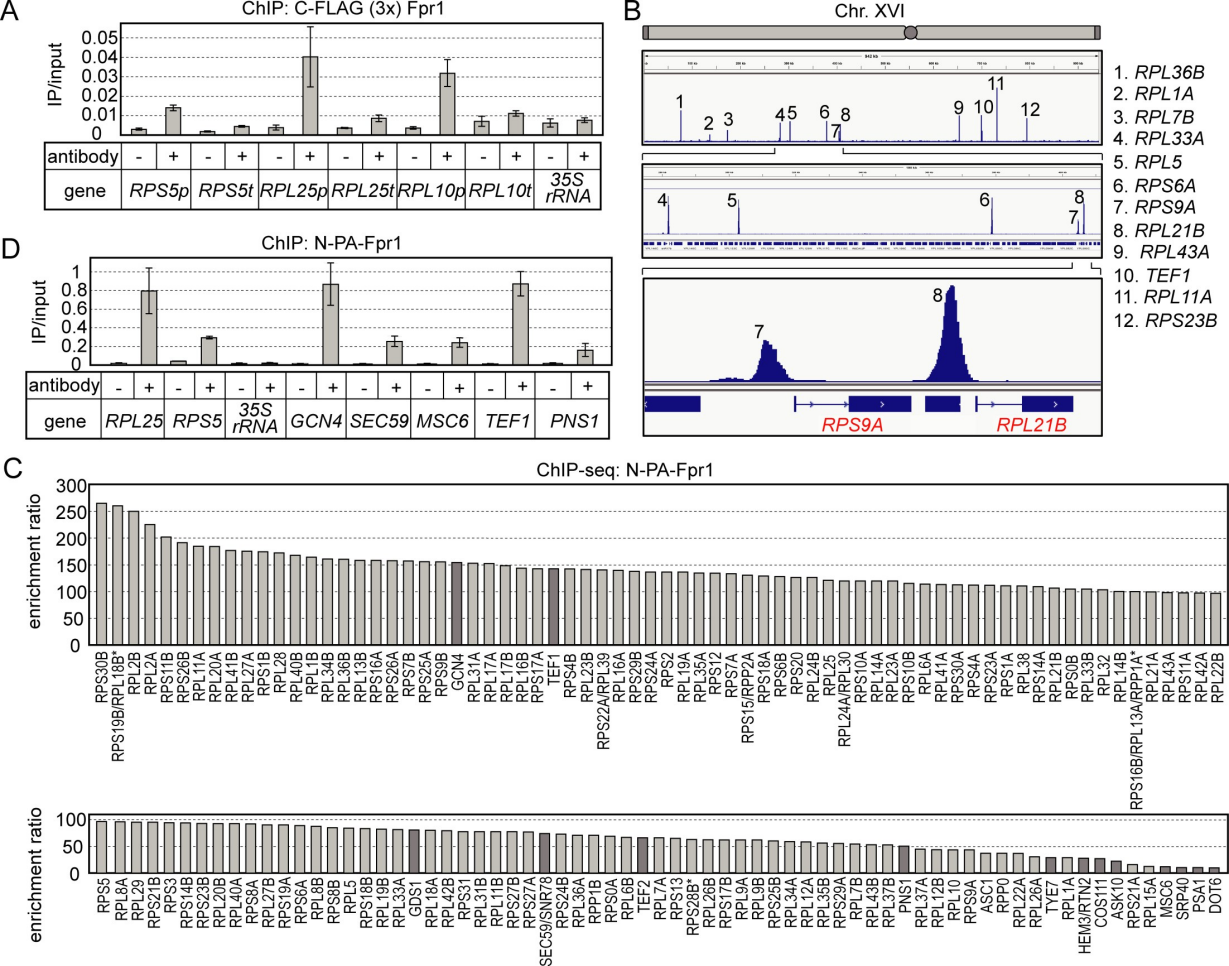
Fpr1 specifically binds to RPG promoters. (A) A ChIP assay was conducted to examine whether Fpr1 binds to RPG promoters *in vivo*. A yeast strain expressing C-terminally FLAG (3×)-tagged Fpr1 (YKK306) was grown in yeast extract-peptone-dextrose medium at 30°C to mid-log phase. IP was conducted using anti-FLAG-tag antibody or no antibody (negative control, indicated as ‘–’). Immunoprecipitated DNAs were subjected to PCR using primer sets to amplify the promoter (p) and 3ʹ-terminal region (t) of *RPS5*, *RPL25*, and *RPL10* ORFs and the promoter of the 35S rRNA gene. The panel summarises the ChIP assay results. (B) Genome-wide ChIP-seq analysis was conducted for Fpr1. Because promoter binding of N-terminally PA-tagged Fpr1 can be detected more efficiently that of C-terminally FLAG (3×)-tagged Fpr1 by ChIP assay, which was used to generate the data shown in panel (A), N-terminally PA-tagged Fpr1 was used for ChIP-seq analysis and all the later ChIP assays. The figure, generated using IGV_2.4.8 software, shows the positions of Fpr1-binding loci. The peaks in the top panel, which are numbered **1**–**12**, represent the Fpr1 binding signals in the entire chromosome (Chr.) XVI. The middle and bottom panels show enlarged views of the parts of the top panel containing peaks **4**–**8** and **7**–**8**, respectively. (C) All target genes of Fpr1 in the entire genome are arranged here in descending order of ChIP-signal values. Pale- and dark-grey bars: RPGs and non-RPGs, respectively; vertical axis: enrichment ratio. (D) Binding of Fpr1 to several non-RPG promoters was verified by means of ChIP assays conducted using yeast strains and the anti-PA-tag antibody used in ChIP-seq analysis.

*HMO1* mutation in the *fpr1*Δ background has been shown to cause lethality [26, 27]. This synthetic lethal phenotype was suppressed by increasing the copy number of *RPL25* [26]. To confirm the synthetic lethality in our study, we generated a *hmo1*Δ*fpr1*Δ double disruptant. Unexpectedly, we found that *hmo1*Δ*fpr1*Δ cells were not lethal, at least in our strain background (S288C and BY4741), but rather showed severe growth retardation as described below. RNA-seq of the *hmo1*Δ*fpr1*Δ cells revealed that among all essential genes, *RPL25* was the most severely affected in terms of transcription by the deletion of *HMO1* and *FPR1* (described below, refer to S5 Table). These findings suggested that Fpr1 may be directly involved in the transcription of *RPL25*, at least in *hmo1*Δ cells. To test this possibility, we conducted ChIP analyses using a yeast strain expressing C-terminally FLAG (3×)-tagged Fpr1 protein. Fpr1 bound weakly, but reproducibly to the promoters of *RPL25* and other RPGs (*RPS5* and *RPL10*), but not to the 35S rRNA gene promoter (Fig 1A). Fpr1 binding to these RPG promoters appeared to be specific as only very weak signals were observed in the absence of antibody (Fig 1A) or when using a strain expressing untagged Fpr1 as a negative control (S1 Fig).

RPG promoters have been classified into two types based on Hmo1 binding levels: Hmo1-enriched and Hmo1-limited [32], or category I and category II [28]. Given that Fpr1 binds to both types of RPG promoters, i.e., Hmo1-limited *RPL10* and Hmo1-enriched *RPL25* promoters, at similar levels, it appears unlikely that Fpr1 binding and Hmo1 binding are correlated.

Previous studies using genome-wide ChIP (ChIP-chip or ChIP-seq) analyses have comprehensively identified the target genes of several well-established transcriptional regulators of RPGs and demonstrated the individual or cooperative functions of these factors [28, 30–32, 36, 37]. To identify Fpr1-target genes on a genome-wide scale, we conducted ChIP-seq analyses for Fpr1 using strains expressing N-terminally PA-tagged Fpr1. Approximately 140 significant Fpr1 binding signals were detected at specific loci throughout the genome (S2 Fig). The binding signals for Fpr1 on chromosome XVI (Chr. XVI) are shown in Fig 1B. We thus identified 12 loci as Fpr1-binding sites, and, unexpectedly, 11 loci overlapped with RPG promoters. All Fpr1-target genes identified by ChIP-seq were aligned in descending order of their ChIP-signal value (enrichment ratio), and are presented in Fig 1C and S3 Table. The top of the list is mostly occupied by RPGs.

Rap1 can bind to various protein-coding genes besides RPGs, such as glycolytic enzyme genes [36], as has been observed for other RPG regulators [32]. However, we mostly identified RPGs as Fpr1 target genes by Chip-seq, except for a very small number of other genes (including *GCN4*, *TEF1*, *GDS1*, *SEC59/SNR78*, *TEF2*, *PNS1*, and *TYE7*; Fig 1C and S3 Table). Individual ChIP assay results verified that Fpr1 binds to these non-RPG target genes at similar levels as to RPGs (Fig 1D) and that these non-RPG promoters are also strongly bound by Rap1 [32]. Collectively, the ChIP and ChIP-seq results suggested that Fpr1 participates in the transcriptional regulation of RPGs and, potentially, a few non-RPGs that are also targeted by Rap1.

### Rap1-dependent Fpr1 binding to RPG promoters

**Fig 2:**
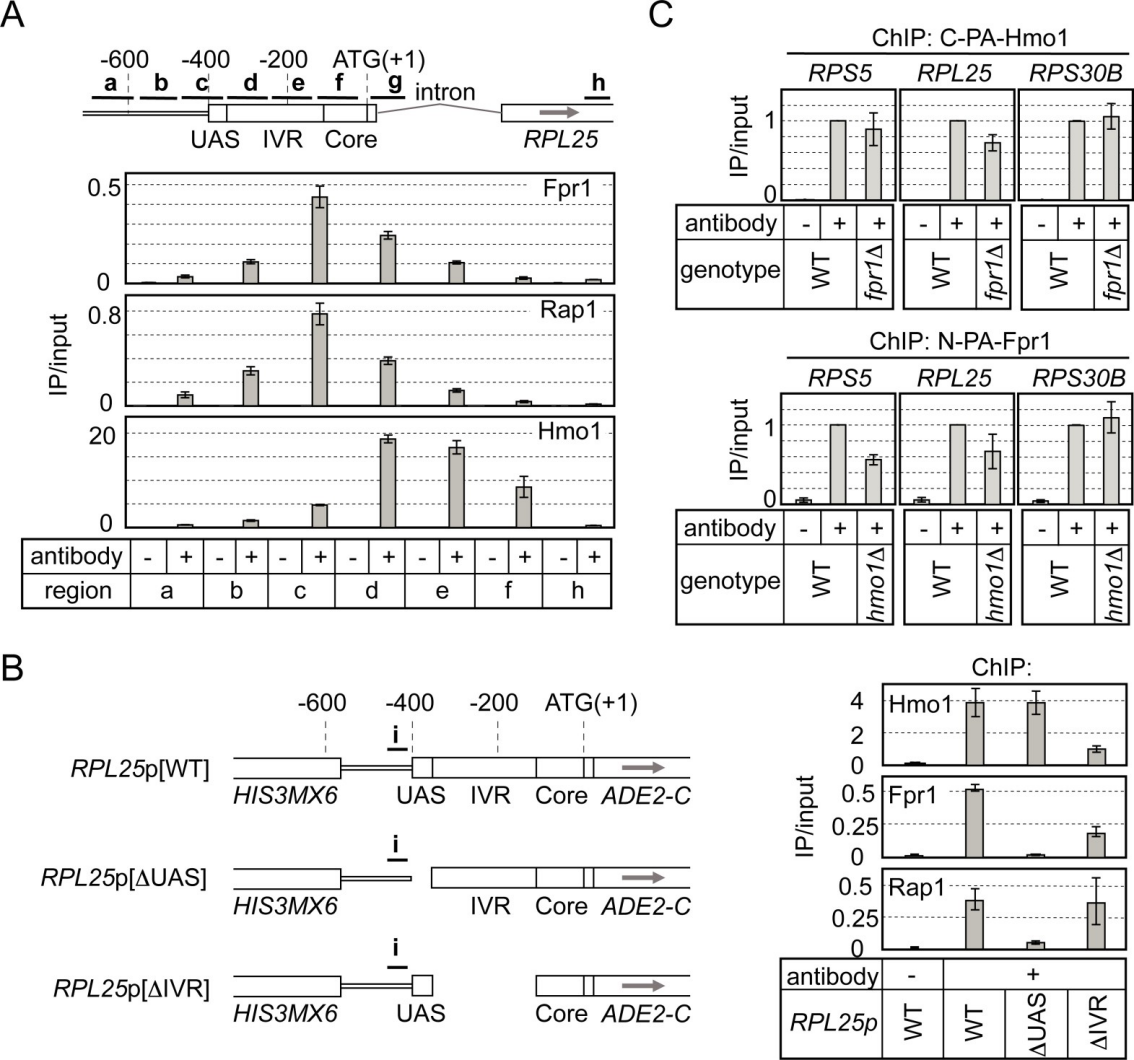
Rap1-dependent binding of Fpr1 to RPG promoters. (A) Detailed mapping of the binding sites of Fpr1, Hmo1, and Rap1 on the *RPL25* promoter, based on a high-resolution ChIP assay. A yeast strain expressing C-terminally FLAG (3×)-tagged Hmo1 and N-terminally PA-tagged Fpr1 (YKK206 harbouring plasmids pKM457 and pKM738) was grown in synthetic dextrose (SD) medium at 30°C to mid-log phase. IP was conducted using anti-PA-tag antibody (Fpr1), anti-FLAG-tag antibody (Hmo1), or anti-Rap1 antibody. A schematic representation of the *RPL25* promoter is shown above the panels summarising the ChIP results for Fpr1, Hmo1, and Rap1; **a**–**h**: regions amplified by PCR in the ChIP assay; UAS, IVR, and Core: upstream activating sequence, intervening region, and core promoter, respectively. (B) To determine the region of the *RPL25* promoter required for Fpr1 binding, a ChIP assay was conducted using modified *RPL25* promoters (depicted on the left). In addition to the wild-type (WT) *RPL25* promoter, UAS/IVR-lacking promoters were integrated into the *ADE2* locus in *fpr1*Δ cells (*RPL25*p[WT]: YKK1054; *RPL25*p[ΔUAS]: YKK1056; *RPL25*p[ΔIVR]: YKK1058). N-terminally PA-tagged Fpr1 was expressed in these strains, and ChIP assays were conducted using anti-PA-tag antibody (Fpr1), anti-Rap1 antibody, or anti-Hmo1 antibody; **i**: region amplified by PCR; top, middle, and bottom panels: summary of ChIP results for Hmo1, Fpr1, and Rap1, respectively. (C) Mutual dependence of Hmo1 and Fpr1 DNA binding was examined by means of ChIP assays conducted using strains expressing C-terminally PA-tagged Fpr1 (*HMO1* (YKK1127) or *hmo1*Δ (YKK1129)) or strains expressing C-terminally PA-tagged Hmo1 (*FPR1* (YKK1123) or *fpr1*Δ (YKK1125)). Values obtained in ChIP assays are expressed as ratios relative to that for the WT strain; results are shown for Fpr1 binding to UAS of promoters of *RPS5*, *RPL25*, and *RPS30B* (lower panels), and Hmo1 binding to the downstream region (upper panels).

Within RPG promoters, Rap1 binds to the upstream activation sequence (UAS), whereas Hmo1 binds to the intervening region (IVR) between the UAS and the core promoter [28, 30, 33]. To compare the binding position of Fpr1 with that of Rap1 and Hmo1 in RPG promoters, we conducted high-resolution ChIP assays for these proteins. The results indicated that Rap1 and Fpr1 bind to the UAS in the *RPL25* promoter, whereas Hmo1 binds to the downstream IVR (~100–200 bp from the Rap1-binding site) of this promoter (Fig 2A). Among the 138 RPGs including *ASC1*, only seven RPGs were found not to have binding sites for Fpr1 (*RPL4A*, *RPL4B*, *RPS22B*, *RPL15B*, *RPL3*, *RPP2B*, and *RPS28A*) (S4 Table). Notably, these RPGs and two other genes, *RPS21A* and *RPL15A*, which are very weakly bound by Fpr1, showed no or weak Rap1-binding signals, respectively, in previous ChIP-chip analyses (S4 Table and [32]). The strong correlation between the binding profiles and the binding positions of Fpr1 and Rap1 suggested that Fpr1 binding to its target RPG promoters might depend on Rap1.

To determine whether Fpr1 binds to its target RPG promoters in a Rap1-dependent manner, we conducted ChIP assays on modified RPG promoters. Briefly, the *RPL25* promoter lacking either IVR or UAS (the Rap1-binding site) was integrated into the *ADE2* locus, as described previously [33], in *fpr1*Δ cells to examine the role of each element in the binding of Fpr1, Rap1, or Hmo1. The results clearly showed that UAS deletion abolished the binding of both Rap1 and Fpr1, which suggested that Fpr1 binding likely depends on Rap1 (Fig 2B). By contrast, Hmo1 binding was affected by deletion of the IVR, but not the UAS (Fig 2B). This result agrees with our previous finding that Hmo1 binding on the *RPS5* promoter was not markedly reduced, even when its UAS was deleted, although this promoter was deficient in Rap1 binding and transcription [33]. However, in contrast to our results, another model has been proposed in which Hmo1 binding to RPG promoters was significantly influenced by deletion of the Rap1-binding motif or by depletion of Rap1 [28, 31]. Although direct evidence to explain this discrepancy is currently not available, we suspect that the marker gene (*HIS3MX6*) that is integrated into the tested locus together with the *RPL25*p[ΔUAS] promoter might create a chromosomal environment (e.g. including a wide nucleosome-free region) within this promoter that leads to Rap1-independent Hmo1 binding. Notably, Fpr1 binding to the *RPL25* promoter lacking the IVR was partially diminished, which suggests that Hmo1 might also play a role in promoting Fpr1 binding to this promoter. However, the ChIP-seq results indicated that Fpr1 binds to most RPG promoters, whereas Hmo1 binds to <70% of the RPG promoters [32], suggesting that Fpr1 binding to the RPG promoters does not necessarily depend on Hmo1. To further investigate this point, we examined the requirement of Hmo1 for Fpr1 binding to DNA in the ChIP assay. The results showed that *HMO1* deletion partially decreased Fpr1 binding to the promoters of *RPS5* and *RPL25* (Hmo1-enriched RPGs), but not *RPS30B* (Hmo1-limited RPG), which suggested that Hmo1 is not essential for DNA binding by Fpr1, but might affect the efficiency of this binding (Fig 2C, lower panels). Thus, Fpr1 is likely recruited by Rap1 to RPG promoters and then stabilized through its interaction with Hmo1 on DNA. As a reciprocal experiment, we examined the requirement of Fpr1 for Hmo1 binding to RPG promoters, which revealed that in the *fpr1*Δ strain, Hmo1 binding to the promoters of *RPS5*, *RPL25* and *RPS30B* was not or only slightly affected (Fig 2C, upper panels). The results of a previous study conducted using a yeast two-hybrid system suggested that Fpr1 could promote Hmo1 dimerization [26], which is required for DNA binding [38]. Although Fpr1 might affect Hmo1 binding to DNA through its interaction with Hmo1, such an effect, if present, might be limited to certain loci (or to nonspecific loci throughout the genome).

### Fpr1 and Hmo1 promote Fhl1/Ifh1 binding to specific RPG promoters cooperatively or independently

**Fig 3:**
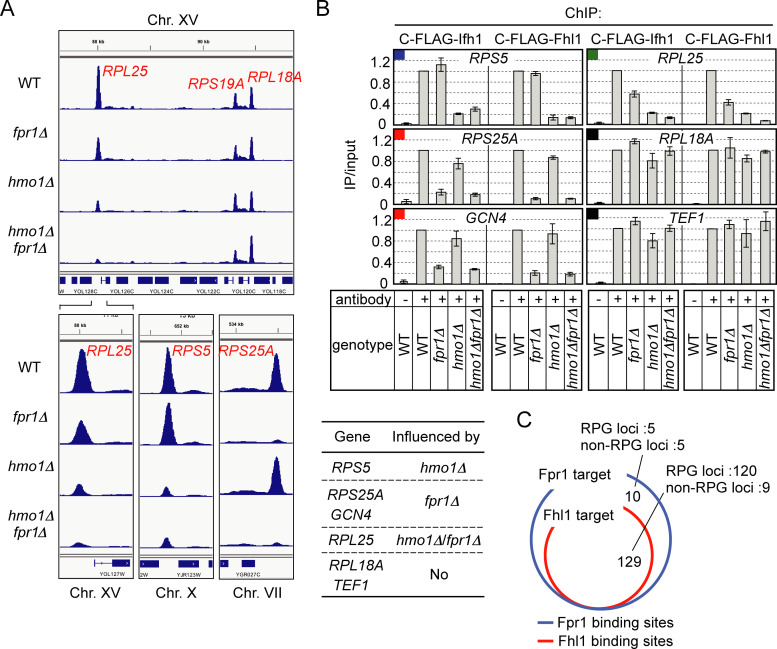
Effect of deletion of *HMO1* and/or *FPR1* on Fhl1/Ifh1 binding to specific RPG promoters. (A) To examine whether Fhl1 binding to RPG promoters is influenced by deletion of *HMO1* and/or *FPR1*, ChIP-seq analyses of Fhl1 were conducted in WT, *fpr1*Δ, *hmo1*Δ, and *hmo1*Δ*fpr1*Δ cells expressing C-terminally FLAG (3×)-tagged Fhl1 (YKK1090, YKK1087, YKK1091, and YKK1086, respectively), as described in the legend for Fig 1B and the Methods section. The binding positions of Fpr1 and Fhl1 in the entire genome are shown in S2 Fig; part of Chr. XV is enlarged and displayed in the upper panel. Binding peaks of Fhl1 at the promoters of *RPL25* (Chr. XV), *RPS5* (Chr. X), and *RPS25A* (Chr. VII) are enlarged and displayed in the bottom panels. (B) Fhl1/Ifh1 binding to certain Fpr1-target promoters in WT, *fpr1*Δ, *hmo1*Δ, and *hmo1*Δ*fpr1*Δ cells was examined by ChIP assays using either the strains listed in (A) or strains expressing C-terminally FLAG (3×)-tagged Ifh1 (YKK1100, YKK1102, YKK1104, and YKK1106). The influence of deletion of *HMO1* and/or *FPR1* on Fhl1/Ifh1 binding to the tested promoters is summarized in a table under the graphs. The coloured symbols at the top of each panel reflect the classification of Fpr1-target genes, as described in S3 Fig. (C) Venn diagram of genes targeted by Fpr1 and Fhl1. The number of target loci corresponding to each sector identified by ChIP-seq is indicated.

Rap1 and Fhl1 bind to >90% of RPG promoters, whereas Hmo1 binds to <70% of RPG promoters [32]. Notably, Fhl1 is recruited to Hmo1-enriched and Hmo1-limited RPG promoters in an Hmo1-dependent and Hmo1-independent manner, respectively [32]. Although it remains unknown how Fhl1 is recruited to Hmo1-limited RPG promoters, the findings that Fpr1 binds to RPG promoters and that *hmo1*Δ*fpr1*Δ cells exhibit a synthetic growth defect raised the possibility that Fpr1 participates in recruiting Fhl1 to RPG promoters. To examine this possibility, we conducted ChIP-seq analysis for Fhl1 in wild-type (WT), *fpr1*Δ, *hmo1*Δ, and *hmo1*Δ*fpr1*Δ cells. We thus identified ~129 loci as binding sites for Fhl1 (S2 Fig and S3 Table). As previously shown [32], Fhl1 binding to the promoter of *RPS5*, an Hmo1-enriched RPG, was significantly decreased in *hmo1*Δ cells, but was unaffected in *fpr1*Δ cells (Fig 3A). Conversely, Fhl1 binding to the promoter of *RPS25A*, an Hmo1-deficient RPG, was significantly decreased in *fpr1*Δ cells, but was unaffected in *hmo1*Δ cells (Fig 3A). Importantly, Fhl1 binding to the promoter of *RPL25*, an Hmo1-enriched RPG, was markedly diminished in both *hmo1*Δ and *fpr1*Δ cells, and these effects were synergistically enhanced in *hmo1*Δ*fpr1*Δ cells (Fig 3A). However, Fhl1 binding to certain RPGs, such as *RPL18A*, was affected to a relatively lesser extent by the deletion of both proteins (Fig 3A, upper panel). The ChIP-seq results of Fhl1 for Fpr1-target genes are summarised S3 Fig.

Next, the effect of deletion of *HMO1* and/or *FPR1* on the binding of Fhl1 and Ifh1 to the Fpr1-target promoters was examined individually using ChIP assays (Fig 3B and S4 Fig). The results largely agreed with those obtained using ChIP-seq (S3 Fig). Fhl1/Ifh1 binding to certain RPG promoters was significantly decreased by deletion of *HMO1*, but not by that of *FPR1* (*RPS5* in Fig 3B, *RPL17A*, *RPL16A*, and *RPL31B* in S4 Fig), whereas the binding to certain other promoters was decreased by deletion of *FPR1*, but not by that of *HMO1* (*RPS25A* and *GCN4* in Fig 3B, *RPS30B*, *RPL35B*, and *RPL10* in S4 Fig). Furthermore, Fhl1/Ifh1 binding to some of the RPGs was decreased significantly (*RPL25* in Fig 3B, *RPS24B*, *RPS16B*, and *RPS0A* in S4 Fig) or slightly (*RPL18A* and *TEF1* in Fig 3B, *RPL41B*, *RPL22B*, and *RPL11B* in S4 Fig) by deletion of both *HMO1* and *FPR1*. In any case, the binding of Fhl1 and Ifh1 to each individual promoter showed nearly identical dependence on Hmo1/Fpr1 (Fig 3B), which is consistent with the widely recognized notion that Fhl1 and Ifh1 form a coactivator complex in rich media [39].

To examine whether the effect of *FPR1* deletion on Fhl1/Ifh1 binding to the RPG promoters was a direct effect, we used the auxin-inducible degron (AID) system [40] to rapidly deplete Fpr1. Fpr1 was C-terminally tagged with a mini AID (codon-optimized for *S*. *cerevisiae*) and expressed from a low-copy-number plasmid under endogenous promoter control in *fpr1*Δ cells. While AID-tagged Fpr1 was lowly expressed in the host strain expressing Tir1, an E3 ubiquitin ligase of *Oryza sativa*, even in the absence of indole-3-acetic acid, AID-tagged Fpr1 became undetectable upon addition of indole-3-acetic acid (0.5 mM, 60 min, S5 Fig A). A ChIP assay using Fpr1-depleted cells demonstrated that Fhl1 binding to the promoters of *RPS25A* and *RPS30B*, both of which bind to Fhl1 in a manner facilitated by Fpr1, was significantly decreased by Fpr1 depletion, whereas Fhl1 binding to the promoter of *RPS5*, which binds to Fhl1 independently of Fpr1, was not affected (S5B Fig). These results suggested that Fpr1 directly promotes Fhl1 binding to certain RPG promoters.

Considering collectively the results of the ChIP and ChIP-seq analyses and those of previous studies on the assembly of Hmo1 and Fhl1, we conclude that Fpr1 is an RPG-specific transcription factor that contributes to the recruitment of Fhl1/Ifh1. In agreement with this view, a Venn diagram for the target genes of Fhl1 and Fpr1 revealed, unexpectedly, that all Fhl1-binding sites, regardless of whether they are present on RPGs or non-RPGs, are included in the binding sites of Fpr1 (Fig 3C).

Based on the ChIP-seq results for Fhl1, Fpr1-target promoters could be classified into several groups and categories (S3 Fig): group **a**: binding of Fhl1 is not influenced by deletion of both *HMO1* and *FPR1* (categories 1 and 2 [black symbol], latter slightly affected in *hmo1*Δ*fpr1*Δ); group **b**: binding of Fhl1 is influenced by deletion of *FPR1*, but not by that of *HMO1* (categories 3 and 4 [red symbol], former unaffected in *hmo1*Δ*fpr1*Δ); group **c**: binding of Fhl1 is influenced by deletion of *HMO1*, but not by that of *FPR1* (categories 5 and 6 [blue symbol], former unaffected in *hmo1*Δ*fpr1*Δ); and group **d**: binding of Fhl1 is influenced by deletion of both *HMO1* and *FPR1* (category 7 [green symbol]). Of note, while deletion of *FPR1* apparently reduced Fhl1 binding to a subset of RPGs (group **b**), most of these RPG promoters retained significant Fhl1 binding with a few exceptions (e.g. *RPS25A* and *RPS30B*), which was also the case for Fhl1 binding upon deletion of *HMO1*. Furthermore, the specific mechanisms underlying the promotion of Fhl1 binding to its target promoters by Hmo1 and Fpr1 are currently unclear. Therefore, here, we preferred to use the term ‘influenced’ instead of ‘dependent’.

As described above, Fpr1 localizes to the UAS (Rap1-binding site) in a Rap1-dependent manner (Fig 2A), whereas Hmo1 binds 100–200 bp downstream from the UAS (Fig 2A). By contrast, comparison of the ChIP-seq results for Fpr1 and Fhl1 revealed that Fhl1 was positioned slightly downstream (~50–100 bp) from Fpr1 (results obtained for *RPS5*, *RPL25*, and *RPS25A* are shown in S6 Fig; compare red dashed line [binding peak of Fpr1] and blue dashed line [binding peak of Fhl1]). Previous studies conducted to determine the precise positions of RPG regulators by using high-resolution ChIP-seq analysis (ChIP-exo) showed that Hmo1 localizes ~110–160 bp downstream from the Rap1-binding site in Hmo1-enriched RPGs (IFHL motif), whereas Fhl1 (or FIS:Fhl1-Ifh1-Sfp1 complex) localizes at the FHL1 motif between Rap1- and Hmo1-binding sites (~100 bp downstream from the Rap1 site) [30]. Our results largely agree with this arrangement, and further reveal a binding position on these promoters for Fpr1, a newly identified regulator of RPGs. Notably, while *HMO1* deletion decreased Fhl1 binding to Hmo1-enriched RPG promoters [31, 32], it also caused an upstream shift of the Fhl1-binding position specifically on Hmo1-enriched RPG promoters (*RPS5* and *RPL25*; compare binding peaks of Fhl1 between WT and *hmo1*Δ cells in S6 Fig). Together with previous findings showing that *HMO1* deletion affects the positions of preinitiation complex (PIC) assembly and the +1 nucleosome [30, 33], our results suggest that Hmo1 plays a central role in organising a complex that includes transcriptional regulators, general transcription factors, and nucleosomes on promoters.

Considering that rapamycin binds to FKBP12 to inhibit TORC1, we suspected that rapamycin might also affect the function of Fpr1 as an RPG transcriptional regulator. To examine this possibility, we conducted ChIP assays for Fpr1 and Fhl1 using a strain expressing both of N-terminally PA-tagged Fpr1 and C-terminally FLAG-tagged Fhl1. The cells were treated with rapamycin (80 nM or 400 nM). The results demonstrated that neither Fhl1 binding (consistent with [37, 39, 41, 42]) nor Fpr1 binding to the RPG promoters was affected by rapamycin treatment (S7 Fig). These results suggested that the rapamycin-bound form of Fpr1 could bind to DNA and recruit Fhl1 to their target genes.

### Effect of *HMO1* and/or *FPR1* deletion on transcription

**Fig 4:**
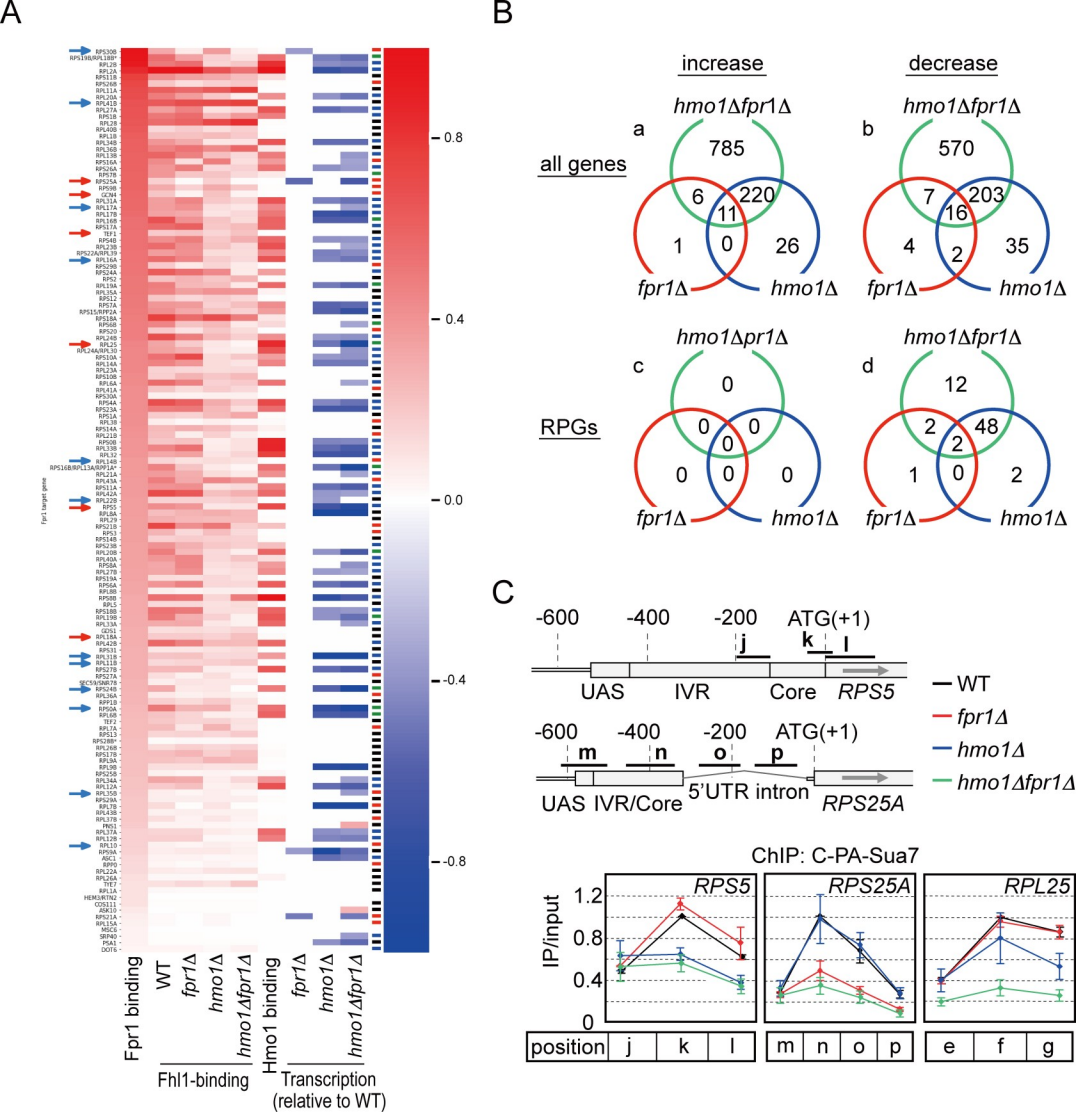
Effect of *HMO1* and/or *FPR1* deletion on transcription. (A) RNA levels of Fpr1-target genes were determined by RNA-seq analyses; the calculated ratios relative to WT were arranged in descending order of Fpr1-binding values and are shown as a heatmap, together with the ChIP-seq results for Fhl1. The Hmo1-binding results are ChIP-seq data quoted from Reja et al. [30]. Coloured symbols in the heatmap indicate that Fhl1 binding to each RPG is not influenced by deletion of *HMO1* and *FPR1* (black); is influenced by deletion of *FPR1*, but not by that of *HMO1* (red); is influenced by deletion of *HMO1*, but not by that of *FPR1* (red); or is influenced by deletion of both *HMO1* and *FPR1* (green), as indicated in S3 Fig. The red and blue arrows on the left indicate the RPGs that were individually examined using ChIP assays, shown in Fig 3B and S4 Fig, respectively. (B) Venn diagrams of genes whose transcription was decreased or increased by *HMO1* and/or *FPR1* deletion. Upper and lower Venn diagrams: genes in the entire genome and RPGs, respectively. The numbers of genes corresponding to each sector identified using RNA-seq are indicated. (C) Effect of *HMO1* and/or *FPR1* deletion on PIC assembly on specific RPG promoters, examined using ChIP assays in WT, *fpr1*Δ, *hmo1*Δ, and *hmo1*Δ*fpr1*Δ cells expressing C-terminally PA-tagged Sua7 (YKK1115, YKK1117, YKK1119, and YKK1121, respectively). Schematic diagrams: promoters of *RPS5* and *RPS25A*; **j–l** (*RPS5*) and **m–p** (*RPS25A*): regions amplified by PCR in the ChIP assay. A schematic representation of the *RPL25* promoter indicating the regions amplified by PCR is depicted in Fig 2A (**e**, **f**, **g**). Values obtained from ChIP assays were expressed as ratios to the top value of WT cells and are shown as line graphs.

To examine how deletion of *HMO1* and/or *FPR1* affects genome-wide transcription, we performed RNA-seq analyses using WT, *fpr1*Δ, *hmo1*Δ, and *hmo1*Δ*fpr1*Δ cells; the results are listed in S5 Table and graphically displayed as heatmaps in S8 Fig (entire genome) and Fig 4A (Fpr1-target genes). Little correlation appeared to exist between the amounts of Fpr1 binding and Hmo1 binding, which agrees with the finding that the target genes of Fpr1 and Hmo1 are not necessarily correlated, as described above (Fig 4A). *HMO1* deletion caused a decrease or increase in the mRNA levels of several genes across the genome (Fig 4B; increase: 257; decrease: 256), whereas *FPR1* deletion affected only a few genes (Fig 4B; increase: 18; decrease: 29). Fpr1 bound to most RPG promoters together with Rap1 and Fhl1 (S4 Table), and *FPR1* deletion significantly affected Fhl1 binding to 37 RPGs (red or green symbol in S3 Fig), whereas its effect on RPG transcription was limited to a few genes (*RPS25A*, *RPS30B*, *RPS21A*, *RPS9A*, and *RPS22B* [*RPS22B* was not bound by Fpr1]) (Fig 4A). These results are consistent with the finding that *FPR1* deletion only slightly affected cell growth as compared with *HMO1* deletion ([26] and our results described below). Notably, however, *FPR1* deletion in the *hmo1*Δ background severely affected genome-wide transcription (Fig 4B and S8 Fig). Given that Fpr1 did not bind to most of the affected genes, these effects might be largely indirect, although the precise mechanisms underlying this genome-wide increase and decrease in transcription remain unclear. By contrast, the transcription of certain RPGs was markedly decreased, whereas that of none of the RPGs was increased in *hmo1*Δ*fpr1*Δ cells (and in other tested mutants) as compared with the transcription in WT cells (Fig 4B, Venn diagram **c**, and S6 Table). Considering this result together with the finding that Hmo1 and Fpr1 directly bind to the aforementioned RPGs, we expect the effect of *HMO1* and/or *FPR1* deletion on RPG transcription to be direct. Importantly, the transcription of RPGs was significantly decreased in *hmo1*Δ*fpr1*Δ cells in terms of the types of RPGs that were transcribed and the amount of RPG transcripts produced, as compared with that in the WT and other strains (Fig 4B, Venn diagram **d**, and S6 Table), which suggests that Fpr1 promotes the transcription of certain RPGs cooperatively with Hmo1. Notably, the RNA-seq results demonstrated that *RPL25* was the most severely affected in terms of transcription by deletion of *HMO1* and *FPR1* among all essential genes (S5 Table). This finding strongly suggested that the severe effect on *RPL25* transcription likely is responsible for the growth restriction in *hmo1*Δ*fpr1*Δ cells, which can also explain why the growth defect of these cells was substantially mitigated by duplication of a single gene, *RPL25*.

We next evaluated the relationship between the effect of *HMO1*/*FPR1* deletion on Fhl1 binding and on transcription by clustering Fpr1-target genes according to their Fhl1-binding profile (S9 Fig). The result clearly demonstrated that Fhl1 binding to RPGs exhibiting abundant Hmo1 binding is significantly decreased by deletion of *HMO1*, and that transcription of these RPGs is also decreased by deletion of *HMO1* (groups **c** and **d**, blue and green symbols, respectively in S3 and S9 Figs). By contrast, the amount of Fpr1 binding did not correlate with the degree of decrease in transcription induced by *FPR1* deletion (Fig 4A). The RPGs whose binding to Fhl1 was influenced by deletion of *FPR1*, but not by that of *HMO1* (group **b**, red symbol in S9 Fig) showed no or only limited Hmo1 binding, and their transcription was not influenced by deletion of *HMO1* (S9 Fig). Other RPGs whose binding to Fhl1 was not influenced by deletion of both *HMO1* and *FPR1* (group **a**, black symbol in S9 Fig) also showed limited Hmo1 binding and slight influence of deletion of *HMO1* on transcription (S9 Fig). These results suggested that nearly half of all RPGs exhibit abundant Hmo1 binding and require Hmo1 for efficient Fhl1 binding and transcription, whereas the other RPGs, showing no or only limited Hmo1 binding, use Fpr1 or other factors rather than Hmo1 to promote Fhl1 binding and transcription.

Our previous study showed that PIC amounts and positions on Hmo1-enriched RPG promoters were affected by *HMO1* deletion [33]. To test whether *FPR1* deletion also would affect PIC assembly on its target promoters, we examined the binding of Sua7 (yeast TFIIB) to the three RPG core promoters by ChIP assays using WT, *hmo1*Δ, *fpr1*Δ, and *hmo1*Δ*fpr1*Δ cells (Fig 4C). As expected from the results of ChIP assays for Fhl1/Ifh1 (Fig 3B) and RNA-seq (S6 Table), Sua7 binding, which reflects PIC assembly, to the *RPS5* core promoter was markedly decreased in *hmo1*Δ cells, but not *fpr1*Δ cells (Fig 4C). Furthermore, as previously reported [33], the Sua7-binding positions on the *RPS5* core promoter were shifted upstream in *hmo1*Δ and *hmo1*Δ*fpr1*Δ cells (Fig 4C; Sua7 binds most abundantly to region **k** in WT and *fpr1*Δ cells, whereas it binds to regions **j** and **k** at similar and significantly higher levels than to the region **l** in *hmo1*Δ and *hmo1*Δ*fpr1*Δ cells). By contrast, Sua7 binding to the Hmo1-limited *RPS25A* core promoter was significantly decreased in *fpr1*Δ cells, but not *hmo1*Δ cells (Fig 4C), which agrees well with the RNA-seq results (S6 Table). Notably, the Sua7-binding position on the *RPS25A* promoter was not shifted in *fpr1*Δ cells, which suggested that Fpr1, unlike Hmo1, does not function in directing PIC assembly at appropriate positions on its target promoters. Lastly, we found that deletion of *FPR1* alone only modestly affected Sua7 binding to the *RPL25* promoter and *RPL25* RNA levels (Fig 4C and S6 Table), although it markedly decreased Fhl1/Ifh1 binding to this promoter (Fig 3B). Similarly, *FPR1* deletion significantly affected Fhl1 binding to the promoters of numerous other RPGs (group **b**, red symbol in S9 Fig), but only modestly affected their transcription (S6 Table and S9 Fig). Most of these genes exhibited diminished yet notable levels (~50% of WT) of Fhl1 binding (S3 Fig). Thus, Hmo1 and/or other factors, such as Rap1, could potentially compensate to ensure sufficient transcription of these genes either by recruiting minimal levels Fhl1/Ifh1 or by acting through an Fhl1/Ifh1-independent mechanism, such as one involving direct interactions among Hmo1, Rap1, and TFIID [29, 43].

### Characterization of Fpr1 function

**Fig 5:**
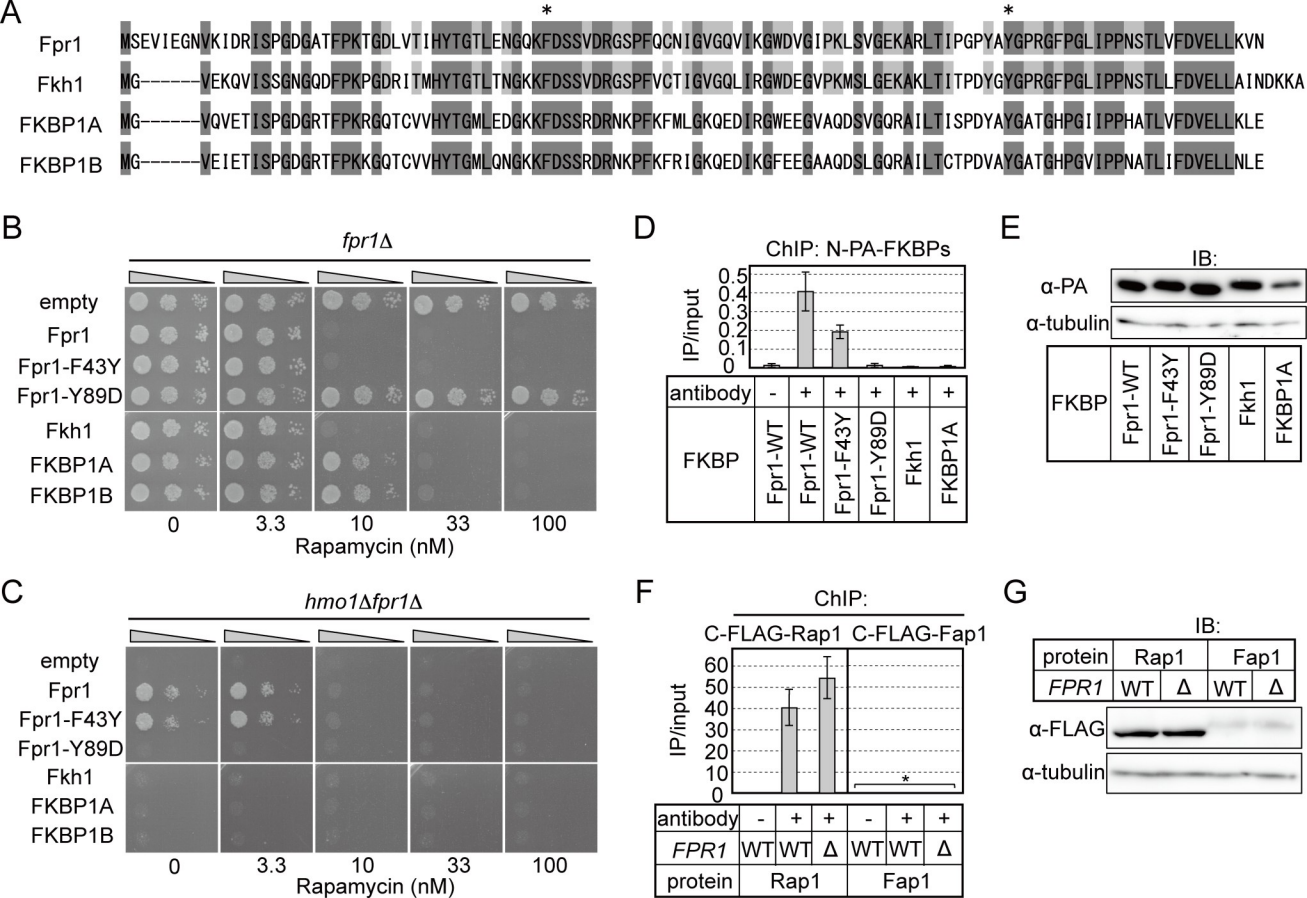
Effect of mutations of Fpr1 on its functions. (A) Alignment of Fpr1 and its orthologues from other species. Amino acids highlighted in dark grey are conserved among Fpr1, Fkh1 (*S*. *pombe*), FKBP1A (human), and FKBP1B (human). Amino acids highlighted in pale grey are conserved between Fpr1 and Fkh1. (B) WT or mutant Fpr1 proteins (F43Y, Y89D) or Fpr1 orthologues from other species were expressed in *fpr1*Δ cells (YKK207 cells harbouring plasmids expressing Fpr1^WT^ (pKM738), Fpr1^F43Y^ (pKM769), Fpr1^Y89D^ (pKM788), Fkh1 (pKM774), FKBP1A (pKM794), or FKBP1B (pKM785)). The resulting strains were spotted onto SD plates containing or not containing rapamycin as indicated, and grown at 30°C for 3 days. (C) The activity of Fpr1 proteins or Fpr1 orthologues in enhancing *hmo1*Δ*fpr1*Δ cell growth was examined. This activity should reflect the function of the proteins in promoting the transcription of Fpr1-target genes. The same proteins as in (B) were expressed in *hmo1*Δ*fpr1*Δ cells (YKK206), and the resulting strains were spotted onto SD plates containing or not containing rapamycin as indicated, and grown at 30°C for 4 days. (D) ChIP analysis of DNA binding of Fpr1 proteins and orthologues. The strains used in (B) were subjected to ChIP assays using anti-PA-tag antibody. (E) Extracts prepared from yeast cells used in (D) were immunoblotted with anti-PA-tag or anti-α-tubulin antibody (loading control). (F) ChIP analysis of DNA binding of Fap1. Strains expressing C-terminally FLAG (3×)-tagged Fap1 were subjected to ChIP assays using anti-FLAG-tag antibody. The same experiment was conducted for strains expressing C-terminally FLAG (3×)-tagged Rap1 as a control (*FAP1-FLAG*: YKK792; *FAP1-FLAG fpr1*Δ: YKK794; *RAP1-FLAG*: YKK1074; *RAP1-FLAG fpr1*Δ: YKK1072). Asterisks: DNA-binding values of Fap1 are less than 0.1 (IP/input) when calculated using the same method as for Rap1. (G) Extracts prepared from yeast cells used in (F) were immunoblotted with anti-FLAG-tag or anti-α-tubulin antibody as in (E).

FKBP12 binds to rapamycin, and this protein-drug complex inhibits the protein kinase activity of TORC1 and thereby inhibits the cell cycle and cell growth in various eukaryotes. Therefore, deletion or certain mutations of *FPR1* confer rapamycin resistance to yeast cells [5, 44]. By contrast, the PPIase activity of FKBP12/Fpr1 is not necessary for rapamycin binding and TORC1 inhibition [45, 46]. To test whether the distinct Fpr1 functions—TORC1 inhibition, PPIase activity, and growth-supporting activity when expressed in *hmo1*Δ*fpr1*Δ cells—are independent or linked, we examined the effect of specific mutations in Fpr1 on these functions. First, *Δfpr1* cells were transformed with plasmids containing WT or a few mutant *fpr1* alleles to assess rapamycin sensitivity. The F43Y mutation in Fpr1 (Fpr1^F43Y^, corresponding to F37Y in human FKBP12) reduces PPIase activity by nearly 1000-fold, but does not affect rapamycin binding and TORC1 inhibition [45, 47], whereas the Y89D mutant of Fpr1 (Fpr1^Y89D^) cannot bind to rapamycin and therefore is deficient in TORC1 inhibition [44]. The amino acids responsible for PPIase activity and/or rapamycin binding, including the aforementioned residues, are highly conserved among FKBP12 family proteins from yeasts to humans (Fig 5A) [48, 49]. Spot assays of the Fpr1 mutants conducted to test for rapamycin sensitivity verified the phenotypes of these mutants (Fig 5B). Next, *hmo1*Δ*fpr1*Δ cells were transformed with the same plasmids containing WT or mutant *fpr1* alleles, and their growth was measured (Fig 5C). The results showed that the expression of WT Fpr1 or Fpr1^F43Y^ enhanced *hmo1*Δ*fpr1*Δ cell growth, whereas that of Fpr1^Y89D^ did not (Fig 5C). Thus, the rapamycin-binding activity of Fpr1, but not its PPIase activity, is required for the Fpr1 function in supporting cellular growth, potentially by promoting the transcription of RPGs. Consistent herewith, ChIP assay results showed that Fpr1^Y89D^ did not bind to the *RPL25* promoter (Fig 5D), although it was expressed at nearly the same level as WT Fpr1 (Fig 5E).

X-ray structural analyses have revealed that a hydrophobic pocket composed of Y83 (Y89 in *S*. *cerevisiae*) and other aromatic amino acid residues serves as a rapamycin-binding site in mammalian FKBP12 [48, 49]. Fpr1^Y89D^ could not bind to DNA, which suggests that certain proteins that bind to this region recruit Fpr1 to RPG promoters. Fap1 was identified as a protein that confers rapamycin resistance to yeast cells by competing with rapamycin for interaction with Fpr1 [50]. Although its physiological function is unknown, Fap1 harbours a putative DNA-binding motif and accumulates in the nuclei of yeast cells upon rapamycin treatment. Thus, we examined Fap1 binding to RPG promoters by ChIP assays using yeast cells expressing C-terminally FLAG (3×)-tagged Fap1. In contrast to Rap1, which binds strongly to the *RPL25* promoter, Fap1 did not bind to this promoter (Fig 5F), although it was expressed at lower levels than Rap1 (Fig 5G). Therefore, Fap1 is unlikely to be involved in Fpr1 binding to RPG promoters.

**Fig 6:**
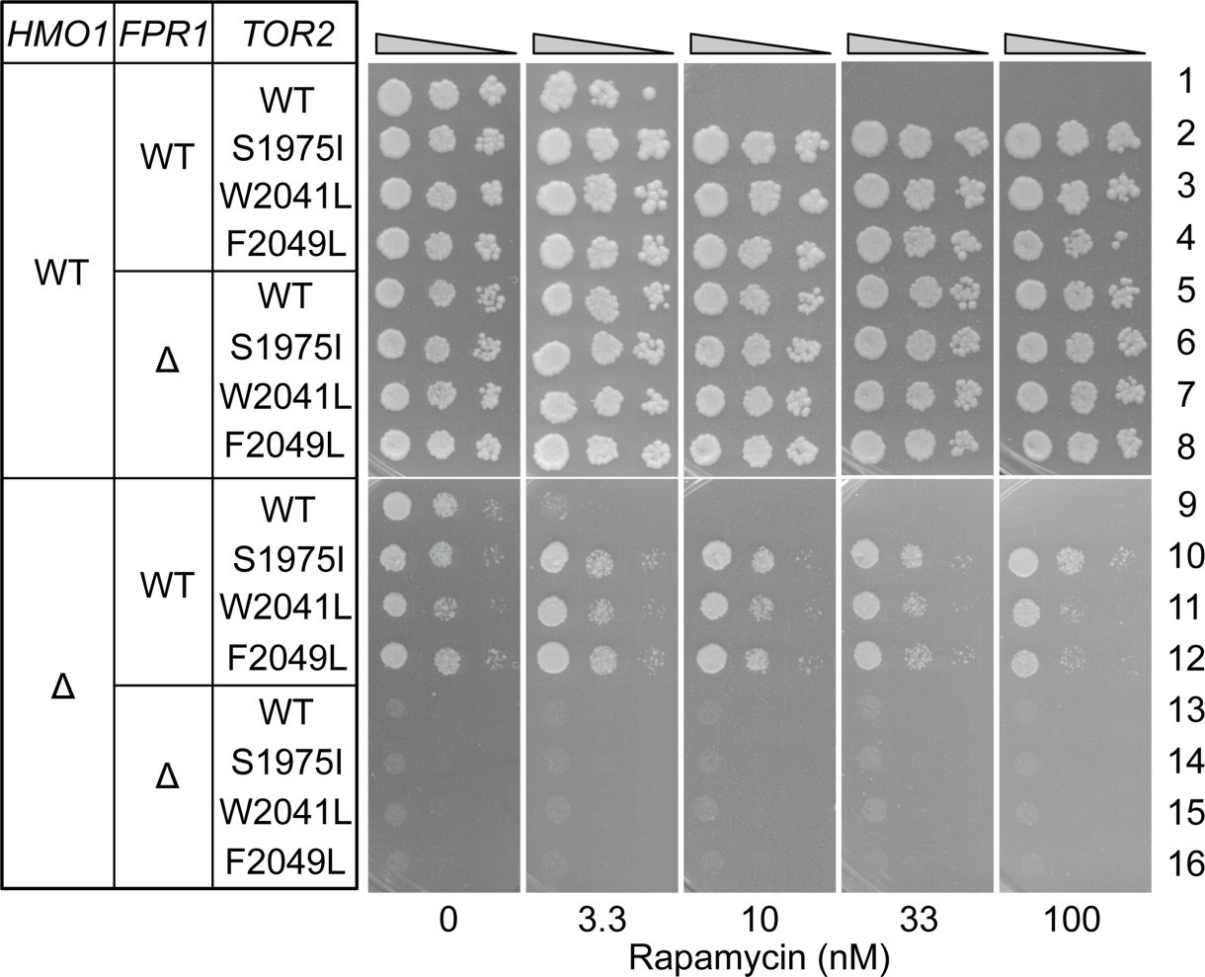
Effect of *tor2* mutations on the function of Fpr1 on RPG promoters. Effect of *tor2* mutations on the growth of WT, *fpr1*Δ, *hmo1*Δ, and *hmo1*Δ*fpr1*Δ cells was examined using spot assays. Plasmids containing *tor2-S1975I* (pKM802), *tor2-W2041L* (pKM804), and *tor2-F2049L* (pKM806) mutant genes or WT *TOR2* (pKM782) were introduced into *tor1*Δ*tor2*Δ [YKK1081], *tor1*Δ*tor2*Δ*fpr1*Δ [YKK1080], *tor1*Δ*tor2*Δ*hmo1*Δ [YKK1093], or *tor1*Δ*tor2*Δ*hmo1*Δ*fpr1*Δ [YKK1097] cells. Subsequently, a plasmid containing WT *TOR2* and *URA3* (pKM731), which was pre-introduced into the host cells, was depleted through plasmid shuffling (using SD plates containing 5-fluoroorotic acid). The resulting strains were spotted onto SD plates containing or not containing rapamycin as indicated, and grown at 30°C for 3 days.

The rapamycin-binding domain of Fpr1 was found to be required for DNA binding and for the Fpr1 function in supporting cell growth, but it remained unclear whether Fpr1 performs these functions by interacting with TORC1. To address this question, we examined the effect of TORC1 mutations that compromise TORC1 interaction with Fpr1 [45]. Specifically, we introduced S1975I, W2041L, or F2049L mutation into Tor2, a catalytic subunit of TORC1, and the proteins were expressed in *tor1*Δ*tor2*Δ cells (Fig 6) (W2041L and F2049L were described respectively as W2042L and F2048L in the original paper [45]). Tor1, which is functionally redundant with Tor2 with respect to RPG transcription, was deleted to precisely evaluate the effect of *tor2* mutations. If Fpr1 requires interaction with TORC1 to promote RPG transcription, these *tor2* mutations would be expected to cause as severe growth defects in *hmo1*Δ cells as in *hmo1*Δ*fpr1*Δ cells. The strains expressing Tor2-S1975I, W2041L, or F2049L grew well, even in the presence of rapamycin, which suggested that these Tor2 mutants specifically lost the interaction with Fpr1, without a marked effect on the essential function of Tor2 (Fig 6, compare strain 1 with strains 2, 3, and 4, and [45]). Notably, *hmo1*Δ cells grew considerably faster than *hmo1*Δ*fpr1*Δ cells in the background of *tor2-S1975I*, *W2041L*, or *F2049L* mutation (Fig 6, compare strains 10, 11, and 12 with strains 14, 15, and 16, respectively). These results strongly suggested that Fpr1 promotes RPG transcription independently of its interaction with TORC1.

Lastly, we examined whether the Fpr1 function of regulating RPG transcription can be reproduced by orthologues from other species. We expressed FKBP1A (human), FKBP1B (human), or Fkh1 (fission yeast) from the *FPR1* promoter in *fpr1*Δ or *hmo1*Δ*fpr1*Δ cells and tested their activities. Fkh1, FKBP1A, and FKBP1B conferred rapamycin sensitivity to *fpr1*Δ cells at a similar level as did WT Fpr1 (Fig 5B), which suggested that these orthologues form a complex with rapamycin to inhibit yeast TORC1. However, the orthologues, unlike WT Fpr1 or Fpr1^F43Y^, did not enhance *hmo1*Δ*fpr1*Δ cell growth (Fig 5C), and ChIP assay results showed that these orthologues did not bind to the *RPL25* promoter (Fig 5D). These findings indicated that the two Fpr1 functions, rapamycin-dependent TORC1 inhibition and regulation of RPG transcription, are mutually independent.

### The N-terminal domain of Fpr1 is essential for its function on RPG promoters

**Fig 7:**
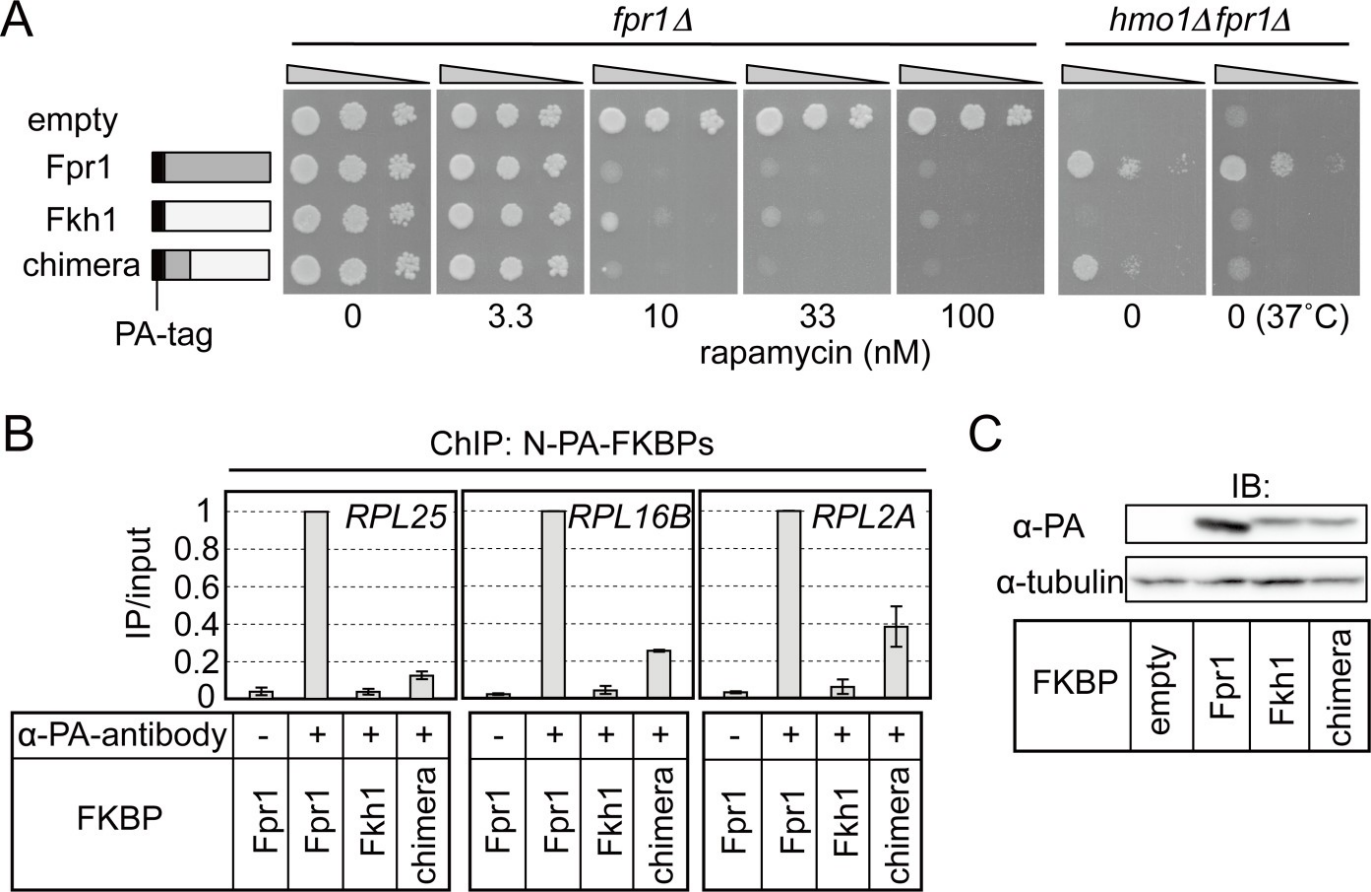
The Fpr1 N-terminal domain is critical for Fpr1 function as a transcriptional regulator. (A) Fpr1, Fkh1, and a mutant Fkh1 protein in which the N-terminal domain (aa 1–25) was replaced with its counterpart from Fpr1 (aa 1–31), were expressed in *fpr1*Δ cells. These proteins were N-terminally fused with PA-tag and expressed from the *FPR1* promoter (Fpr1: pKM738, Fkh1: pKM774, and chimera: pKM795). The resulting strains were spotted onto SD plates containing or not containing rapamycin as indicated, and grown at 30°C for 3 days. The same proteins were also expressed in *hmo1*Δ*fpr1*Δ cells, and the resulting strains were spotted onto SD plates lacking rapamycin and grown at 30°C or 37°C for 3 days. (B) Binding of FKBP proteins tested in (A**)** to specific Fpr1-target promoters (*RPL25*, *RPL16B*, and *RPL2A*) was examined by ChIP assays using anti-PA-tag antibody (as described in Fig 5D). (C) Expression of FKBP proteins used in (A) was examined through immunoblotting (as described in Fig 5E).

To determine which region of Fpr1 is necessary for its specific function of regulating RPG transcription, we expressed several Fpr1–Fkh1 chimeric proteins in cells and assayed their activities. A mutant protein in which the N-terminal domain (1–25 aa) of Fkh1 was replaced with the Fpr1 N-terminal domain (1–31 aa) enhanced *hmo1*Δ*fpr1*Δ cell growth and inhibited TORC1 activity in the presence of rapamycin (Fig 7A). As indicated in Fig 5A, Fpr1 and Fkh1 show lower sequence similarity in the N-terminal region than in other regions, which suggests that the Fpr1 N-terminal domain might carry a specific function. In agreement with the effects on cell growth (Fig 7A), ChIP assay results showed that this N-terminal chimeric protein bound to the promoters of RPGs (*RPL25*, *RPL16B*, and *RPL2A*), albeit more weakly than Fpr1 (Fig 7B). Fkh1 and the N-terminal chimeric protein were expressed at lower levels than Fpr1 (Fig 7C), and thus, the weaker promoter binding might be due to the limited expression of these proteins. The chimeric protein appeared to be unstable when expressed in yeast cells, because its suppressive effect on *hmo1*Δ*fpr1*Δ cell growth was temperature-sensitive (Fig 7A, 37°C). Given that Fpr1^Y89D^, which harboured an intact N-terminal domain, did not bind to the RPG promoter (Fig 5D), the N-terminal domain and rapamycin-binding domain of Fpr1 likely function cooperatively to support promoter binding and transcriptional regulation of Fpr1-target genes.

## Discussion

In examining how Hmo1 and Fpr1 affect the transcription of RPGs, we obtained several key results: (1) Fpr1 specifically binds to nearly all RPG promoters and to a few non-RPG promoters; (2) Fpr1 binding to RPG promoters depends on Rap1 and Hmo1 entirely or partially, respectively (at least on the *RPL25* promoter), although no significant correlation was observed between the binding of Hmo1 and Fpr1 with regard to the amount; (3) Fpr1 promotes Fhl1/Ifh1 binding to a subset of RPG promoters; and (4) transcriptional regulation by Fpr1 is a physiological process unrelated to the non-physiological role of FKBP12 inhibition of TORC1. On the basis of these findings, we conclude that Fpr1 functions as a transcriptional regulator of RPGs.

A previous study demonstrated that the defective growth of *hmo1*Δ*fpr1*Δ cells was restored by increasing *RPL25* expression [26]. Our RNA-seq results showed that mRNA expression of *RPL25* was more strongly downregulated than that of all other essential genes in *hmo1*Δ*fpr1*Δ cells when compared to the expression in WT cells, suggesting that the synthetic growth defect of *hmo1*Δ*fpr1*Δ cells is due to the lack of Rpl25 protein, for which no paralogues exist and whose encoding gene is recognised to be a haplo-insufficient gene in diploid cells (https://www.yeastgenome.org/). On the other hand, our preliminary RNA-seq analysis showed that increasing *RPL25* expression did not restore the expression of other RPGs in *hmo1*Δ*fpr1*Δ cells (that will be published elsewhere), suggesting that reduced expression of RPGs in *hmo1*Δ*fpr1*Δ cells was a direct effect of Hmo1 and Fpr1 depletion, rather than an indirect effect of severe growth retardation induced by the lack of Rpl25.

The ChIP signal of Fpr1 is considerably weaker than that of Rap1 and Hmo1 (compare Fig 1A [Fpr1-FLAG], Fig 1D [PA-Fpr1], Fig 2A [Hmo1-FLAG], and Fig 5F [Rap1-FLAG]; binding of N-terminally PA-tagged Fpr1 was more efficiently detected than that of C-terminally FLAG-tagged Fpr1, but it was considerably weaker than that of Hmo1 and Rap1). Such weak ChIP signal of Fpr1, together with the apparent lack of the characteristic DNA-binding motif in Fpr1, suggests that Fpr1 indirectly binds to DNA via interaction with other DNA-binding proteins. Fpr1 and Rap1 appear to bind to overlapping sites in nearly all RPG promoters, and Fpr1 binding was lost when the Rap1-binding site was deleted, at least in the *RPL25* promoter. Thus, Rap1 or certain Rap1-associating factor(s) might recruit Fpr1 to RPG promoters potentially through interaction with the N-terminal domain and/or rapamycin-binding domain of Fpr1.

Previous genome-wide ChIP analyses have identified Rap1 interaction with several target genes other than RPGs [28, 30, 32, 36]. Rap1 can bind to telomere repeats and glycolytic enzyme gene promoter regions, and Rap1 has been suggested to alter its conformation on different target genes and thereby recruit distinct transcription factors [51, 52]. Rap1 interacts with factors such as Rif1 and Sir on telomere repeats to maintain telomere length and inhibit transcription [53–55], whereas on glycolytic enzyme gene promoter regions, it recruits Gcr1, an activator of these genes [56, 57]. Notably, although Fpr1 binds to RPG promoters in a Rap1-dependent manner, it does not bind to Rap1-target genes other than RPGs, with very few exceptions, which suggests that Fpr1 functions as a coactivator dedicated to RPG transcription (Fig 1C and S2 Fig). It is possible that Fpr1 recognizes a conformation of Rap1 that is specifically induced upon binding to RPG promoters, although we did not detect a direct interaction between Rap1 and Fpr1 *in vitro* (Ni-pulldown assay, using recombinant Rap1 and Fpr1 proteins) or *in vivo* (two-hybrid assay).

Fhl1/Ifh1 are recruited to Hmo1-enriched RPG promoters in a manner requiring Hmo1 function, on the other hand, they are recruited to Hmo1-limited RPG promoters in a manner independent of Hmo1. However, it has been unclear how Fhl1/Ifh1 are recruited to Hmo1-limited RPG promoters. In this study, we showed that Fhl1/Ifh1 are recruited to a subset of Hmo1-limited RPG promoters in a manner requiring Fpr1 function. Based on our ChIP-seq analyses, we classified all RPGs into several groups and categories (S3 Fig). As indicated by this classification, Fhl1 binding to 52 RPGs (37.7% of 138 RPGs) was influenced by deletion of *HMO1*, but not by that of *FPR1*, the binding to 24 RPGs (17.4%) was influenced by deletion of *FPR1*, but not by that of *HMO1*, and the binding to 15 RPGs (10.9%) was influenced by deletion of both *HMO1* and *FPR1*. Currently, the mechanisms by which Hmo1 and Fpr1 promote Fhl1/Ifh1 binding to their target promoters remain unclear, but our finding that the proteins could target apparently distinct sets of RPG promoters (e.g., groups **b** and **c** in S3 Fig) suggests the existence of at least two different pathways for Fhl1/Ifh1 recruitment to the RPG promoters that are facilitated primarily by Hmo1 or by Fpr1, respectively. This agrees with the finding that deletion of both *HMO1* and *FPR1* more strongly compromised cell growth than deletion of each single gene. Furthermore, *FPR1* deletion exerted only a limited effect on the binding of Hmo1 to distinct types of RPGs (Fig 2C), which suggests that it does not affect Fhl1 binding to those RPGs through a reduction in Hmo1 binding, and vice versa for the effect of *HMO1* deletion on Fpr1 binding. On the other hand, it should be noted that Fhl1 binding to ~29% of the RPGs (group **a**) was not or only weakly influenced by deletion of *HMO1* and/or *FPR1*. These promoters are largely deficient in Hmo1 binding, and their transcription was also only slightly affected by deletion of *HMO1* and/or *FPR1* (S9 Fig). These results suggest the existence of another pathway for recruitment of Fhl1 to DNA that is independent of Hmo1 and Fpr1. In a previous study, Knight et al. proposed that a subset of RPG promoters showing a limited amount of Hmo1 binding, such as *RPL11A*, *RPL28*, *RPS22B*, and *RPL8A*, should be recognized directly by Fhl1. This was supported by the observation that Fhl1 binding to these promoters was reduced significantly by mutation of the Fhl1-binding motif in these promoters or by deletion of the DNA-binding domain (FHD) of Fhl1 [28]. Importantly, the *RPL11A*, *RPL28*, and *RPL8A* promoters were categorized by our classification into group **a**, where Fhl1 binding appeared to be independent of Hmo1 and Fpr1 (S3 Fig). Furthermore, the *RPS25A* and *RPL24B* promoters, which were shown to be bound by Fhl1 in a manner independently of FHD and the Fhl1-binding motif [28], were categorized by our classification into group **b** and **c**, where Fhl1 binding could be influenced significantly by deletion of *FPR1* and *HMO1*, respectively (S3 Fig). These observations suggest that the third pathway for recruitment of Fhl1 to DNA described above could be implemented by direct binding of Fhl1 via its FHD, and that other RPGs belonging to group **a** might also employ such mechanism to recruit Fhl1 to their promoters.

It was clearly shown that Rap1 plays a crucial role in recruiting Fhl1/Ifh1 to RPG promoters of both category I (Hmo1-enriched) and category II (Hmo1-limited) [28]. However, it still remains unclear how Rap1 recruits Fhl1/Ifh1 to the RPG promoters. ChIP-seq results demonstrated that the target genes of Fpr1 and Fhl1 largely overlapped (Fig 3C), and Rap1, Fpr1, and Fhl1 colocalized at the same set of RPG promoters (S4 Table). Considering these results together with the expected positions of these factors on their target promoters, we propose that Fpr1 functions in concert with Rap1 to recruit Fhl1/Ifh1 to a subset of its target promoters.

Considering that the coordinated synthesis of ribosomal components is critical for the efficient production of intact ribosomes, it is unclear why distinct Fhl1/Ifh1-recruitment mechanisms involving Hmo1 and/or Fpr1 exist in yeast. Although Fpr1 binds to more (nearly all) RPG promoters than does Hmo1, *FPR1* deletion only weakly affected the transcription of most RPGs and yeast growth as compared to *HMO1* deletion, as if Fpr1-mediated pathway is subsidiary to the Hmo1- mediated pathway. Possibly, the Fpr1-mediated pathway may play a more crucial role in the regulation of RPG expression in response to certain specific nutrient conditions. Notably, Fpr1 binds to the promoter of *GCN4* (Fig 1B and Fig 1D), which encodes a transcription factor that promotes amino acid synthesis in response to amino acid starvation. To examine whether Fpr1 is involved in the amino acid starvation response, we tested whether *fpr1*Δ, *hmo1*Δ, and *hmo1*Δ*fpr1*Δ cells would show altered sensitivity to sulfometuron methyl, an inhibitor of amino acid synthesis. The results indicated that deletion of *FPR1* and/or *HMO1* did not cause any significant sensitivity to the inhibitor, while the deletion of *GCN4* did. These findings suggested that it is unlikely that Fpr1 and Hmo1 are involved in the regulation of amino acid synthesis. Thus, it is currently unknown why Fpr1 binds to the *GCN4* promoter.

The physiological functions of FKBP12 orthologues appear to be diverse among eukaryotes, and only the inhibitory effects of FKBP12 on TORC1 and calcineurin in the presence of immunosuppressive drugs are widely conserved. For more than 20 years since the elucidation of the molecular mechanisms by which the FKBP12/rapamycin complex inhibits TORC1 to downregulate RPG transcription, it has remained unclear how FKBP12 is involved in ribosomal production under physiological conditions in the absence of rapamycin. The complementation analysis performed in this study using FKBP12 orthologues from other species indicated that two activities of FKBP12, rapamycin-dependent TORC1 inhibition and binding to RPG promoters, are mutually independent. Furthermore, Fpr1 supported the growth of *hmo1*Δ*fpr1*Δ cells expressing mutant Tor proteins that do not interact with Fpr1. These results strongly suggest that Fpr1 participates in RPG transcription under physiological conditions independently of its interaction with TORC1. This is another critical finding of this study. Further mutation analysis of Fpr1 and domain-swapping analysis between Fpr1 and Fkh1 revealed that the N-terminal and rapamycin-binding domains of Fpr1 are crucial for promoter binding of Fpr1; however, it remains unclear whether the mutations affect the DNA-binding activity of Fpr1, or its nuclear localization. Moreover, the Fpr1 region/mechanism involved in the recruitment of Fhl1/Ifh1 remains unidentified.

Fpr1 mutation analysis results here and in previous studies suggest that the PPIase activity of Fpr1 is not necessary for RPG transcription and drug-dependent TORC1 inhibition [26, 45, 60]. Although the functional importance of PPIase activity has been demonstrated, the enzyme’s substrates remain unknown. A previous random-screening study of Fpr1 mutants lacking rapamycin sensitivity indicated that most of the mutant proteins were not expressed stably [44]. Considering that FKBP12 family proteins are very small, even a point mutation could affect the entire protein structure. In this regard, our results obtained using FKBP12 orthologues from other species or mutant Tor proteins are likely physiologically relevant, because the FKBP12 proteins tested in these experiments were WT proteins and appear to be expressed stably. To more precisely clarify how Fpr1 performs its function in RPG transcription, such as by recruiting Fhl1/Ifh1 to the promoter, it will be crucial to isolate *fpr1* mutants that affect only a specific function of Fpr1 and do not destabilise the protein itself. For this purpose, the TORC1-inhibitory activity of the FKBP12 mutants could be used as a hallmark of the stability of the entire protein structure.

In contrast to the good correlation between the levels of Fhl1 binding to the Hmo1-enriched promoters and those of their transcription upon deletion of *HMO1*, there was only a weak correlation between the levels of Fhl1 binding to the RPG promoters belonging to category **b** and those of their transcription or cell growth upon deletion of *FPR1* (S9 Fig). This result indicated that Fhl1 binding, as measured by ChIP, is not linearly related to transcriptional output, especially in the *fpr1Δ* strain. As a possible explanation for such weak correlation, we suspect that a minimum residence of Fhl1 on the RPG promoters could assure sufficient transcription of those promoters. Alternatively, the ChIP signal of Fhl1 may not fully reflect the extent of its binding to RPG promoters due to changes in some binding property of Fhl1. For example, certain conformational changes of Fhl1 may occur only when Fpr1 is absent from the promoter, masking the epitope used in our ChIP assays. To test such a possibility, another method to measure Fhl1/Ifh1 binding to DNA independently of immunoprecipitation, e.g., chromatin endogenous cleavage (ChEC) [58] or its combination with high-throughput sequencing (ChEC-Seq) [59], should be effective.

In this study, we evaluated the effects of *fpr1*Δ and/or *hmo1*Δ on transcription using RNA-seq, which quantifies steady-state, not nascent mRNA levels. Although there was a good correlation between the RNA-seq results and ChIP assay results for Sua7/TFIIB, a PIC component, at least for the RPG promoters tested here (e.g. *RPS5*, *RPS25A*, and *RPL25* in Fig 4C), the effects of *fpr1*Δ and/or *hmo1*Δ on transcription might be obscured by a transcript buffering effect [61]. Therefore, further experiments to detect on-going transcription, such as ChIP-seq for RNA polymerase II occupancy or comparative dynamic transcriptome analysis [62], will be required to clarify the function of Fpr1 on its target promoters.

## Methods

### Yeast strains and plasmids

The yeast strains and oligonucleotides used in this study are listed in S1 Table and S2 Table, respectively. Plasmids containing *S*. *pombe fkh1*^*+*^ and *Oryza sativa TIR1* were provided by the National Bio-Resource Project (NBRP), Japan. Detailed information regarding each strain and the protocol used for plasmid construction are provided in the S1 text. The yeast culture conditions for each experiment are described in the figure legends.

### ChIP analysis

ChIP analysis was conducted as described [33]. Briefly, DNA was fragmented by sonication to an average size of 400–500 bp for standard ChIP or of 100–200 bp for high-resolution ChIP. Immunoprecipitation was conducted using Dynabeads Protein G (Invitrogen, Carlsbad, CA, USA), anti-FLAG-tag antibody (Sigma-Aldrich, St. Louis, MO, USA; M2), anti-PA-tag antibody (FujiFilm Wako Pure Chemical Corporation, Osaka, Japan; NZ-1), and polyclonal antibody against Rap1 (Santa Cruz Biotechnology, Dallas, TX, USA; yC-19) or Hmo1 [29]. Real-time quantitative PCR analyses were performed using a KAPA SYBR Fast qPCR kit (Kapa Biosystems, Wilmington, MA, USA). Each experiment was performed in triplicate, and the mean and standard deviation of the ratio of immunoprecipitated DNA to input DNA (IP/input) were calculated. Primer pairs used for quantitative PCR are described in the S1 text.

### ChIP-seq analysis

ChIP-seq analysis for Fpr1 was performed using a yeast strain expressing N-terminally PA-tagged Fpr1. Strain YKK207 harbouring plasmid pKM740 was grown in yeast extract-peptone-dextrose medium containing aureobasidin A (0.2 μg/mL) at 30°C, to mid-log phase. ChIP DNA was prepared using the methods used for ChIP analysis and the anti-PA tag antibody. Sequencing libraries were prepared using the NEBNext ChIP-Seq Library Prep Master Mix Set for Illumina (New England Biolabs, Ipswich, MA, USA), following the manufacturer’s protocols, in which 15 PCR cycles were used to amplify the ChIP DNA and input DNA. Pooled libraries were sequenced on an Illumina HiSeq 2500 instrument, generating 100-bp paired-end reads with 6-bp index tags. ChIP-seq data were analysed using CLC Genomics Workbench ver. 11.0. Sequence reads were mapped to the *S*. *cerevisiae* s288c reference genome (NC_001133–NC_001148). Peaks showing statistically significant enrichment were selected using a 10^−50^ *p*-value cut-off and an enrichment ratio of >10. To calculate the enrichment ratio of detected peaks, we exported the per-base read coverage into CLC Genomics Workbench and then computed the normalised read coverage by dividing the values by the total number of mapped reads in each sample. We calculated the per-base enrichment ratio by dividing the normalised read coverage by the normalised read coverage of a matched input DNA control sample. The mean 5-bp enrichment ratio was then calculated. These calculations were performed using BedTools [https://www.ncbi.nlm.nih.gov/pubmed/25199790] [63] within the Galaxy server [https://www.ncbi.nlm.nih.gov/pubmed/16169926]. We also visually inspected the per-base enrichment ratio, using IGV [https://www.ncbi.nlm.nih.gov/pubmed/22517427]. Read data for ChIP-seq experiments for Fpr1 (IP and input) are accessible at the DDBJ Sequence Read Archive (DRA) (https://ddbj.nig.ac.jp/DRASearch/) under accession no. DRA007139. ChIP-seq analyses for Fhl1 were conducted using almost identical methods and yeast strains expressing C-terminally FLAG-tagged Fhl1 in the background of WT (YKK1090), *fpr1*Δ (YKK1087), *hmo1*Δ (YKK1091), and *hmo1*Δ*fpr1*Δ (YKK1086). Peaks showing statistically significant enrichment were selected using a 10^−50^ *p*-value cut-off and an enrichment ratio of >5. Read data for ChIP-seq experiments for Fhl1 (IP and input) are accessible DRA (https://ddbj.nig.ac.jp/DRASearch) under accession no. DRA008879.

### Protein depletion

Protein depletion using the AID degron system was conducted according to a previously reported protocol [40]. Yeast strains were grown to mid-log phase in SD medium at 30°C, and were then treated with indole-3-acetic acid (0.5 mM) for 60 min. Then, the yeast cells were collected and subjected to immunoblot analysis or ChIP assay.

### RNA-seq analysis

RNA levels of all genes in WT (YKK461 and YKK468), *hmo1*Δ (YKK459 and YKK464), *fpr1*Δ (YKK455 and YKK463), and *hmo1*Δ*fpr1*Δ (YKK458 and YKK469) cells were examined using RNA-seq analysis. Total RNA for RNA-seq analysis was prepared using ISOGEN with Spin Column (Nippon Gene, Tokyo, Japan). cDNA sequencing libraries were prepared from 500 ng of total RNA by using a TruSeq RNA Library Prep Kit v2 (Illumina) according to the manufacturer’s protocols. The libraries were amplified using 15 PCR cycles. Pooled libraries were sequenced on an Illumina HiSeq 2500 instrument, generating 100-bp paired-end reads with 6-bp index tags. Sequencing data were analysed using CLC Genomics Workbench ver. 7.0.4. Read-adapter and quality-trimmed reads were mapped to the *S*. *cerevisiae* s288c reference genome (NC_001133–NC_001148) using default parameters. Read data for RNA-seq experiments are accessible at DRA (https://ddbj.nig.ac.jp/DRASearch) under accession no. DRA008879.

### Immunoblot analysis

Yeast cell extract was prepared according to a published protocol [64]. Proteins were detected using an anti-FLAG tag antibody, anti-PA-tag antibody, anti-α-tubulin antibody (Santa Cruz Biotechnology, Dallas, TX, USA; YOL1/34), or anti-Fpr1-antibody. Polyclonal antibody directed against Fpr1 (aa 1–114) was raised in rabbits using gel-purified C-terminally His-tagged Fpr1 expressed in *E*.*coli*.

## Supporting information

S1 Fig Fpr1 binds specifically to the RPG promoters. A ChIP assay was conducted to examine whether Fpr1 binds specifically to the RPG promoters *in vivo*. Yeast strains expressing N-terminally PA-tagged Fpr1 (pKM738) or untagged Fpr1 (no tag control; pKM304) were subjected to a ChIP assay using anti-PA-tag antibody. Values obtained in ChIP assays are expressed as ratios relative to that for PA-tagged Fpr1.
(PDF)

S2 Fig Genome-wide identification of binding loci for Fpr1 and Fhl1. Binding positions of Fpr1 and Fhl1 (in WT, *fpr1*Δ, *hmo1*Δ, and *hmo1*Δ*fpr1*Δ cells), identified by ChIP-seq, are summarised for each chromosome. Names of genes harbouring Fpr1-binding sites are shown in the top panel, and serial numbers are assigned within each chromosome. Fhl1-binding positions in WT, *fpr1*Δ, *hmo1*Δ, and *hmo1*Δ*fpr1*Δ cells are shown in the second to fifth panels, as described on the left. The colours (black, red, blue, or green) of Fhl1-binding peaks/loci indicate the influence of deletion of *HMO1* and/or *FPR1* on Fhl1 binding to those loci. Superscripts of peak numbers correspond to the category numbers assigned to the peaks, as described in S3 Fig.
(PDF)

S3 Fig Influence of deletion of *HMO1* and/or *FPR1* on Fhl1 binding to its target loci. Amounts of Fhl1 binding to its target loci, which were measured by ChIP-seq, are presented as bar graphs. Bars featuring four colours, blue, red, yellow, and green, represent Fhl1 binding to the genes in each panel in WT, *fpr1*Δ, *hmo1*Δ, and *hmo1*Δ*fpr1*Δ cells, respectively. Genes within red or blue boxes were also examined in individual ChIP assays, shown in Fig 3B or S4 Fig, respectively. ChIP-assay values are expressed as ratios relative to values obtained for WT cells. Fpr1-target genes harbouring Fhl1-binding sites are classified into four groups according to the influence of deletion of *HMO1* and/or *FPR1* on Fhl1 binding: **a**: not influenced by deletion of *HMO1/FPR1* (black symbol); **b**: influenced by deletion of *FPR1*, but not by that of *HMO1* (red symbol); **c**: influenced by deletion of *HMO1*, but not by that of *FPR1* (blue symbol); and **d**: influenced by deletion of *HMO1/FPR1* (green symbol). Coloured symbols at the top of bar graphs reflect this classification. Groups **a**, **b,** and **c** are further divided into two categories. These classifications are summarised in the table at the bottom of this figure and explained in the text.
(PDF)

S4 Fig Effect of deletion of *HMO1* and/or *FPR1* on Fhl1 binding to specific RPG promoters. To examine the influence of deletion of *HMO1* and/or *FPR1* on Fhl1 binding to RPG promoters, ChIP assays were conducted for additional Fpr1-target genes as described in Fig 3B. Coloured symbols at the top of each panel reflect the classification of Fpr1-target genes, as described in S3 Fig.
(PDF)

S5 Fig Effect of rapid depletion of Fpr1 on Fhl1 binding to specific RPG promoters. (A) Rapid depletion analysis of Fpr1 using the AID degron system. Two *fpr1*Δ strains expressing C-terminally FLAG-tagged Fhl1, expressing or not expressing *Oryza sativa TIR1*, were transformed with empty plasmid (indicated as “–”), plasmid expressing untagged-Fpr1 (–AID), or plasmid expressing C-terminally AID-tagged-Fpr1 (+AID). Depletion of Fpr1 was achieved by addition of indole-3-acetic acid (IAA, 0.5 mM) for 60 min, as described in the Methods section. The amount of Fpr1 proteins was measured by immunoblotting using anti-Fpr1 antibody. (B) Fhl1 binding to the promoters of *RPS25A*, *RPS30B*, and *RPS5* was examined by ChIP assays using yeast cells in which Fpr1 was depleted or not, as described in (A).
(PDF)

S6 Fig Binding positions of Fpr1 and Fhl1 on their target RPG promoters. Precise binding sites of Fpr1 and Fhl1 (in WT, *fpr1*Δ, *hmo1*Δ, and *hmo1*Δ*fpr1*Δ cells) at the promoters of *RPL25*, *RPS5*, and *RPS25A*, which were identified by ChIP-seq; IGV_2.4.8 software was used for depicting the sites. Red and blue dashed lines indicate the binding peaks of Fpr1 and Fhl1 in WT cells. The grey rectangles, black arrows, and red asterisks indicate the coding region, transcriptional direction, and position of a start codon, of each RPG, respectively.
(PDF)

S7 Fig Effect of rapamycin on the binding of Fpr1 and Fhl1 to three RPG promoters. Yeast cells expressing both N-terminally PA-tagged Fpr1 and C-terminally FLAG-tagged Fhl1 were treated with rapamycin (+: 80 nM, ++: 400 nM) for 60 min, and subjected to ChIP assay using anti-PA-tag antibody or anti-FLAG-tag antibody.
(PDF)

S8 Fig Effect of deletion of *HMO1* and/or *FPR1* on genome-wide transcription. (A) RNA-seq analyses were conducted to examine the effects of deletion of *HMO1* and/or *FPR1* on genome-wide transcription as described in Fig 4A. The values obtained for RNA levels of genes in the entire genome were expressed as a ratio to the value measured for WT cells, aligned in the descending order of values for *hmo1*Δ*fpr1*Δ cells, and are summarised here as a heatmap. In this heatmap, maximal and minimal values for fold-changes were set at +5 and –5, respectively, and FDR *p*-value of <0.05 was used as the cut-off. (B) RPGs are marked here with green lines in the heatmap from (A).
(PDF)

S9 Fig Effect of deletion of Hmo1 and/or Fpr1 on transcription and Fhl1 binding of Fpr1-target genes. To evaluate the correlation between the effects of deletion of *HMO1* and/or *FPR1* on Fhl1 binding to and transcription of Fpr1-target genes, the heatmap in Fig 4A was modified as follows. The Hmo1-binding results are ChIP-seq data quoted from Reja et al. [30]. Fhl1 binding to each gene in WT was set as 1, and the relative strength of Fhl1 binding to the same gene in other strains (*fpr1*Δ, *hmo1*Δ, and *hmo1*Δ*fpr1*Δ) was calculated as a ratio to that of WT cells. The data were reanalysed by using the hierarchical clustering method according to the binding profiles of Fhl1 and are summarised as a heatmap. Coloured symbols attached to each gene show the influence of deletion of *HMO1*/*FPR1* on Fhl1 binding, as described in S3 Fig.
(PDF)

S1 Table *S*. *cerevisiae* strains used in this study. (XLSX)

S2 Table Oligonucleotides used in this study. (XLSX)

S3 Table Target genes of Fpr1 and Fhl1 revealed using ChIP-seq. (XLSX)

S4 Table Binding of Fpr1, Fhl1, and Rap1 to RPGs. (XLSX)

S5 Table Comparison of transcripts of all genes among WT, *hmo1*Δ, *fpr1*Δ, and *hmo1*Δ*fpr1*Δ cells. (XLSX)

S6 Table Comparison of transcripts of RPGs among WT, *hmo1*Δ, *fpr1*Δ, and *hmo1*Δ*fpr1*Δ cells. (XLSX)

S7 Table ChIP Data for Fig 1A. (XLSX)

S8 Table ChIP Data for Fig 1D. (XLSX)

S9 Table ChIP Data for Fig 2A. (XLSX)

S10 Table ChIP Data for Fig 2B. (XLSX)

S11 Table ChIP Data for Fig 2C. (XLSX)

S12 Table ChIP Data for Fig 3B. (XLSX)

S13 Table ChIP Data for Fig 4C. (XLSX)

S14 Table ChIP Data for Fig 5D. (XLSX)

S15 Table ChIP Data for Fig 5F. (XLSX)

S16 Table ChIP Data for Fig 7B. (XLSX)

S17 Table ChIP Data for S1 Fig. (XLSX)

S18 Table ChIP Data for S4 Fig. (XLSX)

S19 Table ChIP Data for S5B Fig. (XLSX)

S20 Table ChIP Data for S7 Fig. (XLSX)

S1 Text Information about yeast strains and plasmids used in this study, and oligonucleotides used for ChIP analysis. (DOCX)
