# Supplementary material for: Fpr1, a primary target of rapamycin, functions as a transcription factor for ribosomal protein genes cooperatively with Hmo1 in Saccharomyces cerevisiae
Source: PLoS Genet. 2020 Jun 30;16(6):e1008865. doi: 10.1371/journal.pgen.1008865 (PMC7357790; doi:10.1371/journal.pgen.1008865)
Supplement: S1 Fig — A ChIP assay was conducted to examine whether Fpr1 binds specifically to the RPG promoters in vivo. Yeast strains expressing N-terminally PA-tagged Fpr1 (pKM738) or untagged Fpr1 (no tag control; pKM304) were subjected to a ChIP assay using anti-PA-tag antibody. Values obtained in ChIP assays are expressed as ratios relative to that for PA-tagged Fpr1. (PDF) [file pgen.1008865.s001.pdf]

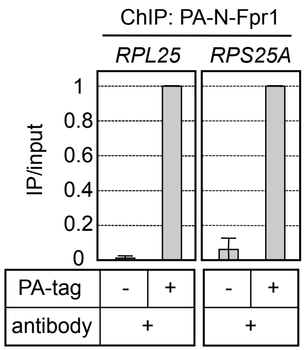

S1 Fig. Fpr1 binds specifically to the RPG promoters.
A ChIP assay was conducted to examine whether Fpr1 binds specifically to the RPG promoters *in vivo*. Yeast strains expressing N-terminally PA-tagged Fpr1 (pKM738) or untagged Fpr1 (no tag control; pKM304) were subjected to a ChIP assay using anti-PA-tag antibody. Values obtained in ChIP assays are expressed as ratios relative to that for PA-tagged Fpr1.
