# Supplementary material for: Fpr1, a primary target of rapamycin, functions as a transcription factor for ribosomal protein genes cooperatively with Hmo1 in Saccharomyces cerevisiae
Source: PLoS Genet. 2020 Jun 30;16(6):e1008865. doi: 10.1371/journal.pgen.1008865 (PMC7357790; doi:10.1371/journal.pgen.1008865)

data range 50

black: Fhl1 binding is not influenced by *hmo1Δ/fpr1Δ*
red: Fhl1 binding is influenced by *fpr1Δ* but not by *hmo1Δ*
blue: Fhl1 binding is influenced by *hmo1Δ* but not by *fpr1Δ*
green: Fhl1 binding is influenced by *hmo1Δ/fpr1Δ*

Chr.1

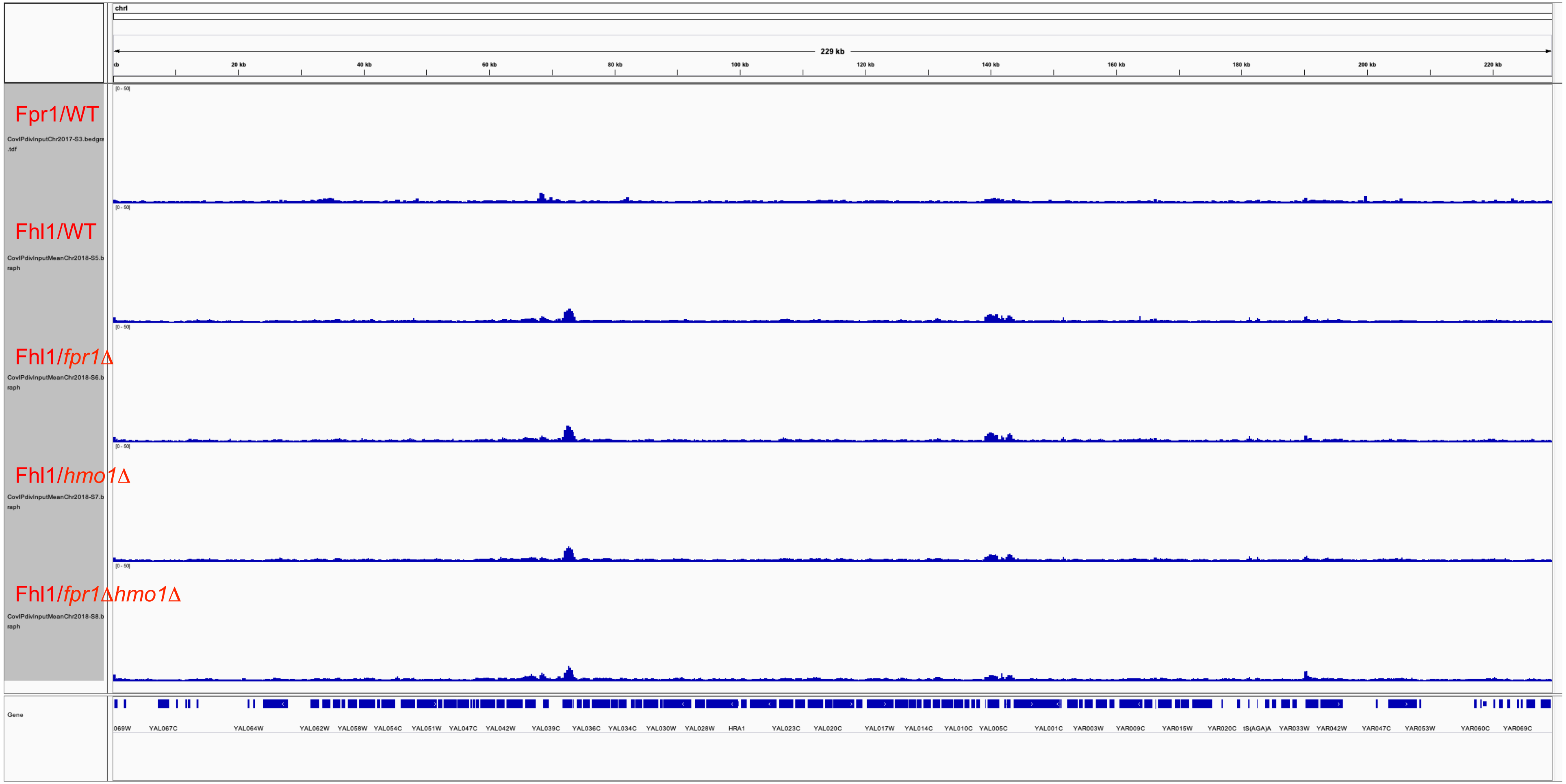


**S2 Fig. Genome-wide identification of binding loci for Fpr1 and Fhl1.**
Binding positions of Fpr1 and Fhl1 (in WT, *fpr1Δ*, *hmo1Δ*, and *hmo1Δfpr1Δ* cells), identified by ChIP-seq, are summarised for each chromosome. Names of genes harbouring Fpr1-binding sites are shown in the top panel, and serial numbers are assigned within each chromosome. Fhl1-binding positions in WT, *fpr1Δ*, *hmo1Δ*, and *hmo1Δfpr1Δ* cells are shown in the second to fifth panels, as described on the left. The colours (black, red, blue, or green) of Fhl1-binding peaks/loci indicate the influence of deletion of *HMO1* and/or *FPR1* on Fhl1 binding to those loci. Superscripts of peak numbers correspond to the category numbers assigned to the peaks, as described in S3 Fig.

data range 50

black: Fhl1 binding is not influenced by *hmo1Δ/fpr1Δ*
red: Fhl1 binding is influenced by *fpr1Δ* but not by *hmo1Δ*
blue: Fhl1 binding is influenced by *hmo1Δ* but not by *fpr1Δ*
green: Fhl1 binding is influenced by *hmo1Δ/fpr1Δ*

Chr.2

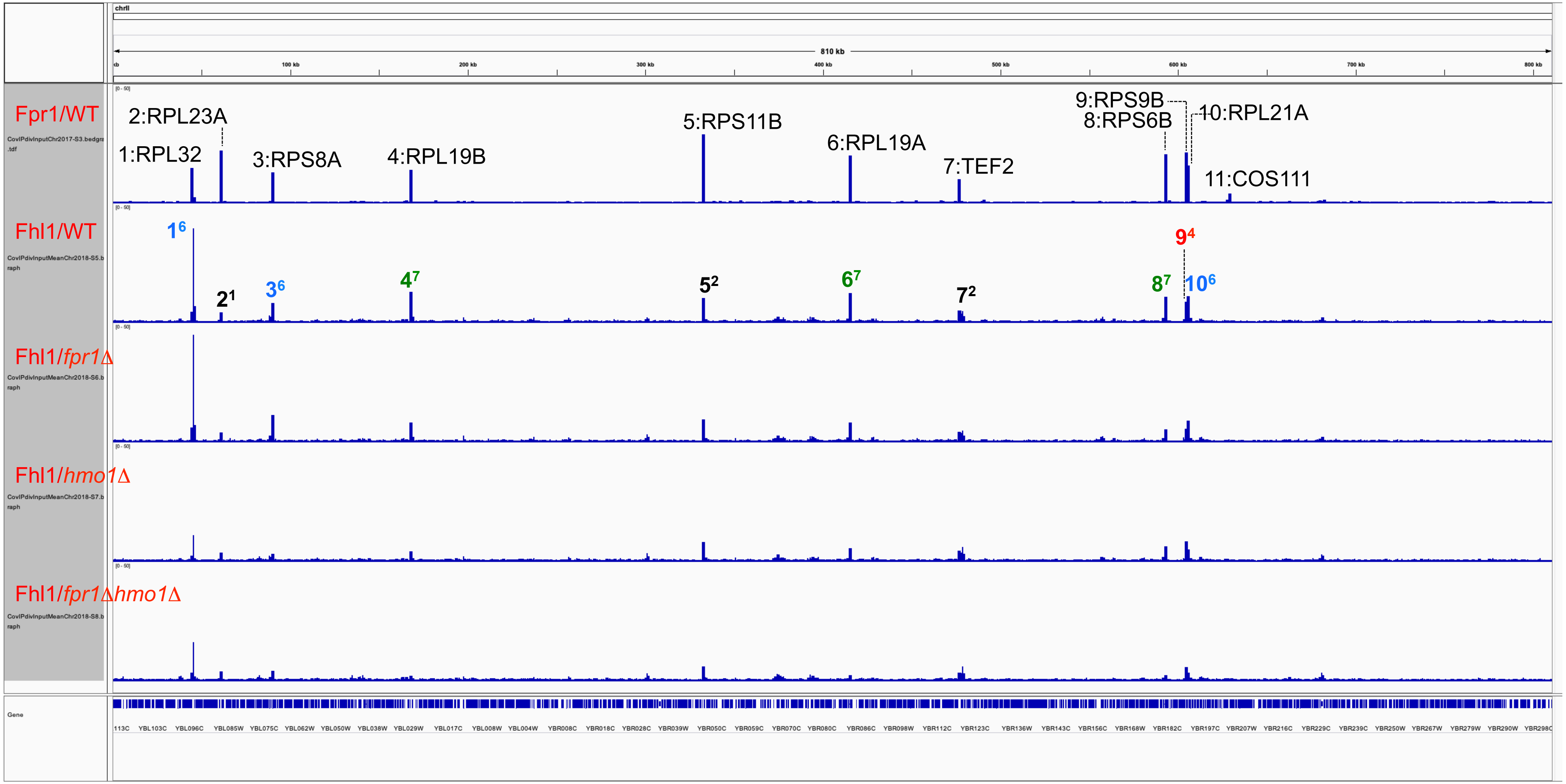

data range 50

black: Fhl1 binding is not influenced by *hmo1Δ/fpr1Δ*
red: Fhl1 binding is influenced by *fpr1Δ* but not by *hmo1Δ*
blue: Fhl1 binding is influenced by *hmo1Δ* but not by *fpr1Δ*
green: Fhl1 binding is influenced by *hmo1Δ/fpr1Δ*

Chr.3

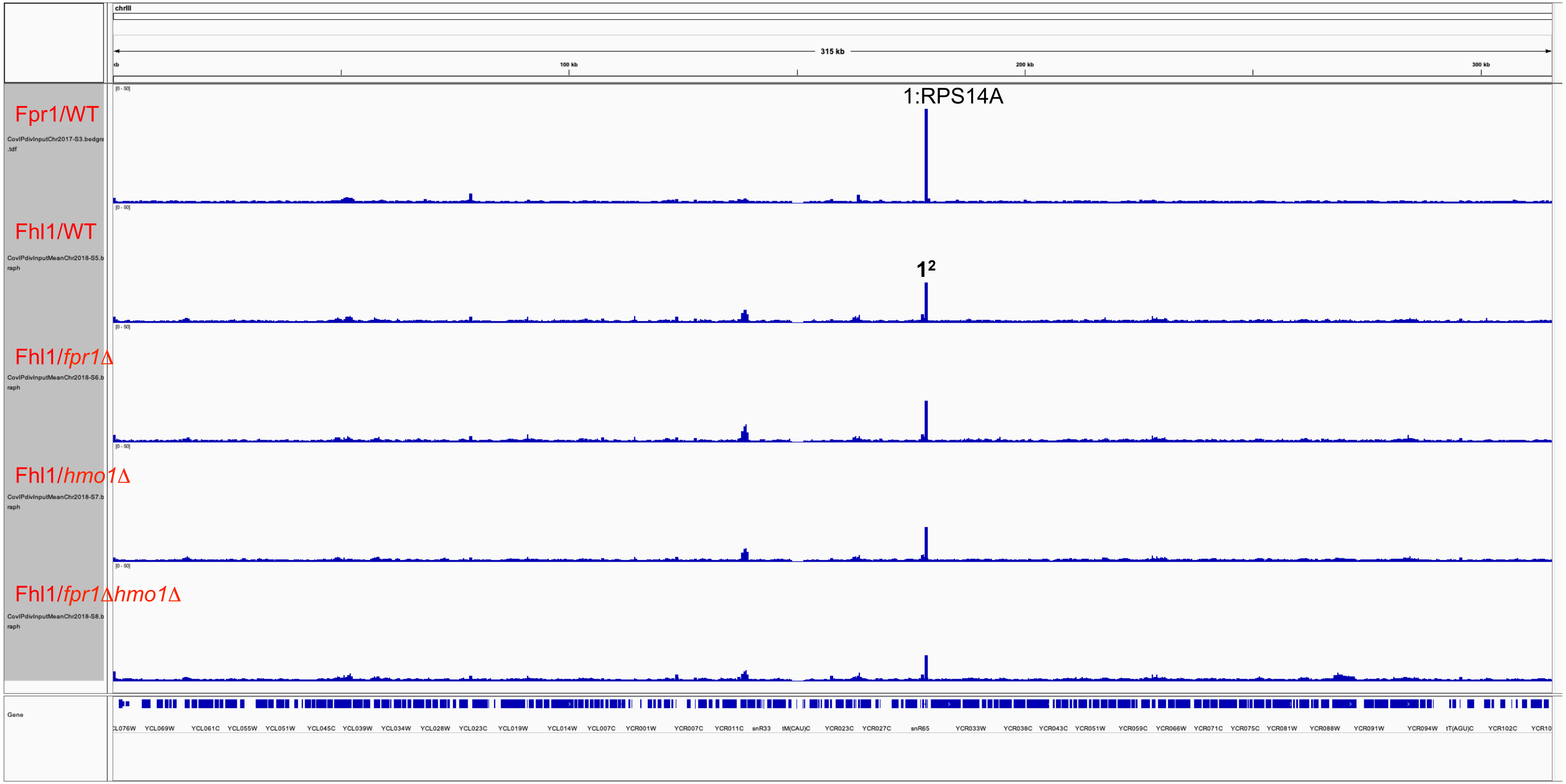

data range 50

black: Fhl1 binding is not influenced by *hmo1Δ/fpr1Δ*
red: Fhl1 binding is influenced by *fpr1Δ* but not by *hmo1Δ*
blue: Fhl1 binding is influenced by *hmo1Δ* but not by *fpr1Δ*
green: Fhl1 binding is influenced by *hmo1Δ/fpr1Δ*

Chr.4

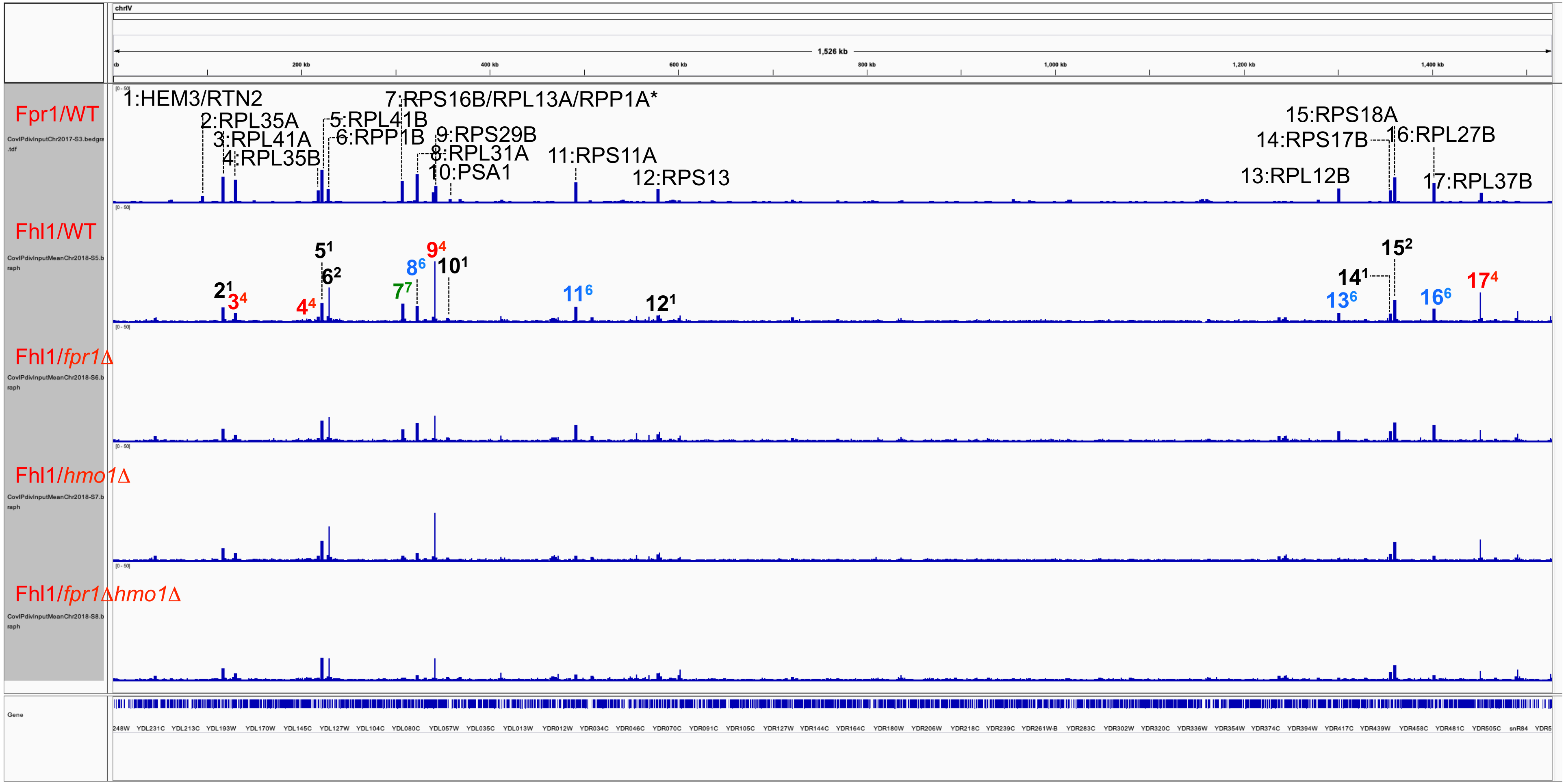

data range 50

black: Fhl1 binding is not influenced by *hmo1*Δ/*fpr1*Δ
red: Fhl1 binding is influenced by *fpr1*Δ but not by *hmo1*Δ
blue: Fhl1 binding is influenced by *hmo1*Δ but not by *fpr1*Δ
green: Fhl1 binding is influenced by *hmo1*Δ/*fpr1*Δ

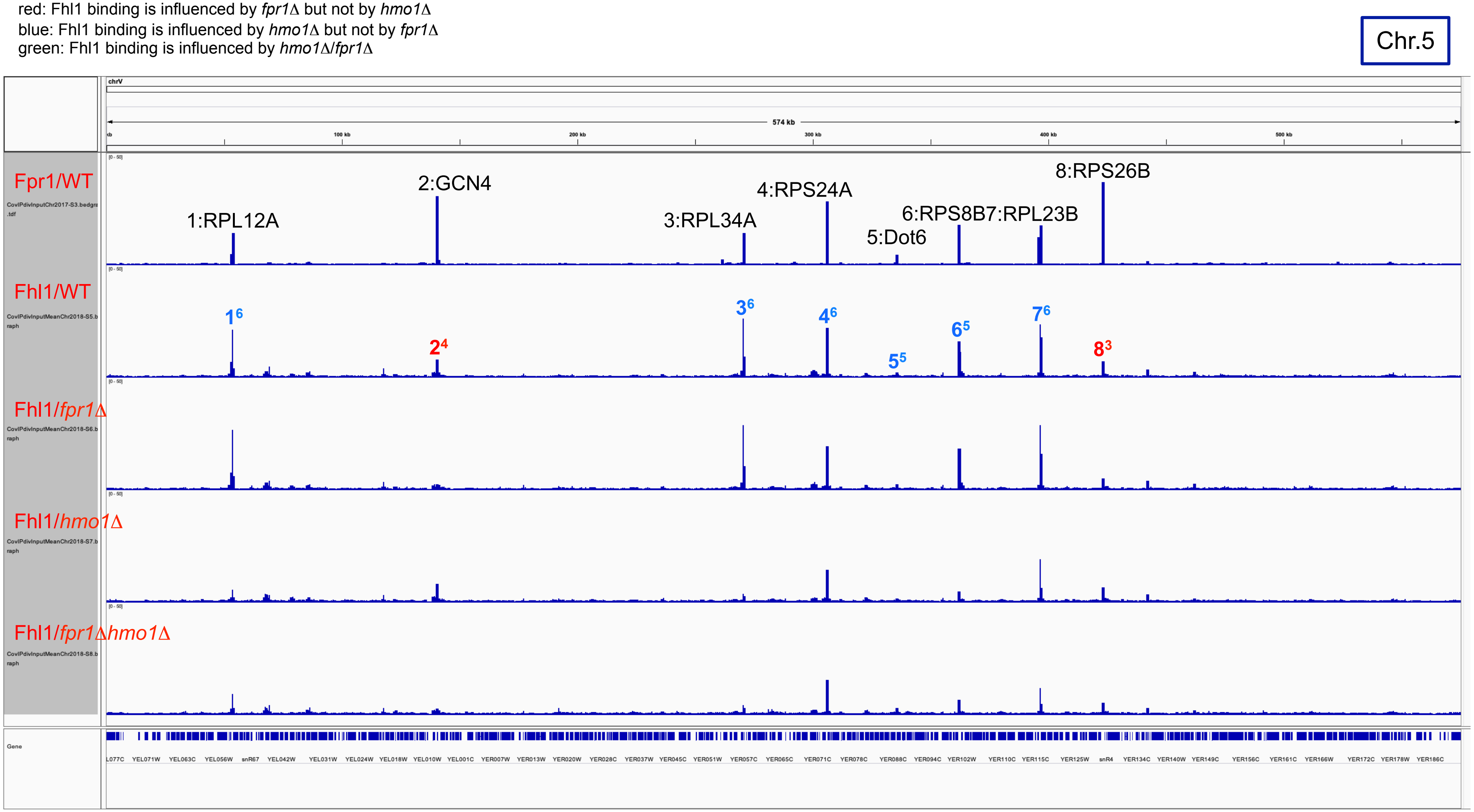

data range 50

black: Fhl1 binding is not influenced by *hmo1*Δ/*fpr1*Δ
red: Fhl1 binding is influenced by *fpr1*Δ but not by *hmo1*Δ
blue: Fhl1 binding is influenced by *hmo1*Δ but not by *fpr1*Δ
green: Fhl1 binding is influenced by *hmo1*Δ/*fpr1*Δ

Chr.6

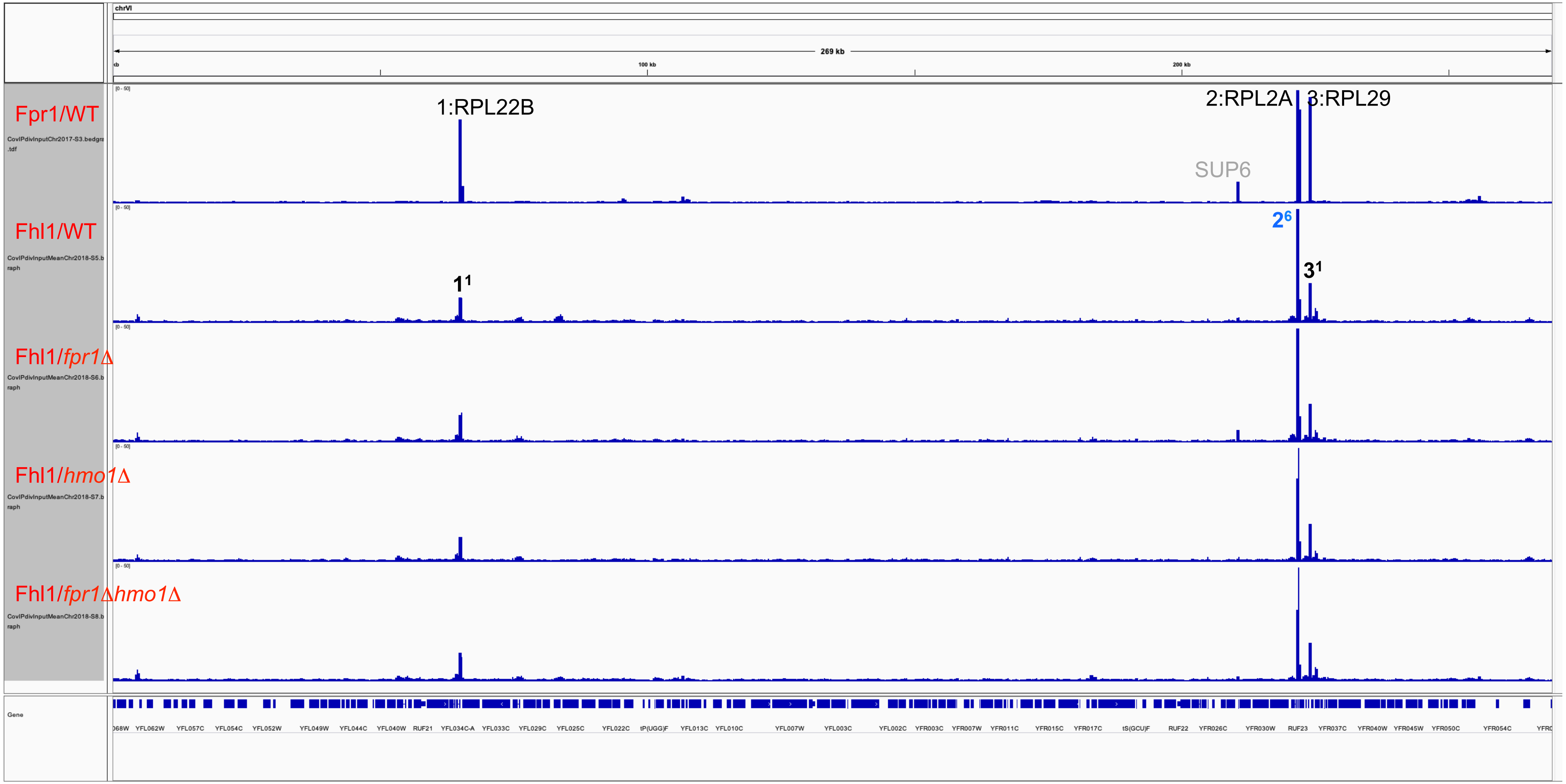

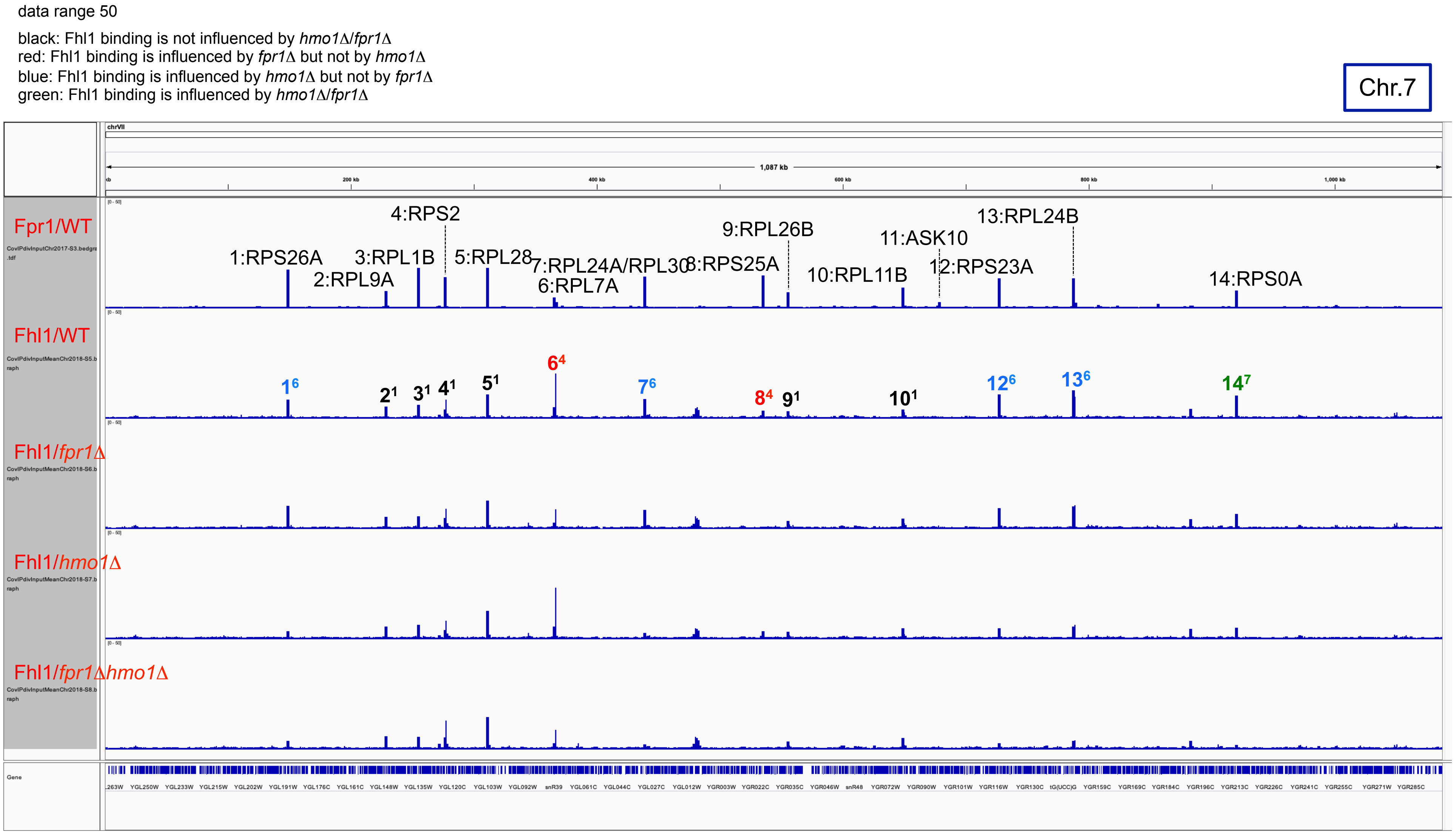
data range 50
black: Fhl1 binding is not influenced by hmo1Δ/fpr1Δ
red: Fhl1 binding is influenced by fpr1Δ but not by hmo1Δ
blue: Fhl1 binding is influenced by hmo1Δ but not by fpr1Δ
green: Fhl1 binding is influenced by hmo1Δ/fpr1Δ
Chr.7
chrVII
1,087 kb
200 kb
400 kb
600 kb
800 kb
1,000 kb
Fpr1/WT
4:RPS2
9:RPL26B
13:RPL24B
11:ASK10
1:RPS26A
3:RPL1B
5:RPL28
7:RPL24A/RPL30
8:RPS25A
12:RPS23A
10:RPL11B
14:RPS0A
2:RPL9A
6:RPL7A
Fhl1/WT
1[6]
2[1]
3[1]
4[1]
5[1]
6[4]
7[6]
8[4]
9[1]
10[1]
12[6]
13[6]
14[7]
Fhl1/fpr1Δ
Fhl1/hmo1Δ
Fhl1/fpr1Δhmo1Δ
Gene

data range 50

black: Fhl1 binding is not influenced by *hmo1Δ/fpr1Δ*
red: Fhl1 binding is influenced by *fpr1Δ* but not by *hmo1Δ*
blue: Fhl1 binding is influenced by *hmo1Δ* but not by *fpr1Δ*
green: Fhl1 binding is influenced by *hmo1Δ/fpr1Δ*

Chr.8

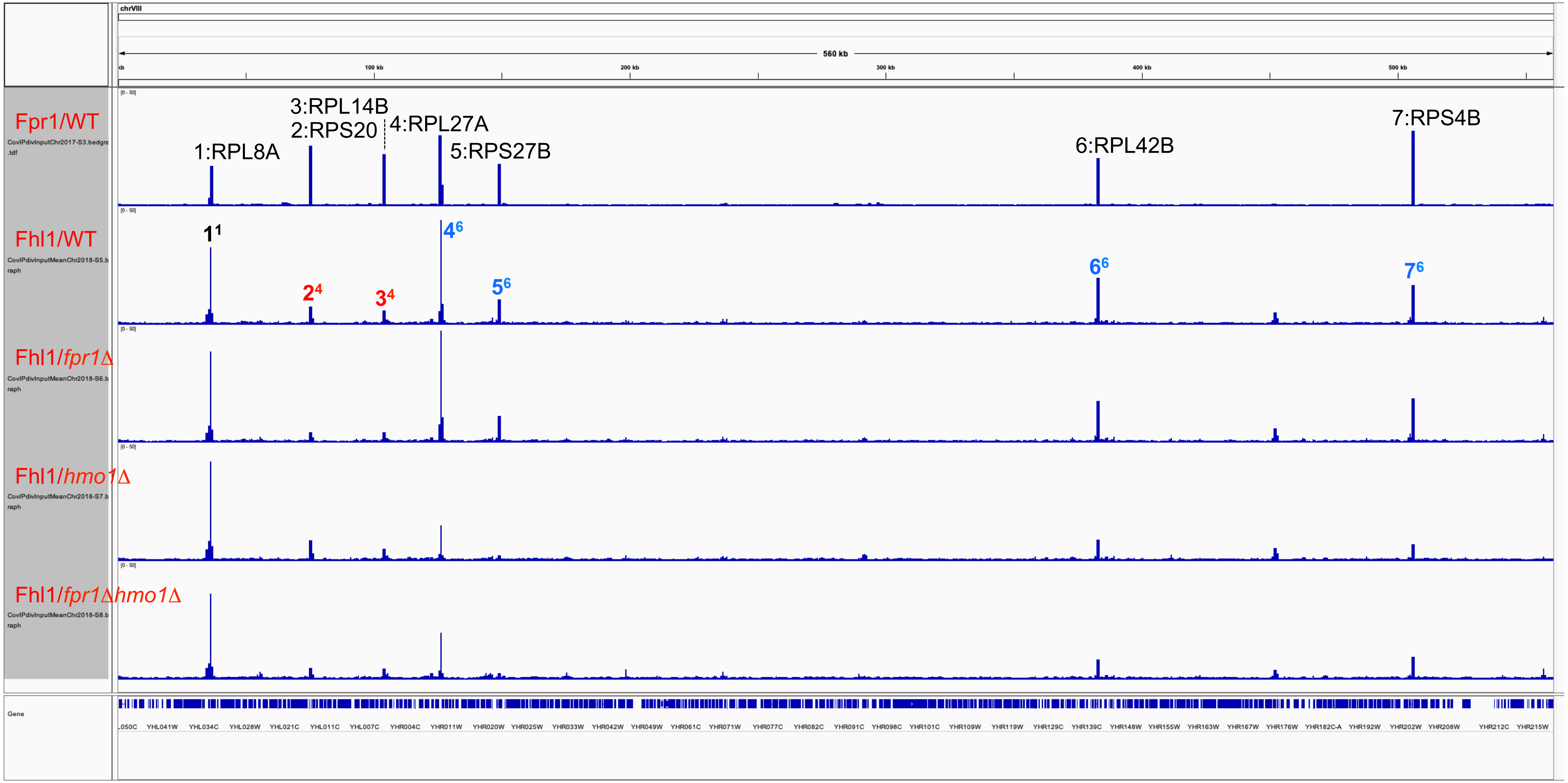

data range 50

black: Fhl1 binding is not influenced by *hmo1Δ/fpr1Δ*
red: Fhl1 binding is influenced by *fpr1Δ* but not by *hmo1Δ*
blue: Fhl1 binding is influenced by *hmo1Δ* but not by *fpr1Δ*
green: Fhl1 binding is influenced by *hmo1Δ/fpr1Δ*

Chr.9

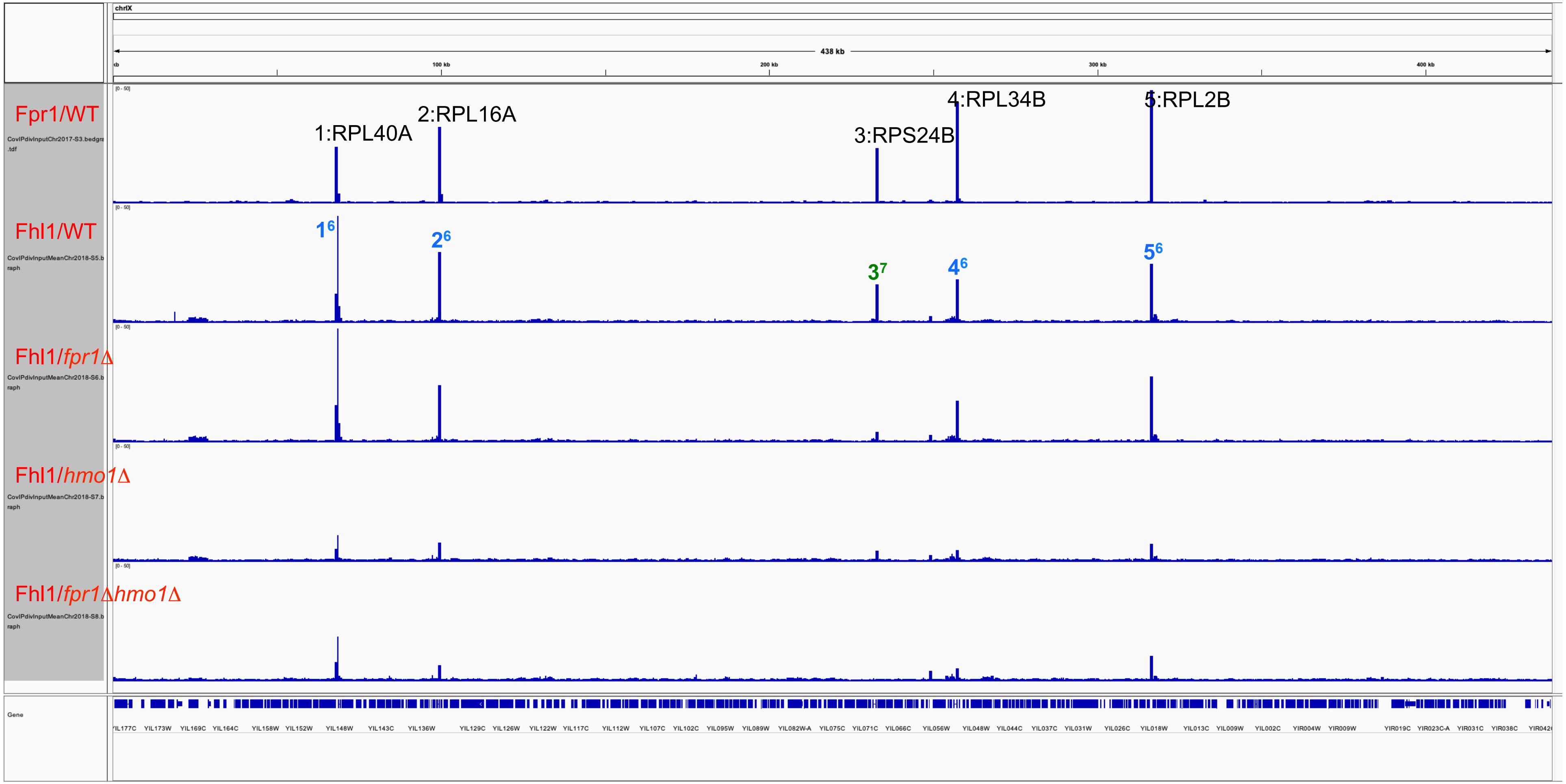

data range 50

black: Fhl1 binding is not influenced by *hmo1*Δ/*fpr1*Δ
red: Fhl1 binding is influenced by *fpr1*Δ but not by *hmo1*Δ
blue: Fhl1 binding is influenced by *hmo1*Δ but not by *fpr1*Δ
green: Fhl1 binding is influenced by *hmo1*Δ/*fpr1*Δ

Chr.10

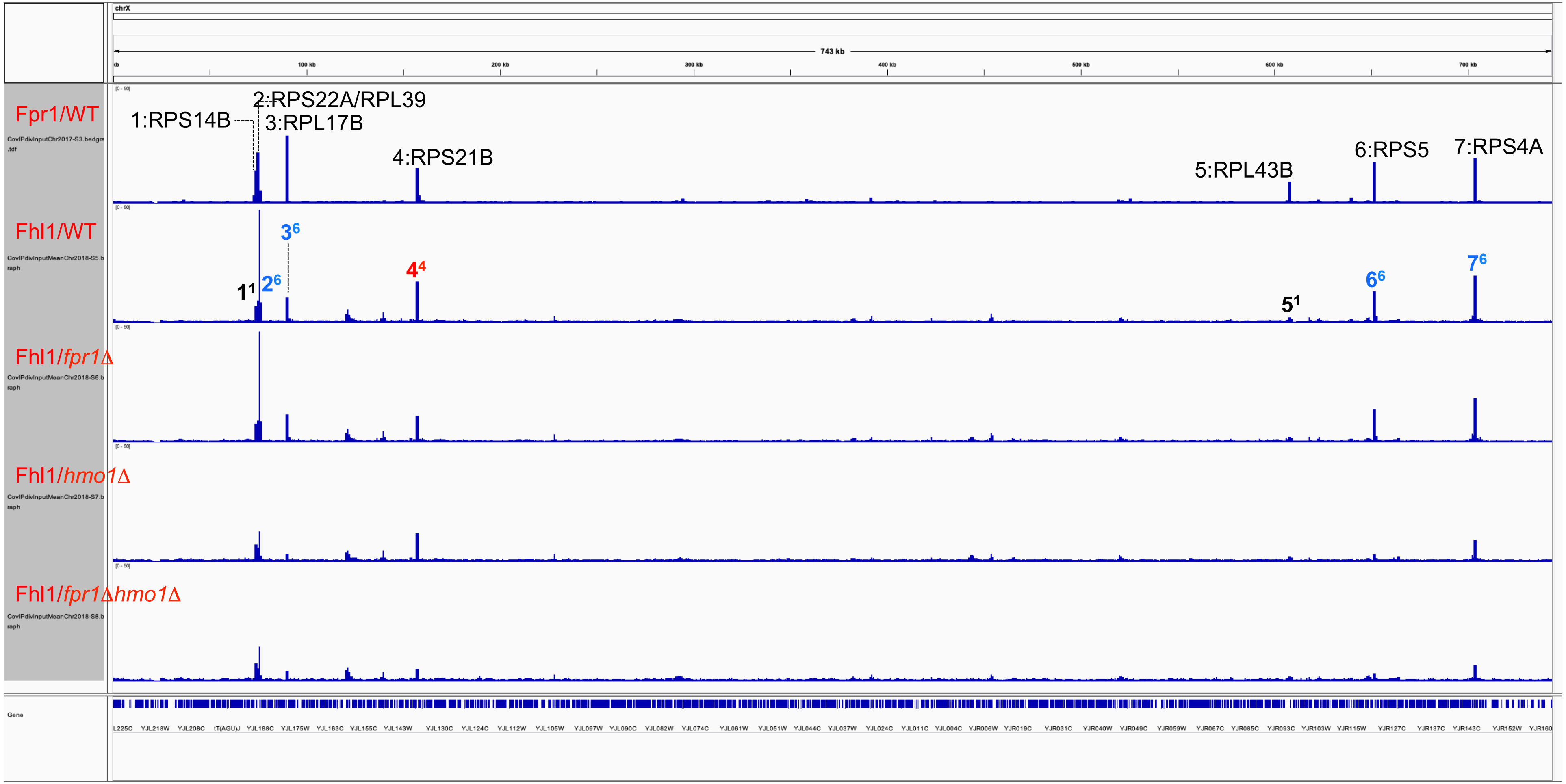

data range 50

black: Fhl1 binding is not influenced by *hmo1Δ/fpr1Δ*
red: Fhl1 binding is influenced by *fpr1Δ* but not by *hmo1Δ*
blue: Fhl1 binding is influenced by *hmo1Δ* but not by *fpr1Δ*
green: Fhl1 binding is influenced by *hmo1Δ/fpr1Δ*

Chr.11

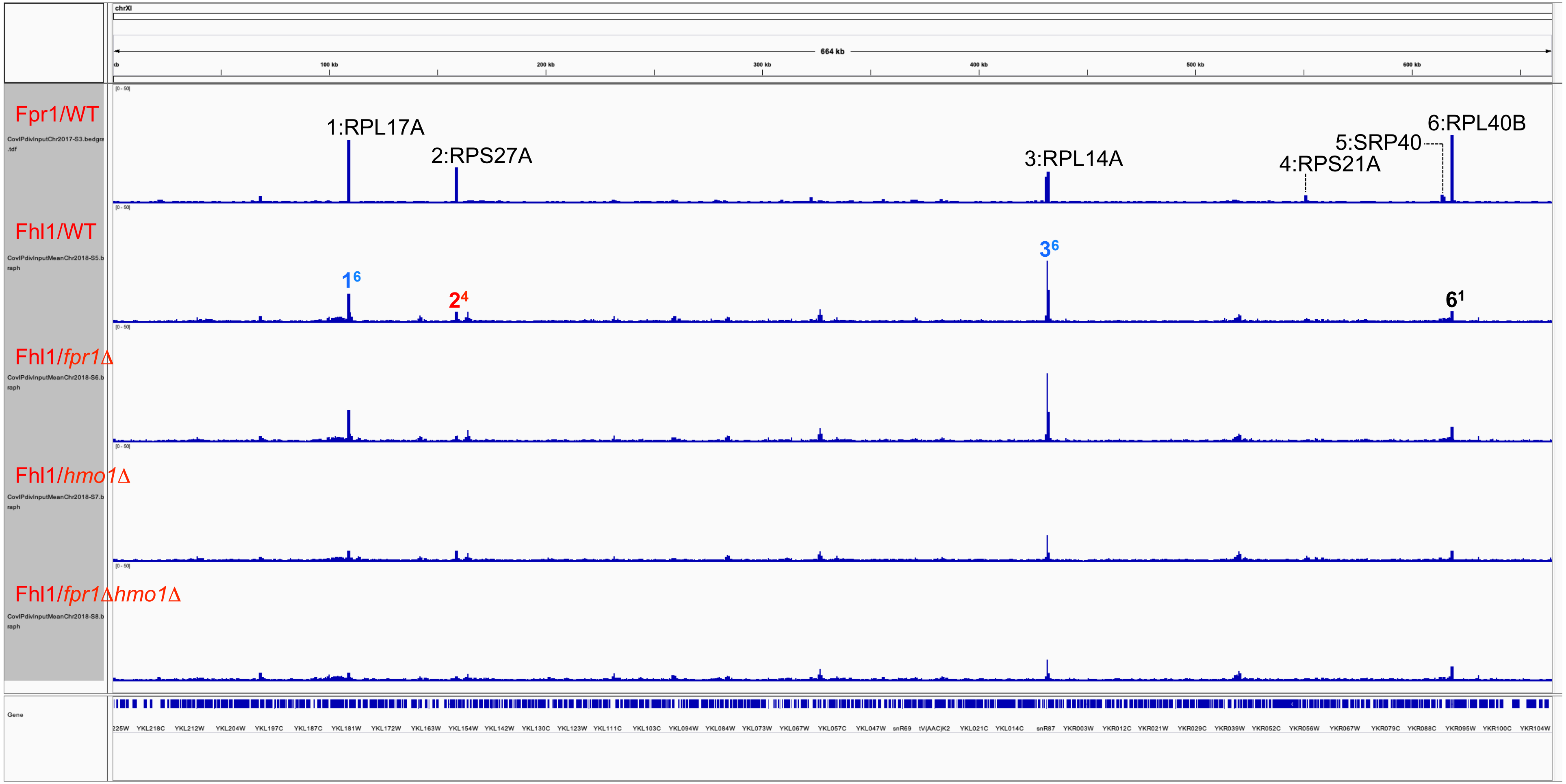

data range 50

black: Fhl1 binding is not influenced by *hmo1Δ/fpr1Δ*
red: Fhl1 binding is influenced by *fpr1Δ* but not by *hmo1Δ*
blue: Fhl1 binding is influenced by *hmo1Δ* but not by *fpr1Δ*
green: Fhl1 binding is influenced by *hmo1Δ/fpr1Δ*

Chr.12

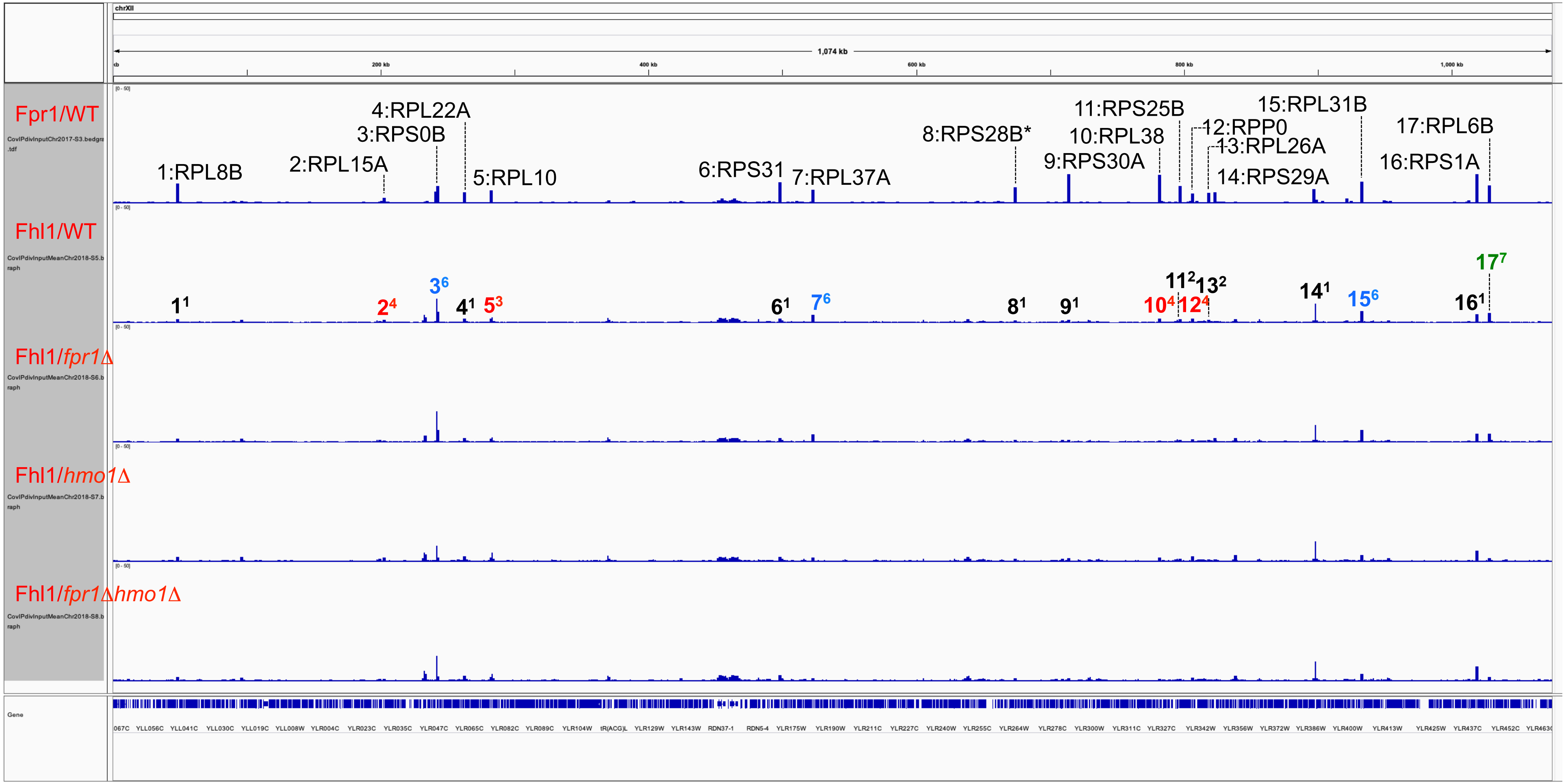

data range 50

black: Fhl1 binding is not influenced by *hmo1Δ/fpr1Δ*
red: Fhl1 binding is influenced by *fpr1Δ* but not by *hmo1Δ*
blue: Fhl1 binding is influenced by *hmo1Δ* but not by *fpr1Δ*
green: Fhl1 binding is influenced by *hmo1Δ/fpr1Δ*

Chr.13

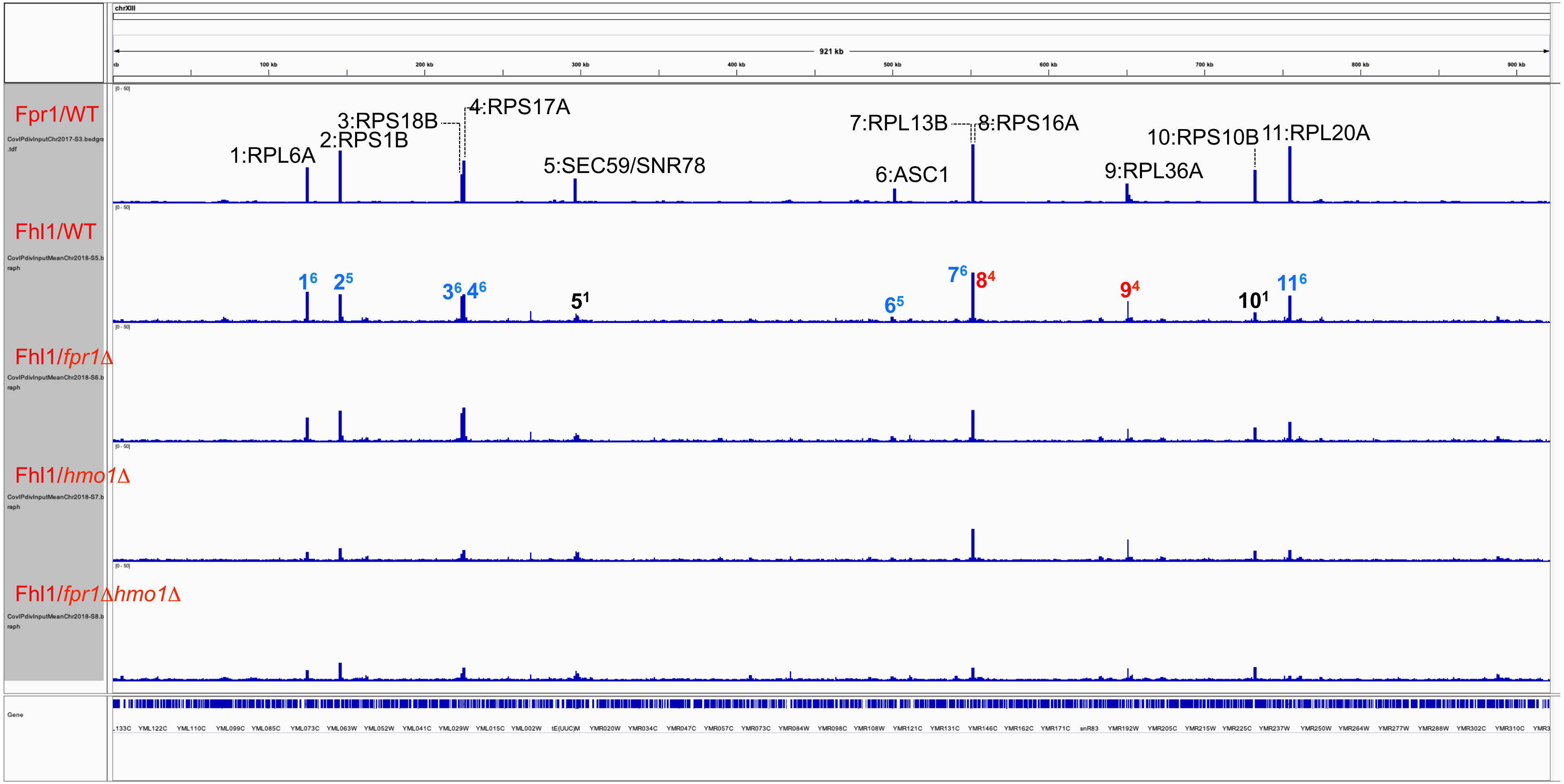

data range 50

black: Fhl1 binding is not influenced by *hmo1Δ/fpr1Δ*
red: Fhl1 binding is influenced by *fpr1Δ* but not by *hmo1Δ*
blue: Fhl1 binding is influenced by *hmo1Δ* but not by *fpr1Δ*
green: Fhl1 binding is influenced by *hmo1Δ/fpr1Δ*

Chr.14

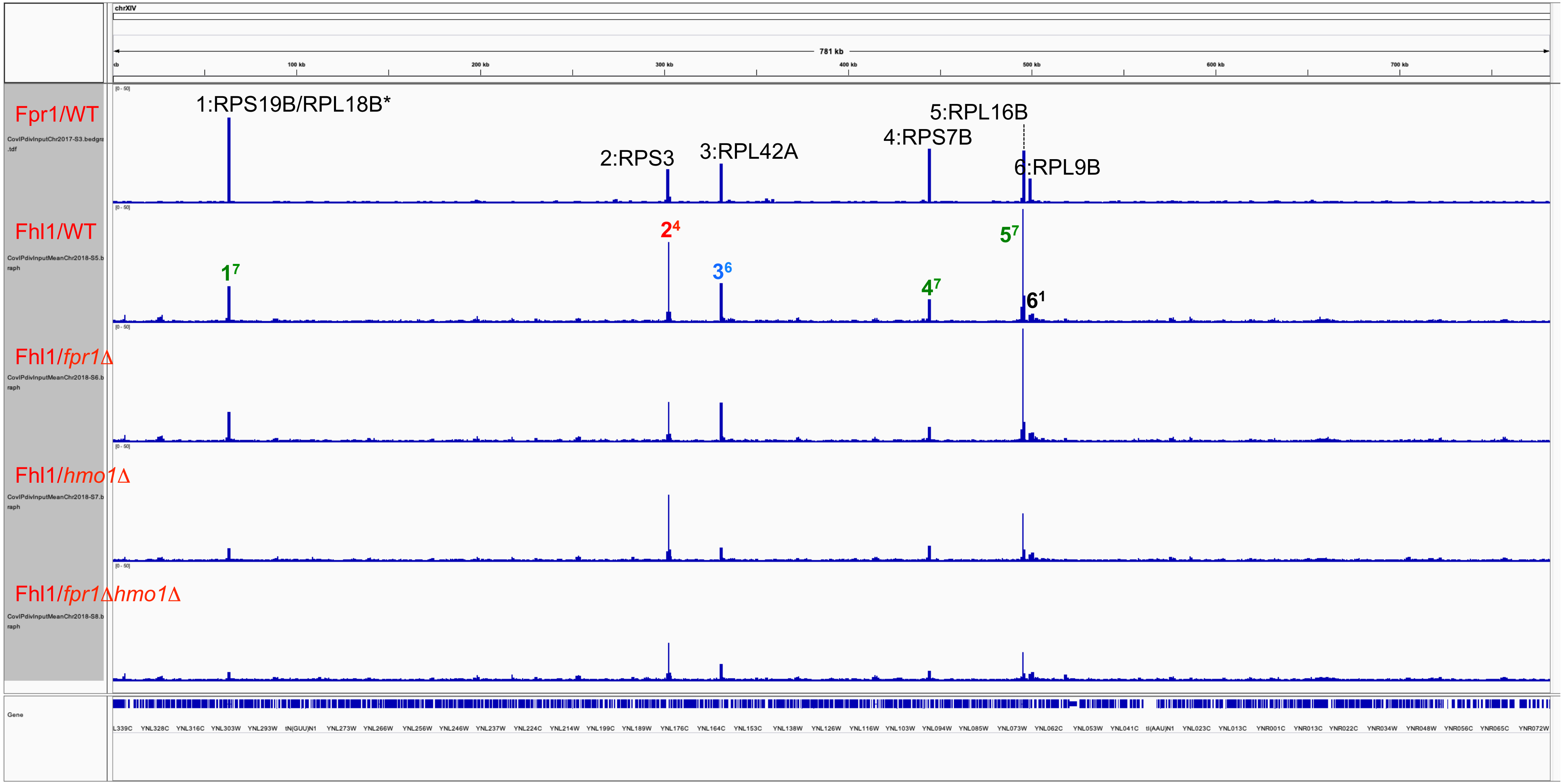

data range 50

black: Fhl1 binding is not influenced by *hmo1Δ/fpr1Δ*
red: Fhl1 binding is influenced by *fpr1Δ* but not by *hmo1Δ*
blue: Fhl1 binding is influenced by *hmo1Δ* but not by *fpr1Δ*
green: Fhl1 binding is influenced by *hmo1Δ/fpr1Δ*

Chr.15

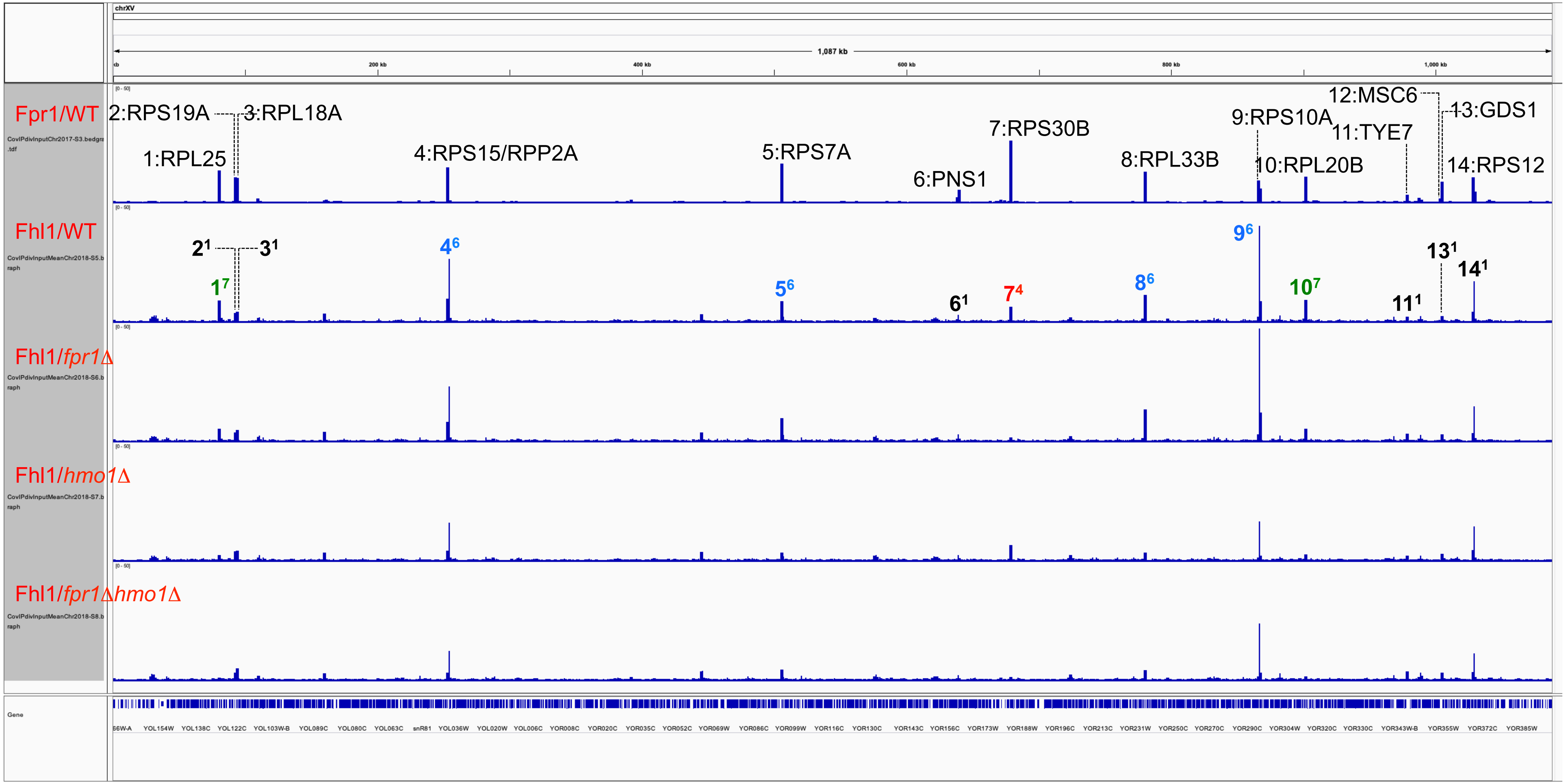

data range 50

black: Fhl1 binding is not influenced by *hmo1Δ/fpr1Δ*
red: Fhl1 binding is influenced by *fpr1Δ* but not by *hmo1Δ*
blue: Fhl1 binding is influenced by *hmo1Δ* but not by *fpr1Δ*
green: Fhl1 binding is influenced by *hmo1Δ/fpr1Δ*

Chr.16

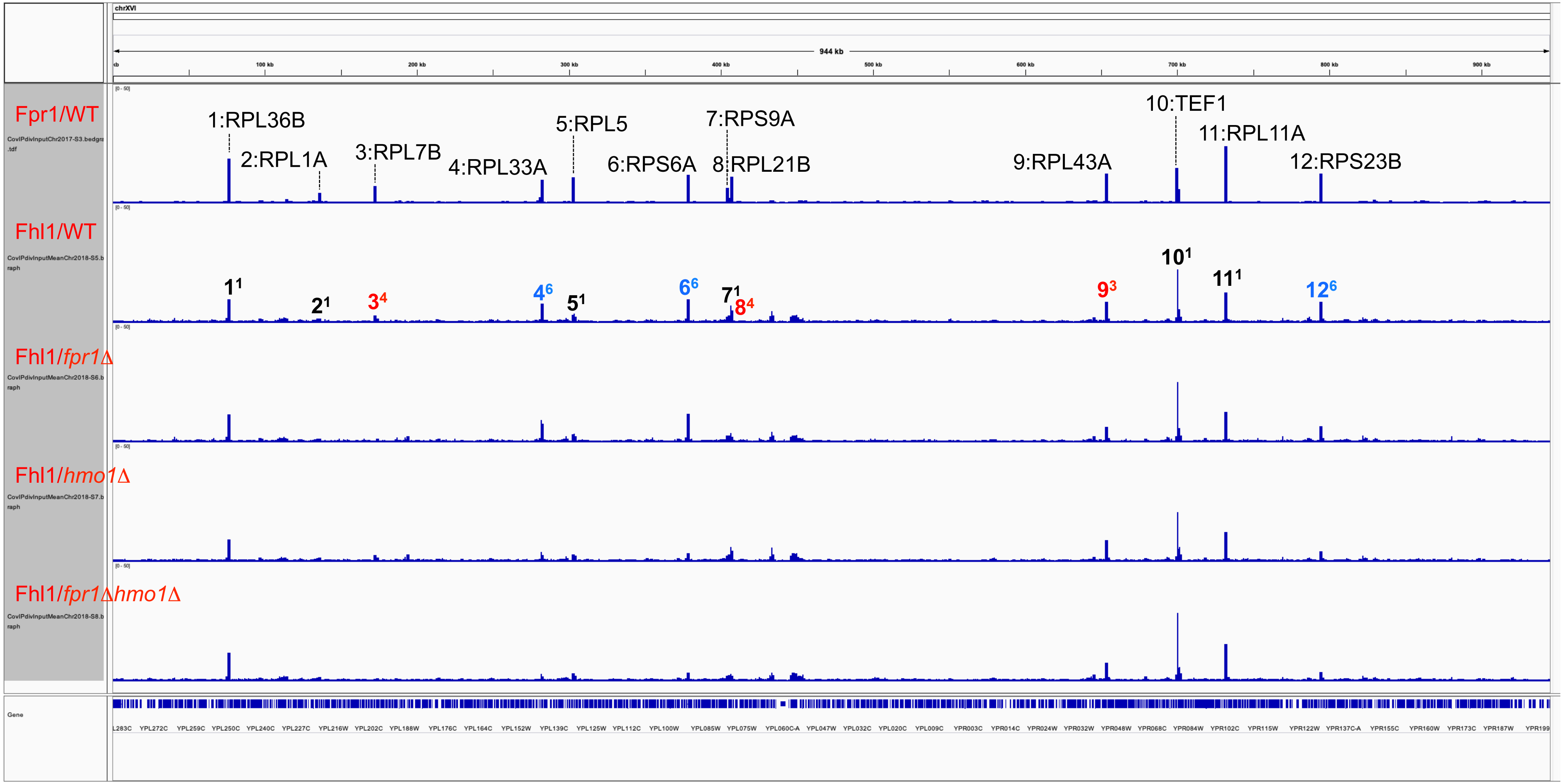

Supplement: S2 Fig — Binding positions of Fpr1 and Fhl1 (in WT, fpr1Δ, hmo1Δ, and hmo1Δfpr1Δ cells), identified by ChIP-seq, are summarised for each chromosome. Names of genes harbouring Fpr1-binding sites are shown in the top panel, and serial numbers are assigned within each chromosome. Fhl1-binding positions in WT, fpr1Δ, hmo1Δ, and hmo1Δfpr1Δ cells are shown in the second to fifth panels, as described on the left. The colours (black, red, blue, or green) of Fhl1-binding peaks/loci indicate the influence of deletion of HMO1 and/or FPR1 on Fhl1 binding to those loci. Superscripts of peak numbers correspond to the category numbers assigned to the peaks, as described in S3 Fig. (PDF) [file pgen.1008865.s002.pdf]
