# Supplementary material for: Fpr1, a primary target of rapamycin, functions as a transcription factor for ribosomal protein genes cooperatively with Hmo1 in Saccharomyces cerevisiae
Source: PLoS Genet. 2020 Jun 30;16(6):e1008865. doi: 10.1371/journal.pgen.1008865 (PMC7357790; doi:10.1371/journal.pgen.1008865)
Supplement: S3 Fig — Amounts of Fhl1 binding to its target loci, which were measured by ChIP-seq, are presented as bar graphs. Bars featuring four colours, blue, red, yellow, and green, represent Fhl1 binding to the genes in each panel in WT, fpr1Δ, hmo1Δ, and hmo1Δfpr1Δ cells, respectively. Genes within red or blue boxes were also examined in individual ChIP assays, shown in Fig 3B or S4 Fig, respectively. ChIP-assay values are expressed as ratios relative to values obtained for WT cells. Fpr1-target genes harbouring Fhl1-binding sites are classified into four groups according to the influence of deletion of HMO1 and/or FPR1 on Fhl1 binding: a: not influenced by deletion of HMO1/FPR1 (black symbol); b: influenced by deletion of FPR1, but not by that of HMO1 (red symbol); c: influenced by deletion of HMO1, but not by that of FPR1 (blue symbol); and d: influenced by deletion of HMO1/FPR1 (green symbol). Coloured symbols at the top of bar graphs reflect this classification. Groups a, b, and c are further divided into two categories. These classifications are summarised in the table at the bottom of this figure and explained in the text. (PDF) [file pgen.1008865.s003.pdf]

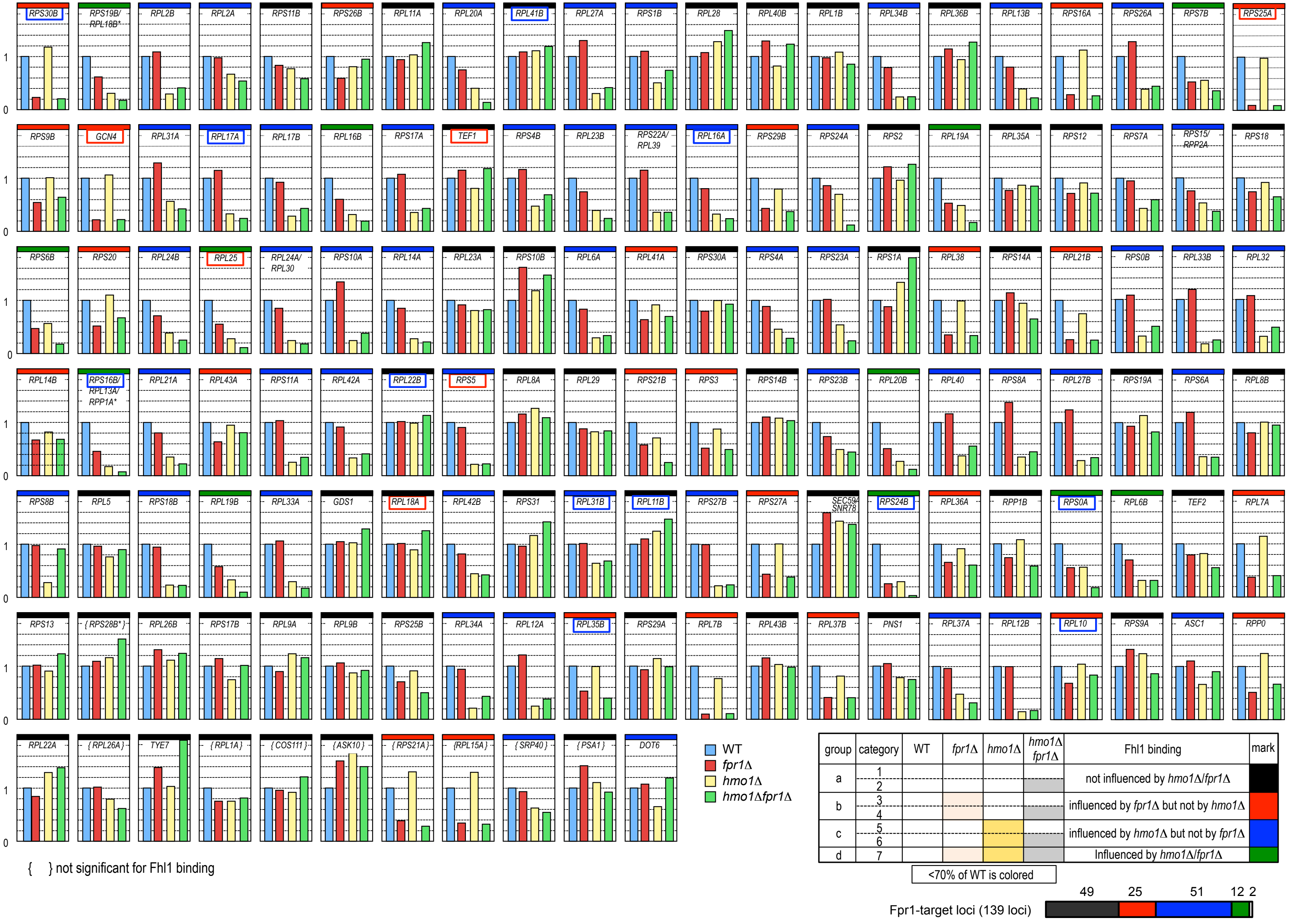

S3 Fig. Influence of deletion of *HMO1* and/or *FPR1* on Fhl1 binding to its target loci.

Amounts of Fhl1 binding to its target loci, which were measured by ChIP-seq, are presented as bar graphs. Bars featuring four colours, blue, red, yellow, and green, represent Fhl1 binding to the genes in each panel in WT, *fpr1*Δ, *hmo1*Δ, and *hmo1*Δ*fpr1*Δ cells, respectively. Genes within red or blue boxes were also examined in individual ChIP assays, shown in Fig 3B or S3 Fig, respectively. ChIP-assay values are expressed as ratios relative to values obtained for WT cells. Fpr1 target genes harbouring Fhl1-binding sites are classified into four groups according to the influence of deletion of *HMO1* and/or *FPR1* on Fhl1 binding: **a**: not influenced by deletion of *HMO1/FPR1* (black symbol); **b**: influenced by deletion of *FPR1*, but not by that of *HMO1* (red symbol); **c**: influenced by deletion of *HMO1*, but not by that of *FPR1* (blue symbol); and **d**: influenced by deletion of *HMO1/FPR1* (green symbol). Coloured symbols at the top of bar graphs reflect this classification. Groups **a**, **b,** and **c** are further divided into two categories. These classifications are summarised in the table at the bottom of this figure and explained in the text.
