# Supplementary material for: Fpr1, a primary target of rapamycin, functions as a transcription factor for ribosomal protein genes cooperatively with Hmo1 in Saccharomyces cerevisiae
Source: PLoS Genet. 2020 Jun 30;16(6):e1008865. doi: 10.1371/journal.pgen.1008865 (PMC7357790; doi:10.1371/journal.pgen.1008865)
Supplement: S4 Fig — To examine the influence of deletion of HMO1 and/or FPR1 on Fhl1 binding to RPG promoters, ChIP assays were conducted for additional Fpr1-target genes as described in Fig 3B. Coloured symbols at the top of each panel reflect the classification of Fpr1-target genes, as described in S3 Fig. (PDF) [file pgen.1008865.s004.pdf]

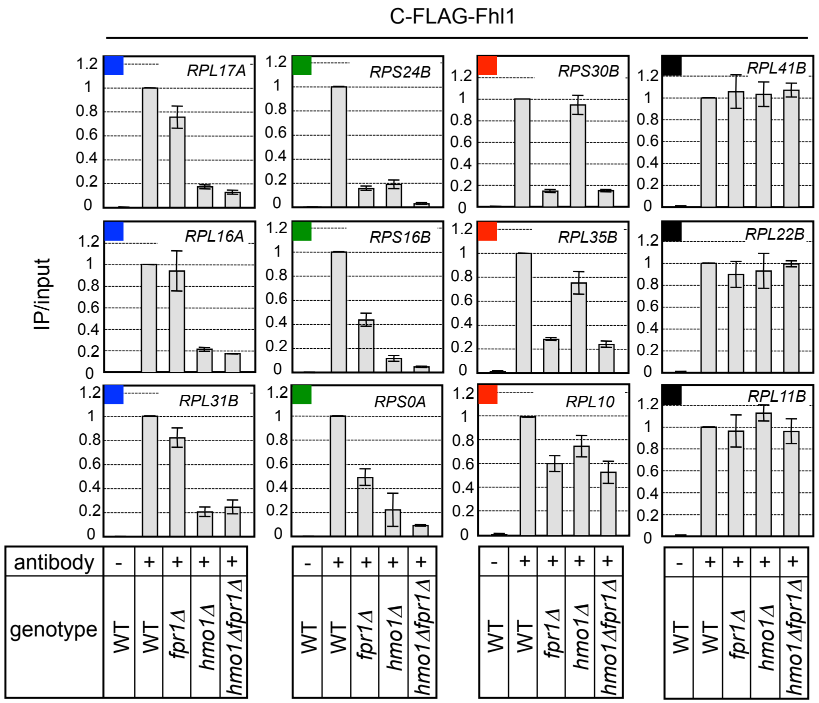

S4 Fig. Effect of deletion of *HMO1* and/or *FPR1* on Fhl1 binding to specific RPG promoters.
To examine the influence of deletion of *HMO1* and/or *FPR1* on Fhl1 binding to RPG promoters, ChIP assays were conducted for additional Fpr1-target genes as described in Fig 3B. The coloured symbols at the top of each panel reflect the classification of Fpr1 target genes as described in S3 Fig.
