# Supplementary material for: Fpr1, a primary target of rapamycin, functions as a transcription factor for ribosomal protein genes cooperatively with Hmo1 in Saccharomyces cerevisiae
Source: PLoS Genet. 2020 Jun 30;16(6):e1008865. doi: 10.1371/journal.pgen.1008865 (PMC7357790; doi:10.1371/journal.pgen.1008865)
Supplement: S5 Fig — (A) Rapid depletion analysis of Fpr1 using the AID degron system. Two fpr1Δ strains expressing C-terminally FLAG-tagged Fhl1, expressing or not expressing Oryza sativa TIR1, were transformed with empty plasmid (indicated as “–”), plasmid expressing untagged-Fpr1 (–AID), or plasmid expressing C-terminally AID-tagged-Fpr1 (+AID). Depletion of Fpr1 was achieved by addition of indole-3-acetic acid (IAA, 0.5 mM) for 60 min, as described in the Methods section. The amount of Fpr1 proteins was measured by immunoblotting using anti-Fpr1 antibody. (B) Fhl1 binding to the promoters of RPS25A, RPS30B, and RPS5 was examined by ChIP assays using yeast cells in which Fpr1 was depleted or not, as described in (A). (PDF) [file pgen.1008865.s005.pdf]

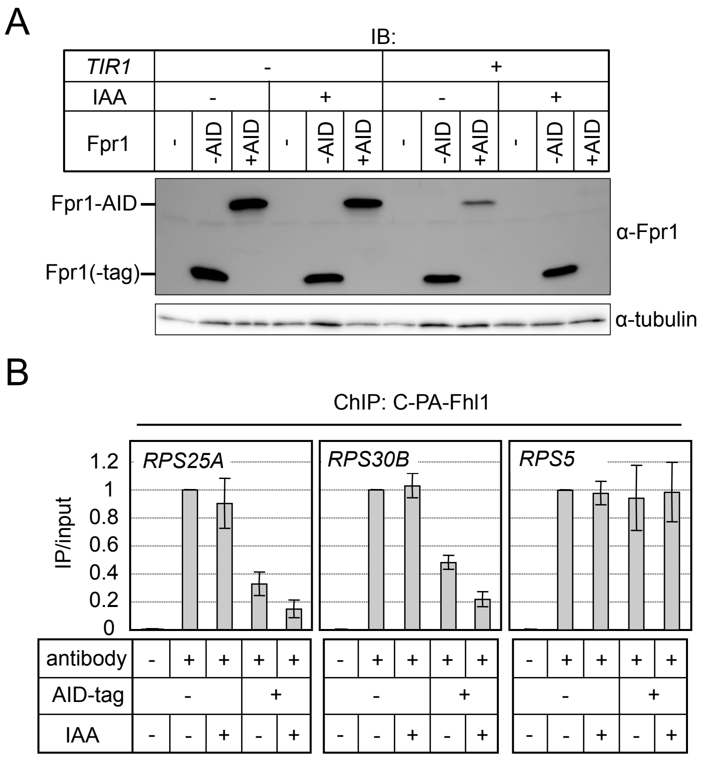


**S5 Fig. Effect of rapid depletion of Fpr1 on Fhl1 binding to specific RPG promoters.**
(A) Rapid depletion analysis of Fpr1 using the AID degron system. Two *fpr1*Δ strains expressing C-terminally FLAG-tagged Fhl1, expressing or not expressing *Oryza sativa TIR1*, were transformed with empty plasmid (indicated as "–"), plasmid expressing untagged-Fpr1 (–AID), or plasmid expressing C-terminally AID-tagged-Fpr1 (+AID). Depletion of Fpr1 was achieved by addition of indole-3-acetic acid (IAA, 0.5 mM) for 60 min, as described in the Methods section. The amount of Fpr1 proteins was measured by immunoblotting using anti-Fpr1 antibody. (B) Fhl1 binding to the promoters of *RPS25A*, *RPS30B*, and *RPS5* was examined by ChIP assays using yeast cells in which Fpr1 was depleted or not, as described in (A).
