# Supplementary material for: Fpr1, a primary target of rapamycin, functions as a transcription factor for ribosomal protein genes cooperatively with Hmo1 in Saccharomyces cerevisiae
Source: PLoS Genet. 2020 Jun 30;16(6):e1008865. doi: 10.1371/journal.pgen.1008865 (PMC7357790; doi:10.1371/journal.pgen.1008865)
Supplement: S6 Fig — Precise binding sites of Fpr1 and Fhl1 (in WT, fpr1Δ, hmo1Δ, and hmo1Δfpr1Δ cells) at the promoters of RPL25, RPS5, and RPS25A, which were identified by ChIP-seq; IGV_2.4.8 software was used for depicting the sites. Red and blue dashed lines indicate the binding peaks of Fpr1 and Fhl1 in WT cells. The grey rectangles, black arrows, and red asterisks indicate the coding region, transcriptional direction, and position of a start codon, of each RPG, respectively. (PDF) [file pgen.1008865.s006.pdf]

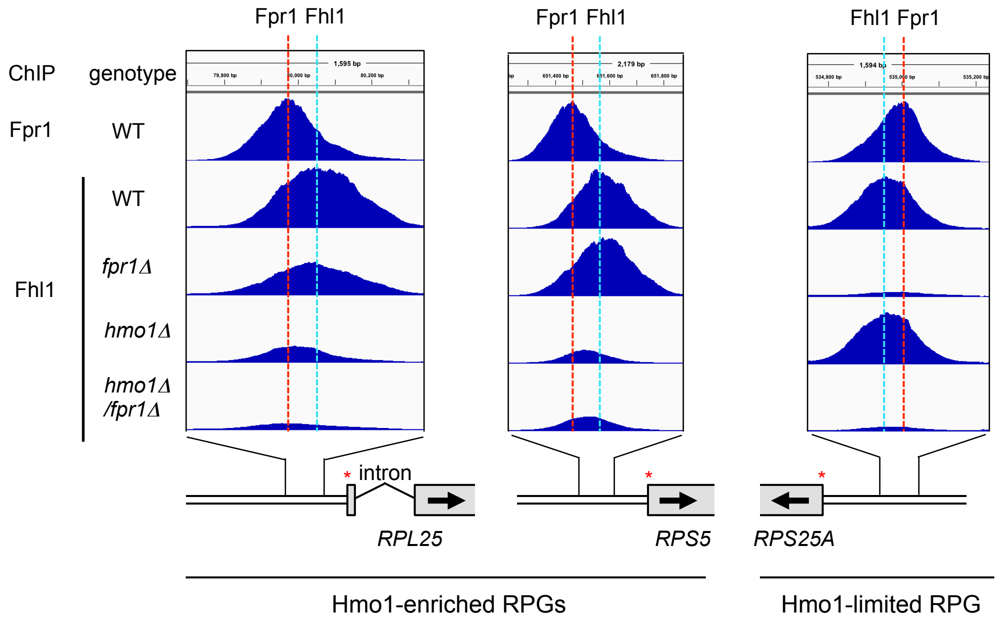

S6 Fig. Binding positions of Fpr1 and Fhl1 on their target RPG promoters.
Precise binding sites of Fpr1 and Fhl1 (in WT, *fpr1*Δ, *hmo1*Δ, and *hmo1*Δ*fpr1*Δ cells) at the promoters of *RPL25*, *RPS5*, and *RPS25A*, which were identified by ChIP-seq; IGV_2.4.8 software was used for depicting the sites. Red and blue dashed lines indicate the binding peaks of Fpr1 and Fhl1 in WT cells. The grey rectangles, black arrows, and red asterisks indicate the coding region, transcriptional direction, and position of a start codon, of each RPG, respectively.
