# Supplementary material for: Fpr1, a primary target of rapamycin, functions as a transcription factor for ribosomal protein genes cooperatively with Hmo1 in Saccharomyces cerevisiae
Source: PLoS Genet. 2020 Jun 30;16(6):e1008865. doi: 10.1371/journal.pgen.1008865 (PMC7357790; doi:10.1371/journal.pgen.1008865)
Supplement: S7 Fig — Yeast cells expressing both N-terminally PA-tagged Fpr1 and C-terminally FLAG-tagged Fhl1 were treated with rapamycin (+: 80 nM, ++: 400 nM) for 60 min, and subjected to ChIP assay using anti-PA-tag antibody or anti-FLAG-tag antibody. (PDF) [file pgen.1008865.s007.pdf]

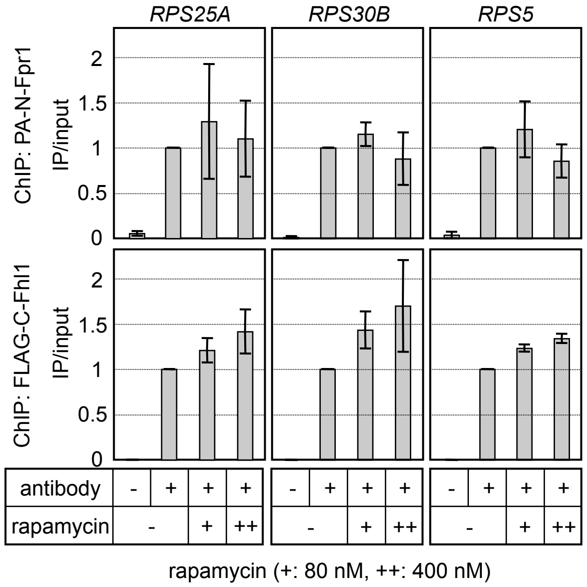


**S7 Fig. Effect of rapamycin on the binding of Fpr1 and Fhl1 to three RPG promoters.**
Yeast cells expressing both N-terminally PA-tagged Fpr1 and C-terminally FLAG-tagged Fhl1 were treated with rapamycin (+: 80 nM, ++: 400 nM) for 60 min, and subjected to ChIP assay using anti-PA-tag antibody or anti-FLAG-tag antibody.
