# Supplementary material for: Fpr1, a primary target of rapamycin, functions as a transcription factor for ribosomal protein genes cooperatively with Hmo1 in Saccharomyces cerevisiae
Source: PLoS Genet. 2020 Jun 30;16(6):e1008865. doi: 10.1371/journal.pgen.1008865 (PMC7357790; doi:10.1371/journal.pgen.1008865)
Supplement: S8 Fig — (A) RNA-seq analyses were conducted to examine the effects of deletion of HMO1 and/or FPR1 on genome-wide transcription as described in Fig 4A. The values obtained for RNA levels of genes in the entire genome were expressed as a ratio to the value measured for WT cells, aligned in the descending order of values for hmo1Δfpr1Δ cells, and are summarised here as a heatmap. In this heatmap, maximal and minimal values for fold-changes were set at +5 and –5, respectively, and FDR p-value of <0.05 was used as the cut-off. (B) RPGs are marked here with green lines in the heatmap from (A). (PDF) [file pgen.1008865.s008.pdf]

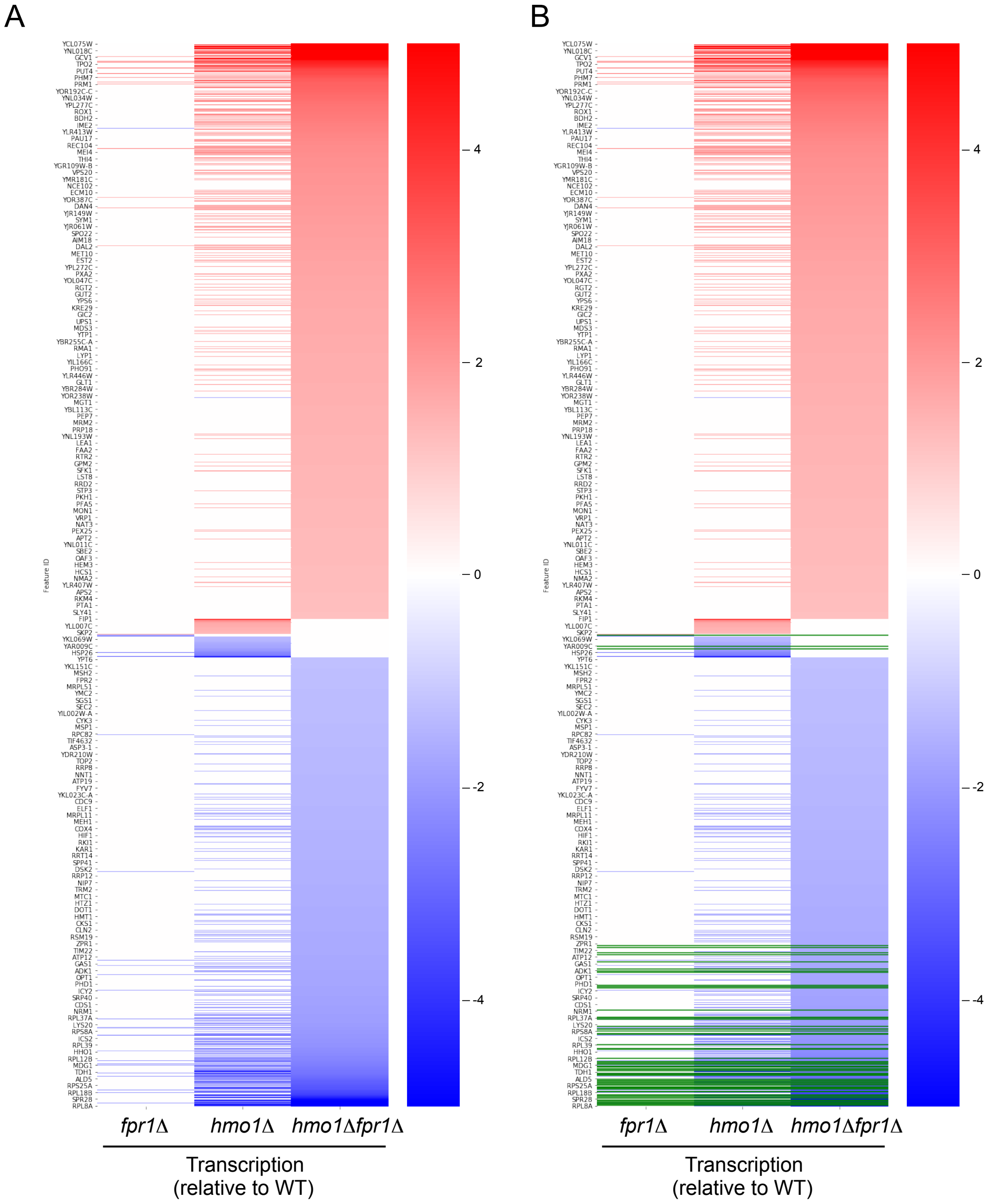

S8 Fig. Effect of deletion of *HMO1* and/or *FPR1* on genome-wide transcription.
(A) RNA-seq analyses were conducted to examine the effects of deletion of *HMO1* and/or *FPR1* on genome-wide transcription as described in Fig 4A. The values obtained for RNA levels of genes in the entire genome were expressed as a ratio to the value measured for WT cells, aligned in the descending order of values for *hmo1*Δ*fpr1*Δ cells, and are summarised here as a heatmap. In this heatmap, maximal and minimal values for fold-changes were set at +5 and –5, respectively, and FDR *p*-value of <0.05 was used as the cut-off. (B) RPGs are marked here with green lines in the heatmap from (A).
