# Supplementary material for: Fpr1, a primary target of rapamycin, functions as a transcription factor for ribosomal protein genes cooperatively with Hmo1 in Saccharomyces cerevisiae
Source: PLoS Genet. 2020 Jun 30;16(6):e1008865. doi: 10.1371/journal.pgen.1008865 (PMC7357790; doi:10.1371/journal.pgen.1008865)
Supplement: S9 Fig — To evaluate the correlation between the effects of deletion of HMO1 and/or FPR1 on Fhl1 binding to and transcription of Fpr1-target genes, the heatmap in Fig 4A was modified as follows. The Hmo1-binding results are ChIP-seq data quoted from Reja et al. [30]. Fhl1 binding to each gene in WT was set as 1, and the relative strength of Fhl1 binding to the same gene in other strains (fpr1Δ, hmo1Δ, and hmo1Δfpr1Δ) was calculated as a ratio to that of WT cells. The data were reanalysed by using the hierarchical clustering method according to the binding profiles of Fhl1 and are summarised as a heatmap. Coloured symbols attached to each gene show the influence of deletion of HMO1/FPR1 on Fhl1 binding, as described in S3 Fig. (PDF) [file pgen.1008865.s009.pdf]

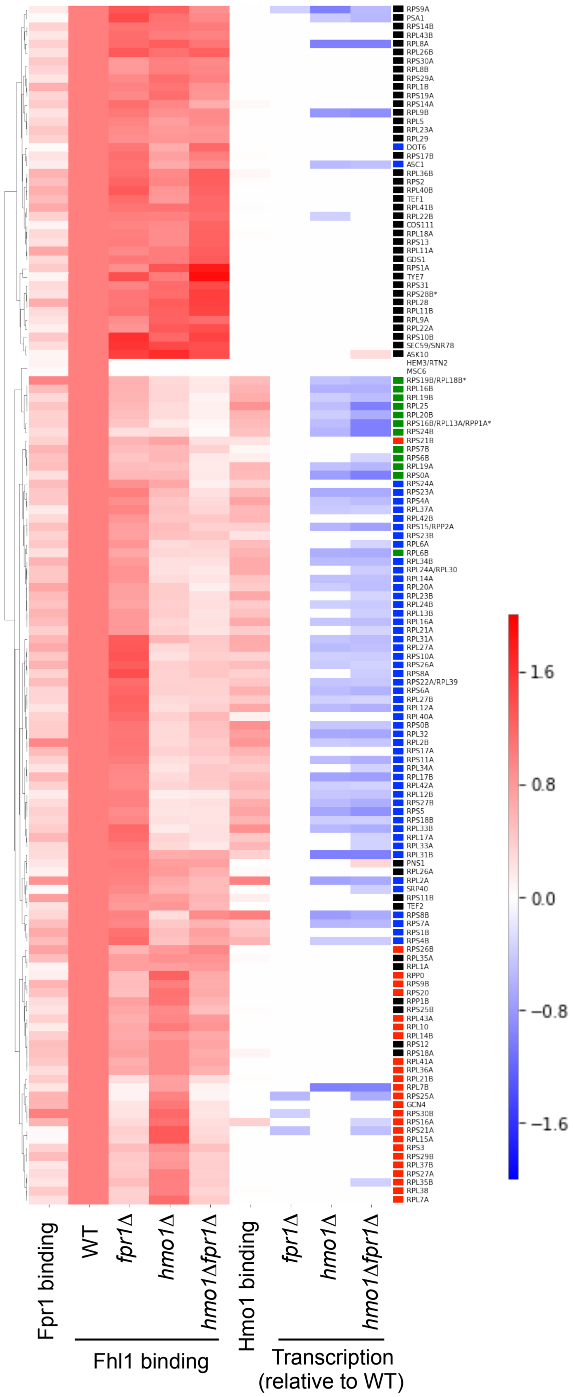

S9 Fig. Effect of deletion of Hmo1 and/or Fpr1 on transcription and Fhl1 binding of Fpr1-target genes.

To evaluate the correlation between the effects of deletion of *HMO1* and/or *FPR1* on Fhl1 binding to and transcription of Fpr1-target genes, the heatmap in Fig 4A was modified as follows. The Hmo1-binding results are ChIP-seq data quoted from Reja et al. [30]. Fhl1 binding to each gene in WT was set as 1, and the relative strength of Fhl1 binding to the same gene in other strains (*fpr1*Δ, *hmo1*Δ, and *hmo1*Δ*fpr1*Δ) was calculated as a ratio to that of WT cells. The data were reanalysed by using the hierarchical clustering method according to the binding profiles of Fhl1 and are summarised as a heatmap. Coloured symbols attached to each gene show the influence of deletion of *HMO1*/*FPR1* on Fhl1 binding, as described in S3 Fig.
