# Supplementary material for: Fpr1, a primary target of rapamycin, functions as a transcription factor for ribosomal protein genes cooperatively with Hmo1 in Saccharomyces cerevisiae
Source: PLoS Genet. 2020 Jun 30;16(6):e1008865. doi: 10.1371/journal.pgen.1008865 (PMC7357790; doi:10.1371/journal.pgen.1008865)
Supplement: S1 Text — (DOCX) [file pgen.1008865.s030.docx]

**Yeast strains**

The yeast strains used in this study are listed in S1 Table. The strains 6864 (*tor1*Δ) and 25053 (*TOR2*/*tor2*Δ) were purchased from Euroscarf. The H2451 (wild-type) [1] and YKK16 (wild-type) [2] were described previously. YKK97 (*hmo1*Δ) were generated by tetrad dissection from YKK94, which was described previously [2].

The *FPR1* gene of H2451 or YKK16 was disrupted by transforming a DNA fragment amplified from pFA6a-kanMX6 [3] by PCR using TK4806-TK4807 primers, and was selected on YPD plate containing G418 (200 μg/ml) to generate YKK166 and YKK168, respectively. H2451 was transformed with a DNA fragment amplified from pM5791[4] by PCR using KK169-TK4807 primers, and was selected on SD–His (synthetic dextrose medium plate lacking histidine) to generate YKK306, a strain expressing C-terminally FLAG (3×)-tagged Fpr1*.* YKK97 was crossed with YKK168 to generate wild-type cells (YKK208, YKK461, and YKK468), *fpr1*Δ cells (YKK207, YKK455, and YKK463), *hmo1*Δ cells (YKK209, YKK459, and YKK464), and *hmo1*Δ*fpr1*Δ cells (YKK206, YKK458, and YKK469) by tetrad dissection.

*His3MX6* gene fragment was amplified from pFA6a-His3-MX6 [3] by PCR using TK10268-KK648. Several *RPL25* promoter-reporter (*ADE2*) strains were generated as follows. *RPL25* promoter was amplified from genomic DNA of H2451 by PCR using KK645-KK646 primers to generate wild-type *RPL25* promoter (*RPL25*p[WT]). A portion of *RPL25* promoter was similarly amplified by PCR using KK649-KK646 primers to generate *RPL25* promoter lacking UAS (*RPL25*p[ΔUAS]). Two fragments of *RPL25* promoter were similarly amplified by PCR using KK650-KK646, or KK645-KK651 primers. These fragments were fused by PCR using KK645-TK10273 primers to generate *RPL25* promoter lacking IVR (*RPL25*p[ΔIVR]). Each of three *RPL25* promoters (*RPL25*p[WT]*, RPL25*p[ΔUAS]*,* and *RPL25*p [ΔIVR]) was fused with a *His3MX6* gene fragment by PCR using TK10272-TK10273 primers. The fused fragments were integrated into the *ADE2* locus of YKK207 to generate YKK1054, YKK1056, and YKK1058, respectively.

YKK468 and YKK455 were transformed with a fragment amplified from pKM750 by PCR using KK1098-TK10078 primers, and was selected on SD–His to generate YKK1123 and YKK1125, strains expressing C-terminally PA-tagged Hmo1, respectively*.* YKK468 and YKK459 were transformed with a fragment amplified from pKM750 by PCR using KK1099-TK4807 primers, and was selected on SD–His to generate YKK1127 and YKK1129, strains expressing C-terminally PA-tagged Fpr1, respectively*.*

YKK206, YKK207, YKK208, and YKK209 were transformed with a fragment amplified from pM5791 by PCR using KK985-TK4738 primers, and was selected on SD–His to generate strains expressing C-terminally FLAG (3×)-tagged Fhl1, YKK1086, YKK1087, YKK1090, and YKK1091, respectively.

YKK208 was transformed with a fragment amplified from pM5791 by PCR using KK986-KK987 primers, and was selected on SD–His to generate YKK1085, a strain expressing C-terminally FLAG (3×)-tagged Ifh1. YKK1085 was crossed with YKK458 to generate YKK1100, YKK1102, YKK1104, and YKK1106, by tetrad dissection.

YKK461 was transformed with a fragment amplified from pKM750 by PCR using KK1054-KK1055 primers, and was selected on SD–His to generate a strain expressing C-terminally PA-tagged Sua7. This strain was crossed with YKK458 to generate YKK1115, YKK1117, YKK1119, and YKK1121, by tetrad dissection.

YKK742, a diploid strain generated by crossing H2451 and YKK206, was transformed with a DNA fragment amplified from pM5791 by PCR using KK587-KK588 primers, and was selected on SD–His to generate YKK776, a diploid strain containing FLAG (3×)-tag at the C-terminus of an *FAP1* genes. Subsequently, YKK776 was subjected to tetrad dissection to generate YKK792 and YKK794. YKK207 and YKK208 were transformed with a fragment amplified from pM5791 by PCR using KK967-KK968 primers, and was selected on SD–His to generate YKK1072 and YKK1074, strains expressing C-terminally FLAG (3×)-tagged Rap1, respectively.

The 6864 strain was crossed with YKK166 to generate YKK899 by tetrad dissection. The 25053 strain was transformed with a fragment amplified from pKM750 by PCR using KK1054-KK1055 primers, and was selected on SD–His to generate YKK867, a diploid strain with a *tor2*Δ*::kanMX6* allele and a *fpr1*Δ*::His3MX6* allele. YKK867 was transformed with a plasmid pKM731 (*TOR2*/pRS316), and was subjected to tetrad dissection to generate YKK940. YKK940 was crossed with YKK899 to generate YKK1081 and YKK1080 by tetrad dissection. The *HMO1* gene of YKK1081 and YKK1080 was disrupted by transforming a DNA fragment amplified from pFA6a-His3MX6 by PCR using TK4022-TK4023 primers to generate YKK1093 and YKK1097, respectively.

YKK1087 was transformed with a plasmid pMK200 (NBRP ID: BYP7569; Masato Kanemaki) digested by *Stu*I, and was selected on SD–uracil to generate YKK1214, a host strain for rapid Fpr1-depletion experiment using AID-degron system.

**Construction of plasmids**

(i) Plasmid for integrating PA-tag into genes on chromosome

The pFA6a-3HA-His3MX6 [3] was modified by inverse PCR mutagenesis using KK860-KK861 primers to generate pKM750, a plasmid for integrating PA-tag at C-terminal region of genes on chromosome.

(ii) Plasmids expressing *HMO1*

A DNA fragment containing C-terminally FLAG (3×) tagged *HMO1* was amplified from pM2782 [1] by PCR using M13F-M13R primers. This DNA fragment was co-transformed into YKK97 together with the *Bam*HI-*Xho*I digested pRS316 to fuse these DNA fragments by homologous recombination in yeast cell. A resulting plasmid was recovered from colonies that grew on SD–uracil plate as pKM457.

(iii) Plasmids expressing *FPR1* or its orthologs derived from other species

A DNA fragment containing *FPR1* gene was amplified from genomic DNA of H2451 by PCR using KK92-KK93 primers, digested by *Bam*HI and *Pst*I, and then inserted into the *Bam*HI/*Pst*I site of pRS315 to generate pKM304. A PA-tag was inserted into N-terminus of *FPR1* on the pKM304 by inverse PCR mutagenesis using KK834-KK835 primers to generate pKM738. A DNA fragment containing N-terminally PA-tagged *FPR1* was prepared from pKM738 by *Bam*HI/*Pst*I digestion, and then inserted into the *Bam*HI/*Pst*I site of pM5140, a plasmid containing aureobasidin A resistant gene *AUR1-C* [1], to generate pKM740. The plasmid pKM738 was modified by inverse PCR mutagenesis using KK879-KK880 primers to generate pKM769, a plasmid expressing Fpr1-F43Y mutant protein. Similarly, pKM738 was modified by inverse PCR mutagenesis using KK970-KK971 primers to generate pKM788, a plasmid expressing Fpr1-Y89D mutant protein.

The entire region of pKM738 plasmid except for ORF of *FPR1* was amplified by PCR using KK887-KK889 primers. A DNA fragments containing *S. pombe fkh1^+^* gene was amplified from FYC25, a plasmid containing cDNA of *fkh1^+^* gene (provided by the National Bio-Resource Project (NBRP), Japan, http://yeast.nig.ac.jp/yeast/top.xhtml), by PCR using KK888-KK890 primers. These two DNA fragments were fused by Gibson assembly Master Mix (New ENGLAND BioLabs) to generate pKM774, a plasmid expressing N-terminally PA-tagged Fkh1 from *FPR1* promoter in *S. cerevisiae*. DNA fragments containing promoter or terminator of *FPR1* gene were amplified from pKM304 by PCR using KK92-KK572 and KK573-KK93 primers, respectively. A DNA fragment containing ORF of human *FKBP1A* was amplified from qPCR Human reference cDNA (TAKARA BIO) by PCR using KK571-KK574 primers. These three DNA fragments were fused by PCR using KK92-KK93 primers, digested by *Bam*HI and *Pst*I, and then inserted into the *Bam*HI/*Pst*I site of pRS315 to generate pKM607, a plasmid expressing FKBP1A from *FPR1* promoter in *S. cerevisiae*. The pKM607 was modified by inverse PCR mutagenesis using KK835-KK982 primers to generate pKM794, a plasmid expressing N-terminally PA-tagged FKBP1A. DNA fragments containing promoter and terminator of *FPR1* gene were amplified from pKM607 by PCR using KK92-KK963 and KK964-KK93, respectively. A DNA fragment containing ORF of human *FKBP1B* was amplified from qPCR Human reference cDNA by PCR using KK962-KK965 primers. These three DNA fragments were fused by PCR using KK92-KK93 primers, digested by *Bam*HI and *Pst*I, and then inserted into the *Bam*HI/*Pst*I site of pRS315 to generate pKM785, a plasmid expressing FKBP1B from *FPR1* promoter in *S. cerevisiae*. A DNA fragment containing promoter and a region encoding 1-31 aa of Fpr1 was amplified from pKM738 by PCR using T7-KK998 primers. A DNA fragment containing terminator of *FPR1* and a region encoding 26-112 aa of Fkh1 was amplified from pKM774 by PCR using KK997-M13R primers. These two DNA fragments were fused by PCR using T7-M13R primers, digested by *Eag*I and *Pst*I, and then inserted into the *Eag*I/*Pst*I site of pRS315 to generate pKM795, a plasmid expressing Fpr1-Fkh1 chimeric protein.

KK1217 and KK1218 primers were mixed and subjected to PCR reaction. Similarly, KK1219 and KK1220 primers were mixed and subjected to PCR reaction. The resulting two DNA fragments were fused by PCR using KK1185-KK1186 primers to generate a DNA fragment encoding mini AID degron tag whose codon usage was optimized for *S. cerevisiae.* DNA fragments containing promoter and terminator of *FPR1* gene were amplified from pKM304 by PCR using KK92-KK1221 and KK1222-KK93, respectively. These fragments were mixed with the mini AID fragment, and fused by PCR using KK92-KK93 primers, digested by *Bam*HI and *Pst*I, and then inserted into the *Bam*HI/*Pst*I site of pRS315 to generate pKM830, a plasmid expressing C-terminally AID-tagged Fpr1.

A DNA fragment containing ORF of *FPR1* gene was amplified from pKM304 by PCR using KK183-KK184 primers, digested by *Nco*I and *Xho*I, and then inserted into the *Nco*I/*Xho*I site of pET28b to generate pKM250, a plasmid expressing C-terminally His-tagged Fpr1 in *E. coli*.

(iv) Plasmids expressing *TOR2*

A DNA fragment containing *TOR2* gene was amplified from genomic DNA of BY4741 by PCR using KK755-KK756 primers. This fragment was inserted into the *Pst*I/*Xho*I site of pRS316 (whose another *Pst*I site was mutated) by Gibson assembly Master mix to generate pKM731.

The pKM731 was modified by inverse PCR mutagenesis using KK895-KK896 primers to generate pKM777, a plasmid expressing N-terminally PA-tagged Tor2. The fragment digested from pKM777 by *Pst*I and *Xho*I was inserted into the *Pst*I/*Xho*I site of pRS415 to generate pKM782. The pKM782 was subjected to inverse PCR mutagenesis using KK988-KK989, KK990-KK991, or KK992-KK993 to generate pKM802 (*tor2-S1975I*), pKM804 (*tor2-W2041L*), and pKM806 (*tor2-F2049L*), respectively.

**Oligonucleotide primers for ChIP assay**

**Fig 1:** The primer pairs used for amplification of *RPS5*p, *RPS5*c, *RPL25*p, *RPL25*c, *RPL10*p, *RPL10*c, and promoter of 35S rRNA gene in Fig 1A are, TK9174-TK9292, TK11284-TK11285, KK613-KK614, KK615-KK616, TK11299-TK11300, KK625-KK626, and TK9075-TK9076, respectively. The primer pairs used for amplification of promoter of *RPL25,* *RPS5*, 35S rRNA gene, *GCN4*, *SEC59*, *MSC6*, *TEF1*, and *PNS1* in Fig 1D are, KK640-KK641, TK9174-TK9292, TK9075-TK9076, KK942-KK943, KK944-KK945, KK946-KK947, KK948-KK949, and KK950-KK951, respectively.

**Fig 2:** The primer pairs used for amplification of different regions of *RPL25* (**a**, **b**, **c**, **d**, **e**, **f**, **g**, and **h** in Fig 2A) are, KK611-KK637, KK638-KK639, KK640-KK641, KK642-KK614, KK643-KK644, KK537-KK1047, KK1093-KK1094, and KK615-KK616, respectively. The primer pair used for amplification of upstream region of *RPL25* promoter-reporter (**i** in Fig 2B) is KK647-KK652. The primer pairs used to test for Fpr1-binding to UAS of *RPS5*, *RPL25*, and *RPS30B* in Fig 2C are KK983-TK10065, KK640-KK641, and KK1026-KK1027, respectively. The primer pairs used to test for Hmo1-binding to IVR of *RPS5*, *RPL25*, and *RPS30B* in Fig 2C are TK9174-TK9292, KK642-KK614, and KK1026-KK1027, respectively.

**Fig 3**: The primer pairs used to test for Fhl1/Ifh1-binding to promoters of *RPS5*, *RPL25*, *RPS25A*, *RPL18A*, *GCN4*, and *TEF1* in Fig 3B are TK9174-TK9292, KK640-KK641, TK10675-TK10676, KK1106-KK1107, KK942-KK943, and KK948-KK949, respectively.

**Fig 4**: The primer pairs used for amplification of different regions of *RPS5* (**j**, **k**, and **l** in Fig 4C), or *RPS25A* (**m**, **n**, **o**, and **p**) were, TK9734-TK10066, TK11310-KK984, TK493-TK9269, KK1091-KK1092, TK10675-KK1090, KK1086-KK1087, and KK1041-KK1042, respectively. The primers to amplify region s of *RPL25* (**e**, **f**, and **g**) are described above.

**Fig 5**: The primer pair used for amplification of *RPL25* promoter in Fig 5D and 5F is KK640-KK641.

**Fig 7**: The primer pairs used for amplification of promoters of *RPL25*, *RPL16B*, and *RPL2A* in Fig 7B are KK640-KK641, KK1059-KK1060, and KK1061-KK1062, respectively.

**S1 Fig**: The primer pair used for amplification of *RPL25* promoter in S1 Fig is KK640-KK641.

**S4 Fig**: The primer pairs used to test for Fhl1-binding to promoters of *RPL17A*, *RPL16A*, *RPL31B*, *RPS24B*, *RPS16B*, *RPS0A*, *RPS30B*, *RPL35B*, *RPL10*, *RPL41B*, *RPL22B*, and *RPL11B* in S4 Fig are KK1035-KK1036, KK1033-KK1034, KK1068-KK1069, KK1070-KK1071, KK1074-KK1075, KK1072-KK1073, KK1026-KK1027, KK1029-KK1030, TK11299-TK11300, KK1154-KK1155, KK1156-KK1157, and KK1158-KK1159, respectively.

**S5 Fig:** The primer pairs used to test for Fhl1-binding to promoters of *RPS25A*, *RPS30B*, and *RPS5* in S5 Fig are KK10675-KK10676, KK1026-KK1027, and TK9174-TK9292, respectively.

**S7 Fig:** The primer pairs used to test for binding of Fpr1 and Fhl1 to promoters of *RPS25A*, *RPS30B*, and *RPS5* in S7 Fig are KK10675-KK10676, KK1026-KK1027, and TK9174-TK9292, respectively.

**Reference**

**1.** Kasahara K, Ohtsuki K, Ki S, Aoyama K, Takahashi H, Kobayashi T, et al. Assembly of regulatory factors on rRNA and ribosomal protein genes in Saccharomyces cerevisiae. Mol Cell Biol. 2007; 27(19):6686-6705. <https://doi.org/MCB.00876-07> [pii]

10.1128/MCB.00876-07. PMID: 17646381.

**2.** Higashino A, Shiwa Y, Yoshikawa H, Kokubo T, Kasahara K. Both HMG boxes in Hmo1 are essential for DNA binding in vitro and in vivo. Biosci Biotechnol Biochem. 2015; 79(3):384-393. <https://doi.org/10.1080/09168451.2014.978258>. PMID: 25410521.

**3.** Longtine MS, McKenzie A, 3rd, Demarini DJ, Shah NG, Wach A, Brachat A, et al. Additional modules for versatile and economical PCR-based gene deletion and modification in Saccharomyces cerevisiae. Yeast. 1998; 14(10):953-961. PMID: 9717241.

**4.** Kasahara K, Ohyama Y, Kokubo T. Hmo1 directs pre-initiation complex assembly to an appropriate site on its target gene promoters by masking a nucleosome-free region. Nucleic Acids Res. 2011; 39(10):4136-4150. <https://doi.org/gkq1334> [pii]

10.1093/nar/gkq1334. PMID: 21288884.
